# Effects of Infant Pneumococcal Conjugate Vaccination on Serotype Distribution in Invasive Pneumococcal Disease among Children and Adults in Germany

**DOI:** 10.1371/journal.pone.0131494

**Published:** 2015-07-01

**Authors:** Mark van der Linden, Gerhard Falkenhorst, Stephanie Perniciaro, Matthias Imöhl

**Affiliations:** 1 National Reference Center for Streptococci, Department of Medical Microbiology, University Hospital (RWTH), Aachen, Germany; 2 Department for Infectious Disease Epidemiology, Robert Koch Institute, Berlin, Germany; Centers for Disease Control & Prevention, UNITED STATES

## Abstract

This study describes the effects of the introduction of universal infant pneumococcal conjugate vaccination in 2006 on invasive pneumococcal disease (IPD) among children and adults in Germany with a focus on the dynamics of serotype distribution in vaccinated and non-vaccinated age groups. Over a period of 22 years (1992–2014), microbiological diagnostic laboratories from all over Germany have been sending isolates of IPD cases to the German National Reference Center for Streptococci on a voluntary basis. *Streptococcus pneumoniae* isolates were serotyped using Neufeld’s Quellung method. Among children <16 years, the proportion of PCV7 serotypes among isolates from IPD cases decreased from 61.8% before vaccination (1997–2006) to 23.5% in the early vaccination period (2007–2010; p = 1.30E-72) and sank further to 5.2% in the late vaccination period (2010–2014; p = 4.59E-25). Similar reductions were seen for the separate age groups <2 years, 2-4 years and 5-15 years. Among adults, the proportion of PCV7 serotypes decreased from 43.4% in the pre-vaccination period (1992–2006) to 24.7% (p = 3.78E-88) in the early vaccination period and 8.2% (p = 5.97E-161) in the late vaccination period. Both among children and among adults, the non-PCV7 serotypes 1, 3, 7F and 19A significantly increased in the early vaccination period. After the switch from PCV7 to PVC10/PCV13 for infant vaccination in 2010, serotypes 1, 6A and 7F significantly decreased. A decrease in serotype 19A was only observed in 2013–2014, as compared to 2010–2011 (children p = 4.16E-04, adults p = 6.98E-06). Among adults, serotype 3, which strongly increased in the early vaccination period (p = 4.44E-15), remained at a constant proportion in the late vaccination period. The proportion of non-PCV13 vaccine serotypes increased over the whole vaccination period, with serotypes 10A, 12F, 23B, 24F and 38 most significantly increasing among children and serotypes 6C, 12F, 15A, 22F and 23B increasing among adults. Eight years of childhood pneumococcal conjugate vaccination have had a strong effect on the pneumococcal population in Germany, both among the target group for vaccination as well as among older children and adults.

## Introduction

*Streptococcus pneumoniae* (pneumococcus) is a major cause of infectious disease worldwide. Globally, over a million children and half a million adults die every year as a result of invasive pneumococcal disease (IPD), including meningitis, sepsis and bacteremic pneumonia [1]. Non-invasive conditions like non-bacteremic pneumonia and otitis media further increase the global burden of disease. Apart from being a pathogen, pneumococci are asymptomatically carried in the nasopharynx, by up to 50% of children under the age of 2 years, and also by 2–5% of adults [2, 3]. It is commonly assumed that colonization precedes disease, and that transmission occurs from child to child and from children to (older) adults. However, circulation of pneumococci among adults or transmission from adults to children is also possible [4]. The main burden of invasive and non-invasive pneumococcal disease lies in young children (0–5 years of age) and older adults (>50 years of age).

The introduction of vaccination aims to reduce the burden of pneumococcal disease, and the currently available vaccines are all directed against the pneumococcal capsular polysaccharides. The polysaccharide capsule is the primary virulence factor of *Streptococcus pneumoniae* and currently over 93 different capsular types (serotypes) have been described [5].

The 23-valent pneumococcal polysaccharide vaccine (Merck, Pneumovax 23, PPV23) contains polysaccharides of serotypes 1, 2, 3, 4, 5, 6B, 7F, 8, 9N, 9V, 10A, 11A, 12F, 14, 15B, 17F, 18C, 19F, 19A, 20, 22F, 23F, and 33F. This vaccine is licenced for ages 2 years and older, therefore, it cannot be used to reduce the burden of pneumococcal disease in young children. In the year 2000, a 7-valent pneumococcal conjugate vaccine was launched (Wyeth/Pfizer, Prevenar/Prevnar, PCV7) containing serotypes 4, 6B, 9V, 14, 18C, 19F and 23F. PCV7 consists of capsular polysaccharides which are conjugated to a carrier protein (CRM197). In 2009, two higher-valent pneumococcal conjugate vaccines were introduced: a 10-valent vaccine (GSK, Synflorix, PCV10), which includes the PCV7 serotypes plus serotypes 1, 5 and 7F, and a 13-valent vaccine (Wyeth/Pfizer, Prevenar13, PCV13), which includes the PCV10 serotypes plus serotypes 3, 6A and 19A. PCV13 uses the same carrier protein as PCV7 (CRM197) whereas Synflorix uses *Haemophilus influenzae* protein D as a carrier protein for 8 serotypes, tetanus toxoid for serotype 18C and diphtheria toxoid for serotype 19F. PCVs elicit a protein immune response and result in immunological memory, even in young children. Apart from systemic immunity against pneumococcal disease, PCVs also induce mucosal immunity, which prevents carriage of the included serotypes.

Many countries have implemented pneumococcal vaccination programs for infants and older adults. Whereas vaccination programs for adults (with PPV23) have resulted in relatively low vaccination rates [6], vaccination programs for conjugate childhood vaccination have been very successful [7, 8]: in vaccinated children, the programs have resulted in a marked reduction of both IPD and carriage of serotypes included in the vaccine. Furthermore, they have resulted in a reduction in vaccine-serotype IPD among older children and adults, a phenomenon called herd protection [9]. Herd protection results from a reduced vaccine-serotype carriage among vaccinated individuals who then have a lowered chance of transferring these serotypes to non-vaccinated individuals.

Parallel to the reduction in serotypes included in the conjugate vaccines, an increase in IPD caused by non-vaccine serotypes (replacement) was observed in many countries. However, in most countries this increase was much lower than the decrease in vaccine serotype disease [10].

A recommendation for pneumococcal conjugate vaccination for all children under 2 years of age was issued by the German standing committee on vaccination (STIKO) in July 2006 [11]. Germany does not have a national immunization program, but vaccination costs of recommended vaccinations are reimbursed by health insurance companies. PCV7 was licensed in Germany in 2001, followed by PCV10 in April 2009 and PCV13 in December 2009 (replacing PCV7).

The actual choice of vaccine made by the parents/physicians is reflected in the prescription data of different vaccine formulations for children under two years of age in Germany. Before 2009, only PCV7 was used. In 2009, 19.8% of prescriptions were PCV10, in 2010 this lowered to 5.6%, in 2011 to 2.6% and in 2014 only 1.9% of prescriptions were PCV10 (data from IMS Health, Germany, ‘Verordnungsindex Pharma (VIP)’).

For adults aged 60 years and older, the 23-valent polysaccharide vaccine (PPV23, SPMSD, Pneumovax) has been recommended since 1998, currently as a single dose. A further vaccination recommendation exists for all children and adults with an increased risk for pneumococcal disease due to underlying conditions. For children up to the age of four years, pneumococcal conjugate vaccine is recommended; for individuals older than 5 years, a vaccination with PCV13 or PPV23 is recommended [12].

The uptake of pneumococcal conjugate vaccine among children under two years of age in Germany is 80–85% (as based on sold/prescribed doses) [13–16]. Among adults aged ≥65 years PPV23 vaccination levels have been low in Germany (31%, [17]).

This study describes the effects of the introduction of childhood pneumococcal conjugate vaccination on invasive pneumococcal disease among children and adults in Germany, focusing on the dynamics of serotype distributions in vaccinated and non-vaccinated age groups over a period of 22 years.

## Materials and Methods

### Study materials

The German National Reference Center for Streptococci (GNRCS) has conducted surveillance for IPD in Germany since 1992, using a laboratory-based approach. IPD cases were defined as *Streptococcus pneumoniae* isolates from blood, cerebrospinal fluid (CSF) or any other normally sterile body fluid. Microbiological diagnostic laboratories from all over Germany have been sending isolates of IPD cases to the GNRCS on a voluntary basis. In total, over 400 laboratories have participated, including large, nationally-operating commercial labs. Participating laboratories are located in all German federal states, and the number of laboratories per federal state correlates to the different population densities of the states. In the last 7 years of the study (2007–2008 to 2013–2014), reported cases varied between 0.7 per 100,000 inhabitants per year (Schleswig-Holstein) and 5.6 per 100,000 per year (Bremen) (Table in S1Table).

Over the years, the surveillance system has been improved. In 2001, surveillance for adults was enhanced in North Rhine-Westphalia (22% of German population), as well as in Bavaria and Saxony in 2006. On each occasion, all laboratories in the respective federal states were approached and asked to send in isolates. In 2007, a web-based surveillance system called PneumoWeb (www.rki.de/pneumoweb) was set up by the Robert Koch Institute in collaboration with the GNRCS. PneumoWeb enables the laboratories to report a case of IPD via an online system, and directly print the corresponding information as a Case Report Form to send to the GNRCS, accompanied by the IPD isolate. The web-based system resulted in a large increase in reported cases for adults, whereas the amount of cases for children remained at the same high level. For children, using our capture-recapture incidence calculations, we determined that before the vaccination recommendation, 40–50% of all IPD cases had a sample sent to the GNRCS. This percentage increased to 50–60% after vaccination introduction [18].

### Characterization of isolates and serotyping

Species identification was performed using bile and optochin testing. In dubious cases, PCR analysis of several genes was performed (*ply*, *lytA*, *sodA*, 16S-rRNA). As a last resort, MLST was performed. Pneumococcal isolates were serotyped by Neufeld’s Quellung reaction using type and factor sera provided by the Statens Serum Institut, Copenhagen, Denmark. Isolates were considered non-typeable when there was no reaction with any of the antisera.

### Statistical methods

Cases were grouped per pneumococcal season (from July to June of consecutive years) because of known infection clusters during winter. For the analysis of vaccination effects, we defined three time periods. The pre-vaccination period from 1997–2006 summarizes 9 pneumococcal seasons in which children were not vaccinated (for adults: 1992–2006, 14 seasons). The season 2006–2007 was considered a transition year in which pneumococcal conjugate vaccination was introduced, and was taken out of the analysis. The early vaccination period summarized the three seasons (2007–2008, 2008–2009 and 2009–2010) in which PCV7 was used, and the late vaccination period summarizes four seasons (2010–2011, 2011–2012, 2012–2013 and 2013–2014) in which higher-valent vaccines (mainly PCV13) were used. To study the most recent effects of higher-valent vaccination, a direct comparison of the seasons 2010–2011 and 2013–2014 was made.

Differences in proportions were tested by Fisher´s exact test with a two-sided p-value <0.05 considered statistically significant. Analyses were conducted using R (R Foundation for Statistical Computing, Vienna, Austria, 2014).

### Ethical statement

An ethical approval was not required since the study was performed with *Streptococcus pneumoniae* isolates that resulted from routine microbiological diagnostic procedures as requested by the treating physician. No additional biological specimens were taken for the purpose of this study. Specimens were anonymized and only data on year and month of birth, sex, type of specimen and hospital/laboratory where the case was diagnosed were registered.

## Results

**Fig 1:**
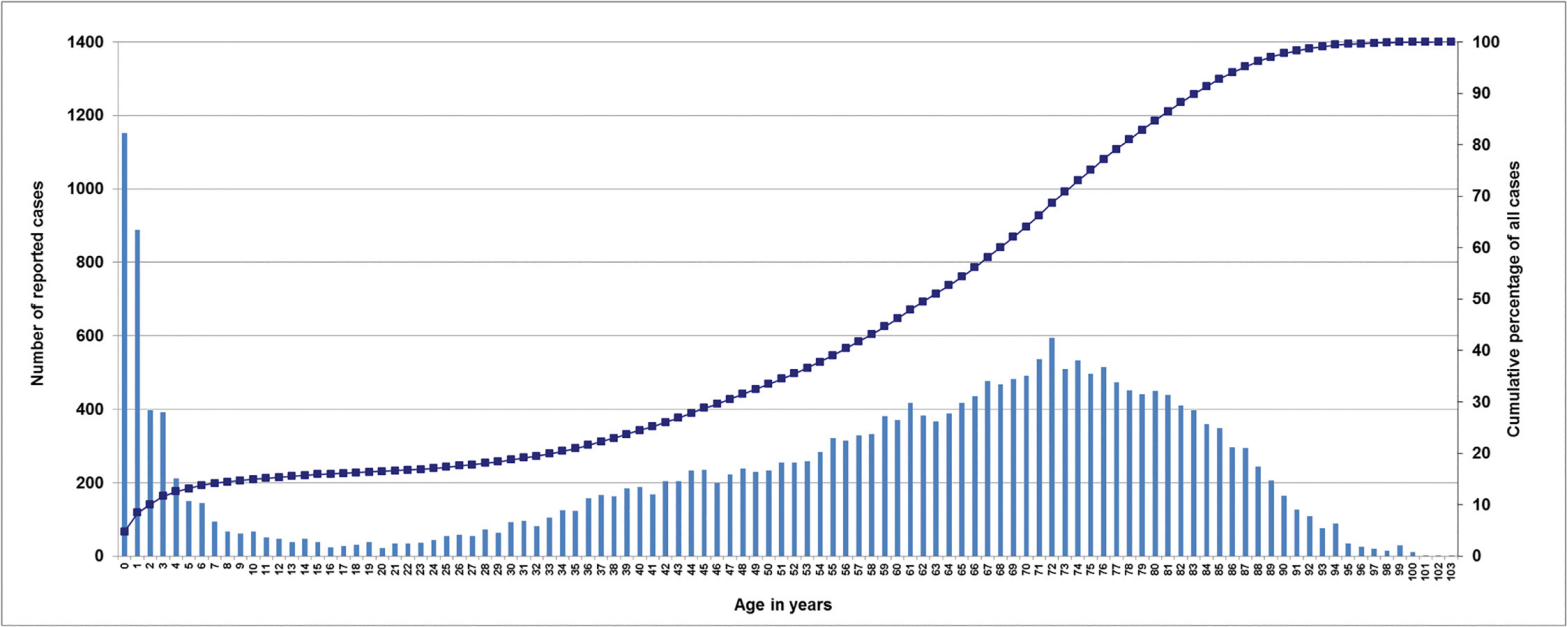
Age distribution of reported IPD isolates (1992–2014) in Germany (n = 24,235). Left y-axis: number of reported cases, right y-axis cumulative percentage of all cases. Data represent the age distribution of isolates sent to the GNRCS on a voluntary basis. Since the surveillance system was improved several times (see materials and methods section), the graph does not necessarily represent the actual prevalence of IPD in the different age groups in Germany.

**Fig 2:**
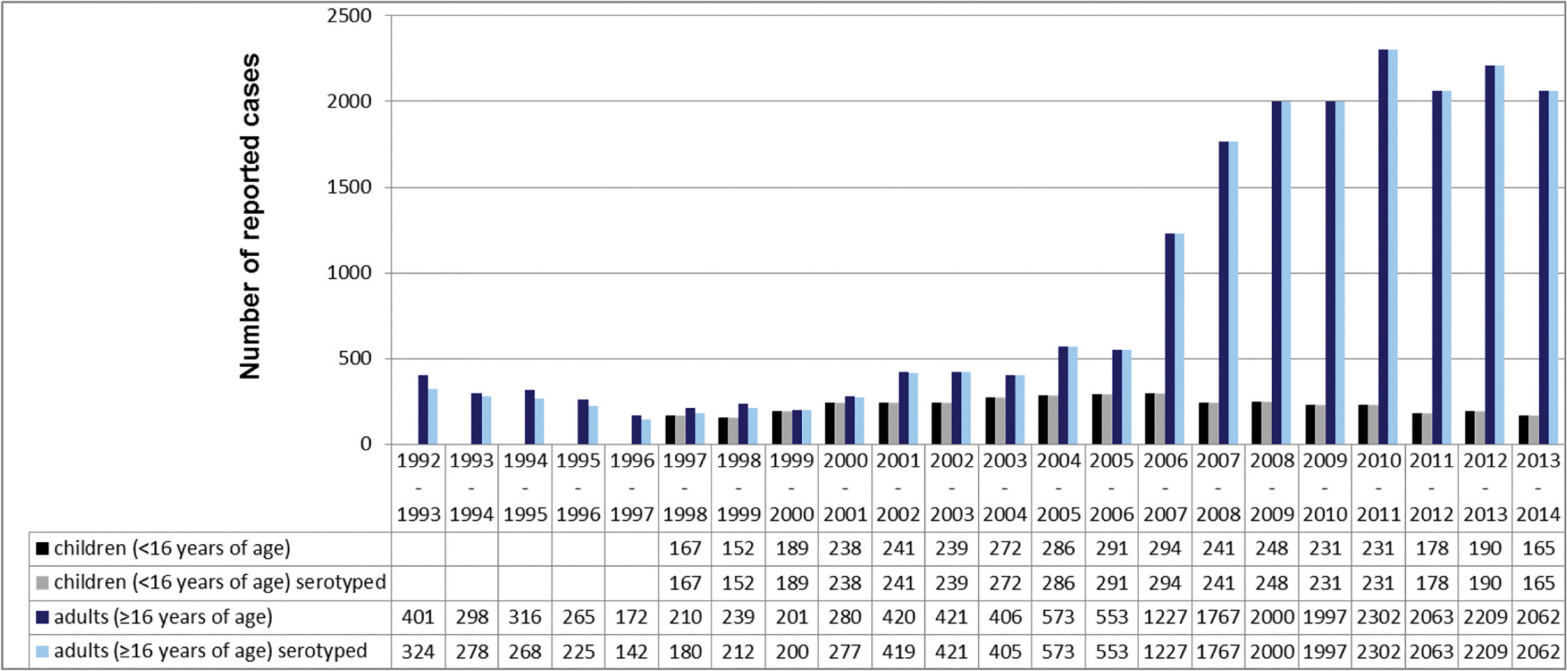
Numbers of reported IPD isolates among children (n = 3,853, all serotyped) and adults (n = 20,382, serotyped: n = 20,104) in Germany.

From July 1992 until June 2014, a total of 3,853 isolates from invasive pneumococcal disease (IPD) among children (<16 years) and 20,382 isolates from IPD among adults (≥16 years) were received at the GNRCS. Of all isolates (24,235), 15.9% were from children under 16 years of age (11.7% from children under 5 years of age). 68.5% of the isolates were from adults over 50, 53.8% from adults over 60 years of age (Fig 1). The median age among isolates from children was 1 year (21 months), and 57.3% of isolates were from male patients (40.0% female, 2.7% gender unknown). Among adults the median age was 67 years, and 45.3% of isolates were from male patients (54.2% female, 0.5% gender unknown). All isolates from children were serotyped, whereas 20,104 isolates from adults were available for serotyping (Fig 2).

### Effects of PCV on IPD among children

In the pre-vaccination period, PCV7 serotypes represented an average of 61.8% of all isolates among children. This proportion was reduced to 23.5% in the early vaccination period (p = 1.30E-72) and sank further to 5.2% in the late vaccination period (p = 4.59E-25). The reduction in the early vaccination period was highly significant for all PCV7 serotypes except for serotype 18C (p = 0.21). Reductions between the early and late vaccination period were much less significant, mainly due to the already very low numbers (Table 1). The reduction of serotype 18C reached statistical significance in the late vaccination period (p = 4.36E-05). Similar reductions were seen for the separate age groups <2 years, 2–4 years and 5–15 years, though reductions in the two higher age groups are less significant due to lower numbers (Tables A-D in S1Text). Comparison of the season 2010–2011 with 2013–2014 shows that PCV7 serotypes have almost disappeared in all age groups.

**Table 1:** Serotype distribution among isolates from IPD in children (0–15 years of age) in Germany (n = 3,853).

Serotype	Pre-Vaccination 1997–2006		Early Vaccination 2007–2010		Late Vaccination 2010–2014		Pre-Vaccination vs. Early Vaccination		Early Vaccination vs. Late Vaccination		2010–2011		2013–2014		2010–2011 vs. 2013–2014	
	n	%	n	%	n	%	p-value	direction	p-value	direction	n	%	n	%	p-value	direction
4	78	3.8	7	1.0	0	0.0	**6.92E-05**	**decreasing**	**6.23E-03**	**decreasing**	0	0.0	0	0.0	1.00	
6B	156	7.5	25	3.5	7	0.9	**7.05E-05**	**decreasing**	**9.58E-04**	**decreasing**	3	1.3	1	0.6	6.44E-01	decreasing
9V	208	10.0	24	3.3	3	0.4	**1.60E-09**	**decreasing**	**1.58E-05**	**decreasing**	1	0.4	1	0.6	1.00	
14	437	21.1	40	5.6	6	0.8	**3.11E-25**	**decreasing**	**4.08E-08**	**decreasing**	3	1.3	0	0.0	2.69E-01	decreasing
18C	116	5.6	31	4.3	7	0.9	2.08E-01	decreasing	**4.36E-05**	**decreasing**	4	1.7	3	1.8	1.00	
19F	157	7,6	31	4.3	16	2.1	**2.40E-03**	**decreasing**	**1.73E-02**	**decreasing**	6	2.6	3	1.8	7.41E-01	decreasing
23F	130	6,3	11	1.5	1	0.1	**5.10E-08**	**decreasing**	**2.59E-03**	**decreasing**	0	0.0	0	0.0	1.00	
PCV7	1282	61.8	169	23.5	40	5.2	**1.30E-72**	**decreasing**	**4.59E-25**	**decreasing**	17	7.4	8	4.8	4.03E-01	decreasing
1	151	7.3	114	15.8	77	10.1	**1.24E-10**	**increasing**	**1.09E-03**	**decreasing**	39	16.9	6	3.6	**2.08E-05**	**decreasing**
5	12	0.6	4	0.6	1	0.1	1.00		2.05E-01	decreasing	1	0.4	0	0.0	1.00	
7F	119	5.7	96	13.3	65	8.5	**3.82E-10**	**increasing**	**3.38E-03**	**decreasing**	28	12.1	5	3.0	**1.36E-03**	**decreasing**
PCV10	1564	75.4	383	53.2	183	24.0	**1.26E-27**	**decreasing**	**2.15E-31**	**decreasing**	85	36.8	19	11.5	**8.42E-09**	**decreasing**
PCV10non7	282	13.6	214	29.7	143	18.7	**7.77E-21**	**increasing**	**8.01E-07**	**decreasing**	68	29.4	11	6.7	**8.75E-09**	**decreasing**
3	57	2.7	41	5.7	46	6.0	**3.86E-04**	**increasing**	8.26E-01	increasing	12	5.2	12	7.3	4.02E-01	increasing
6A	72	3.5	28	3.9	6	0.8	6.41E-01	increasing	**7.41E-05**	**decreasing**	1	0.4	0	0.0	1.00	
19A	58	2.8	54	7.5	77	10.1	**2.05E-07**	**increasing**	8.27E-02	increasing	35	15.2	7	4.2	**4.16E-04**	**decreasing**
PCV13	1751	84.4	506	70.3	312	40.8	**1.50E-15**	**decreasing**	**1.96E-30**	**decreasing**	133	57.6	38	23.0	**6.34E-12**	**decreasing**
PCV13non7	469	22,6	337	46.8	272	35.6	**3.04E-33**	**increasing**	**1.18E-05**	**decreasing**	116	50.2	30	18.2	**3.60E-11**	**decreasing**
PCV13non10	187	9.0	123	17,1	129	16.9	**1.21E-08**	**increasing**	9.45E-01	decreasing	48	20.8	19	11.5	**2.03E-02**	**decreasing**
2	0	0.0	0	0.0	0	0.0	1.00		1.00		0	0.0	0	00	1.00	
8	16	0.8	6	0.8	9	1.2	8.11E-01	increasing	6.08E-01	increasing	1	0.4	4	2.4	1.65E-01	increasing
9N	19	0.9	7	1.0	8	1.0	8.25E-01	increasing	1.00		2	0.9	2	1.2	1.00	
10A	32	1.5	29	4.0	42	5.5	**2.78E-04**	**increasing**	2.24E-01	increasing	8	3.5	11	6.7	1.58E-01	increasing
11A	7	0.3	8	1.1	18	2.4	**3.18E-02**	**increasing**	7.63E-02	increasing	3	1.3	5	30	2.86E-01	increasing
12F	8	0.4	8	1.1	33	4.3	**4.02E-02**	**increasing**	**1.83E-04**	**increasing**	5	2.2	9	5.5	9.97E-02	increasing
15B	14	0.7	16	2.2	23	3.0	**1.22E-03**	**increasing**	4.17E-01	increasing	3	1.3	7	4.2	1.01E-01	increasing
17F	5	0.2	4	0.6	2	0.3	2.48E-01	increasing	4.39E-01	decreasing	0	0.0	2	1.2	1.73E-01	increasing
20	4	0.2	1	0.1	0	0.0	1.00		4.85E-01	decreasing	0	0.0	0	0.0	1.00	
22F	14	0.7	15	2.1	26	3.4	**2.51E-03**	increasing	1.53E-01	increasing	5	2.2	5	3.0	7.47E-01	increasing
33F	11	0.5	9	1.3	23	3.0	6.84E-02	increasing	2.04E-02	increasing	4	1.7	8	4.8	1.34E-01	increasing
PPV23	1809	87.2	581	80.7	490	64.1	**3.62E-05**	**decreasing**	**1.17E-12**	**decreasing**	163	70.6	91	55.2	**2.02E-03**	**decreasing**
9A	20	1.0	0	0.0	0	0.0	**3.72E-03**	**decreasing**	1.00		0	0.0	0	0.0	1.00	
15C	14	0.7	17	2.4	35	4.6	**5.90E-04**	**increasing**	2.33E-02	increasing	11	4.8	11	6.7	5.06E-01	increasing
18B	14	0.7	0	0.0	0	0.0	**2.75E-02**	**decreasing**	1.00		0	0.0	0	0.0	1.00	
23B	4	0.2	3	0.4	34	4.5	3.83E-01	increasing	**1.30E-07**	**increasing**	10	4.3	4	2.4	4.12E-01	decreasing
24F	32	1.5	24	3.3	58	7.6	**5.02E-03**	**increasing**	**3.72E-04**	**increasing**	16	6.9	15	9.1	4.52E-01	increasing
34	3	0.1	2	0.3	7	0.9	6.08E-01	increasing	1.80E-01	increasing	0	0.0	5	3.0	**1.21E-02**	**increasing**
35B	2	0.1	4	0.6	13	1.7	**4.16E-02**	**increasing**	**4.94E-02**	**increasing**	5	2.2	3	1.8	1.00	
38	12	0.6	15	2.1	38	5.0	**1.20E-03**	**increasing**	**3.02E-03**	**increasing**	10	4.3	8	4.8	8.11E-01	increasing
nonPCV13	324	15.6	214	29.7	452	59.2	**1.50E-15**	**increasing**	**1.96E-30**	**increasing**	98	42.4	127	77.0	**6.34E-12**	**increasing**
total	2075	100.0	720	100.0	764	100.0	** **	** **	** **	** **	231	100.0	165	100.0	** **	** **

Of the six extra serotypes included in PCV13 as compared to PCV7, four showed an increase in the early vaccination period: 1 (p = 1.24E-10), 3 (p = 3.86E-04), 7F (p = 3.82E-10) and 19A (p = 2.05E-07). The proportion of serotype 6A increased, but not significantly, whereas the number of serotype 5 isolates was too low for analysis. In the late vaccination period, a significant decrease was observed for serotypes 1, 6A and 7F. Serotypes 3 and 19A further increased, but the increase was no longer statistically significant. The increase in serotypes 19A and 3 persisted well into the late vaccination period. A decrease in serotype 19A was only observed in 2013–2014, as compared to 2010–2011 (p = 4.16E-04). However, when looking at the separate age groups, the impact of the higher-valent vaccination becomes very clear among children <2 years of age, with each of the six extra serotypes decreasing (Tables A-D in S1Text).

The proportion of non-PCV13 vaccine serotypes among IPD in children in the pre-vaccination period was 15.6%. This proportion increased to 29.7% in the early vaccination period, and to 59.2% in the late vaccination period. The most significantly increasing serotypes were 10A, 12F, 23B, 24F and 38. Table 1 lists only those non-PCV13 serotypes which showed statistically significant changes. Table A in S1Text lists all non-PCV13 serotypes.

### Effects of PCV on IPD among adults

PCV7 serotypes represented 43.4% of all isolates among adults in the pre-vaccination period (1992–2006). This proportion decreased to 24.7% (p = 3.78E-88) in the early vaccination period, and to 8.2% (p = 5.97E-161) in the late vaccination period. The reduction was highly significant for all serotypes except for 19F (p = 0.095) in the early vaccination period, and became highly significant for all serotypes in the late vaccination period (Table 2). Looking at separate age groups (16–49 years, 50–60 years, 61–75 years and >75 years), similar reductions were seen, with the lowest reductions for serotypes 18C and 19F (Tables E-I in S1Text).

**Table 2:** Serotype distribution among isolates from IPD in adults (>15 years of age) in Germany (n = 20,104).

Serotype	Pre-Vaccination 1992–2006		Early Vaccination 2007–2010		Late Vaccination 2010–2014		Pre-Vaccination vs. Early Vaccination		Early Vaccination vs. Late Vaccination		2010–2011		2013–2014		2010–2011 vs. 2013–2014	
	n	%	n	%	n	%	p-value	direction	p-value	direction	n	%	n	%	p-value	direction
4	368	8.2	288	5.0	164	1.9	**6.15E-11**	**decreasing**	**7.61E-25**	**decreasing**	59	2.6	31	1.5	**1.42E-02**	**decreasing**
6B	183	4.1	110	1.9	49	0.6	**6.99E-11**	**decreasing**	**9.38E-14**	**decreasing**	16	0.7	11	0.5	5.65E-01	decreasing
9V	286	6.4	203	3.5	66	0.8	**2.40E-11**	**decreasing**	**1.12E-32**	**decreasing**	32	1.4	4	0.2	**5.35E-06**	**decreasing**
14	591	13.2	367	6.4	133	1.5	**1.09E-31**	**decreasing**	**2.05E-53**	**decreasing**	41	1.8	23	1.1	7.73E-02	decreasing
18C	105	2.3	101	1.8	78	0.9	**3.94E-02**	**decreasing**	**1.02E-05**	**decreasing**	33	1.4	14	0.7	**1.82E-02**	**decreasing**
19F	153	3.4	163	2.8	131	1.5	9.47E-02	decreasing	**9.63E-08**	**decreasing**	54	2.3	23	1.1	**2.53E-03**	**decreasing**
23F	257	5.7	194	3.4	90	1.0	**8.47E-09**	**decreasing**	**3.33E-22**	**decreasing**	38	1.7	13	0.6	**1.72E-03**	**decreasing**
PCV7	1943	43.4	1426	24.7	711	8.2	**3.78E-88**	**decreasing**	**5.97E-161**	**decreasing**	273	11.9	119	5.8	**1.18E-12**	**decreasing**
1	307	6.9	487	8.4	413	4.8	**2.87E-03**	**increasing**	**1.35E-18**	**decreasing**	187	8.1	54	2.6	**4.16E-16**	**decreasing**
5	35	0.8	26	0.5	4	0.0	**3.77E-02**	**decreasing**	**1.75E-07**	**decreasing**	3	0.1	0	0.0	2.52E-01	decreasing
7F	296	6.6	592	10.3	788	9.1	**5.02E-11**	**increasing**	**2.25E-02**	**decreasing**	294	12.8	98	4.8	**4.14E-21**	**decreasing**
PCV10	2581	57.7	2531	43.9	1916	22.2	**2.30E-43**	**decreasing**	**2.29E-166**	**decreasing**	757	32.9	271	13.1	**8.67E-55**	**decreasing**
PCV10non7	638	14.3	1105	19.2	1205	14.0	**3.91E-11**	**increasing**	**1.15E-16**	**decreasing**	484	21.0	152	7.4	**5.37E-39**	**decreasing**
3	389	8.7	785	13.6	1213	14.0	**4.44E-15**	**increasing**	4.76E-01	increasing	319	13.9	310	15.0	2.81E-01	increasing
6A	140	3.1	174	3.0	118	1.4	7.73E-01	decreasing	**1.19E-11**	**decreasing**	31	1.3	22	1.1	4.11E-01	decreasing
19A	128	2.9	349	6.1	839	9.7	**7.51E-15**	**increasing**	**1.98E-15**	**increasing**	252	10.9	145	7.0	**6.98E-06**	**decreasing**
PCV13	3238	72.3	3839	66.6	4086	47.3	**5.24E-10**	**decreasing**	**1.90E-116**	**decreasing**	1359	59.0	748	36.3	**2.16E-51**	**decreasing**
PCV13non7	1295	28.9	2413	41.9	3375	39.1	**5.21E-42**	**increasing**	**8.68E-04**	**decreasing**	1086	47.2	629	30.5	**1.36E-29**	**decreasing**
PCV13non10	657	14.7	1308	22.7	2170	25.1	**6.97E-25**	**increasing**	**8.42E-04**	**increasing**	602	26.2	477	23.1	**2.23E-02**	**decreasing**
2	4	0.1	8	0.1	0	0.0	5.68E-01	increasing	**6.57E-04**	**decreasing**	0	0.0	0	0.0	1.00	
8	160	3.6	171	3.0	349	4.0	9.10E-02	decreasing	**7.29E-04**	**increasing**	63	2.7	109	5.3	**1.62E-05**	**increasing**
9N	119	2.7	178	3.1	317	3.7	2.13E-01	increasing	6.19E-02	increasing	70	3.0	92	4.5	**1.59E-02**	**increasing**
10A	80	1.8	131	2.3	256	3.0	9.24E-02	increasing	**1.16E-02**	**increasing**	59	2.6	76	3.7	**3.55E-02**	**increasing**
11A	101	2.3	126	2.2	232	2.7	8.39E-01	decreasing	6.32E-02	increasing	48	2.1	54	2.6	2.70E-01	increasing
12F	120	2.7	112	1.9	505	5.8	**1.33E-02**	**decreasing**	**1.28E-32**	**increasing**	84	3.6	174	8.4	**2.03E-11**	**increasing**
15B	24	0.5	63	1.1	97	1.1	**2.25E-03**	**increasing**	9.35E-01	increasing	25	1.1	19	0.9	6.50E-01	decreasing
17F	29	0.6	16	0.3	65	0.8	**6.23E-03**	**decreasing**	**1.42E-04**	**increasing**	14	0.6	17	0.8	4.71E-01	increasing
20	38	0.8	41	0.7	72	0.8	4.28E-01	decreasing	4.42E-01	increasing	23	1.0	18	0.9	7.54E-01	decreasing
22F	103	2.3	272	4.7	597	6.9	**4.73E-11**	**increasing**	**4.34E-08**	**increasing**	157	6.8	161	7.8	2.21E-01	increasing
33F	35	0.8	65	1.1	150	1.7	8.51E-02	increasing	**3.15E-03**	**increasing**	44	1.9	42	2.0	8.28E-01	increasing
PPV23	3911	87.4	4848	84.1	6608	76.5	**3.38E-06**	**decreasing**	**5.00E-29**	**decreasing**	1557	67.6	1488	72.2	**1.70E-18**	**decreasing**
6C	32	0.7	99	1.7	261	3.0	**4.89E-06**	**increasing**	**6.26E-07**	**increasing**	60	2.6	50	2.4	7.72E-01	decreasing
9A	21	0.5	5	0.1	1	0.0	**1.94E-04**	**decreasing**	**4.11E-02**	**decreasing**	0	0.0	0	0.0	1.00	
12A	6	0.1	3	0.1	13	0.2	1.92E-01	decreasing	1.23E-01	increasing	0	0.0	7	0.3	**5.23E-03**	**increasing**
12B	1	0.0	4	0.1	0	0.0	3.94E-01	increasing	**2.57E-02**	**decreasing**	0	0.0	0	0.0	1.00	
13	20	0.4	9	0.2	5	0.1	**7.83E-03**	**decreasing**	9.80E-02	decreasing	1	0.0	0	0.0	1.00	
15A	27	0.6	31	0.5	216	2.5	6.92E-01	decreasing	**1.48E-21**	**increasing**	30	1.3	75	3.6	**4.85E-07**	**increasing**
15C	19	0.4	24	0.4	70	0.8	1.00		**4.13E-03**	**increasing**	15	0.7	20	1.0	3.08E-01	increasing
16F	20	0.4	25	0.4	84	1.0	1.00		**2.46E-04**	**increasing**	12	0.5	24	1.2	**2.79E-02**	**increasing**
23A	31	0.7	95	1.6	215	2.5	**1.13E-05**	**increasing**	**6.55E-04**	**increasing**	46	2.0	59	2.9	7.45E-02	increasing
23B	6	0.1	31	0.5	231	2.7	**6.70E-04**	**increasing**	**3.76E-24**	**increasing**	31	1.3	75	3.6	**9.32E-07**	**increasing**
24F	47	1.0	78	1.4	204	2.4	1.74E-01	increasing	**1.57E-05**	**increasing**	28	1.2	78	3.8	**3.18E-08**	**increasing**
31	24	0.5	36	0.6	93	1.1	6.03E-01	increasing	**4.97E-03**	**increasing**	18	0.8	23	1.1	2.74E-01	increasing
35B	3	0.1	32	0.6	105	1.2	**8.37E-06**	**increasing**	**4.77E-05**	**increasing**	17	0.7	34	1.6	**6.81E-03**	**increasing**
35F	25	0.6	91	1.6	142	1.6	**6.67E-07**	**increasing**	7.88E-01	increasing	29	1.3	42	2.0	5.45E-02	increasing
38	26	0.6	63	1.1	96	1.1	**5.23E-03**	**increasing**	9.35E-01	increasing	17	0.7	25	1.2	1.22E-01	increasing
nonPCV13	1239	27.7	1925	33.4	4550	52.7	**5.24E-10**	**increasing**	**1.90E-116**	**increasing**	943	41.0	1314	63.7	**2.16E-51**	**increasing**
total	4477	100.0	5764	100.0	8636	100.0	** **	** **	** **	** **	2302	100.0	2062	100.0		

Of the PCV13-non-PCV7 serotypes, 1, 3, 7F and 19A increased in the early vaccination period, whereas 6A decreased, but only the increase in 7F reached statistical significance (1.96E-02). In the late vaccination period, serotypes 1, 6A and 7F significantly decreased. Serotype 19A only showed a decrease (p = 6.98E-06) in 2013–2014 (as compared to 2010–2011). Serotype 3, which strongly increased in the early vaccination period (p = 4.44E-15), remained at a constant prevalence in the late vaccination period.

Non-PCV13 serotypes made up 27.7% of all isolates in the pre-vaccination period, increasing to 33.4% in the early vaccination period, to 52.7% in the late vaccination period and to 63.7% in the last season (2013–2014). The most significantly increasing serotypes among adults were 6C, 12F, 15A, 22F and 23B. Table 2 lists only those non-PCV13 serotypes which showed statistically significant changes. Table B in S1Text lists all non-PCV13 serotypes.

### Serotype 19A

Among children <2 years of age, cases with serotype 19A have increased in the early vaccination period (p = 3.03E-08). During the late vaccination period, 19A decreased as compared to the early vaccination period, but not significantly (p = 9.07E-01). A significant decrease in 19A cases was only observed when comparing 2010–2011 to 2013–2014 (p = 1.06E-03). Among children 2–4 years of age, 19A cases increased significantly in both the early and the late vaccination period, and a (non-significant) decrease was only seen in the last surveillance years (2010–2011 vs. 2013–2014; p = 2.36E-01). Among older children (5–15 years of age), neither the vaccination increase in 19A cases nor the decrease in the latter surveillance years reached statistical significance (Tables A-D in S1Text). Among adults, the dynamics of serotype 19A were similar: a significant increase in all age groups during both the early and the late vaccination periods. Only when comparing 2010–2011 to 2013–2014 a significant decrease in reported cases with serotype 19A was observed for all age groups except for the 16–49 year olds, where the decrease did not reach significance (p = 4.27E-01; Tables E-I in S1Text).

### Serotype 3

In the early vaccination period, reported cases with serotype 3 significantly increased in all age groups, except for children 5–15 years of age, among whom a non-significant decrease was observed (p = 8.36E-01). In the late vaccination period, and also when comparing 2010–2011 to 2013–2014, no significant changes in serotype 3 levels were observed in any age group (Tables A-I in S1Text).

### Serotype 1

The number of reported cases with serotype 1 increased in the early vaccination period in each separate age group, reaching statistical significance among children 0–1 years and 2–4 years, and among adults 16–49 years and 50–60 years. In the late vaccination period, a steep decrease in the number of reported serotype 1 cases was observed among all age groups, most significantly among 16–49 year olds (p = 2.51E-07) and >75 year olds (p = 9.42E-07) (Tables A-I in S1Text).

### PPV23 serotypes among adults

In the pre-vaccination period, PPV23 serotypes were responsible for 87.4% of all IPD cases reported from adults (Table 2) with little variation in this percentage over the 14 prevaccination seasons (84.2%- 90.6%). In the early vaccination period, this percentage decreased significantly to 84.1% (p = 3.38E-06), due to the significant decrease in PCV7 serotypes (p = 3.78E-88). The decrease became more significant in the late vaccination period (76.5%, p = 5.00E-29), caused by the continuing decrease in PCV7 serotypes (p = 5.97E-161) and the decrease in PCV13-non-PCV7 serotypes (p = 8.68E-04). Several of the serotypes included in PPV23 but not in PCV13 significantly increased, either over the whole vaccination period (22F, 33F), or only in the late vaccination period (8, 12F).

### Serotypes never detected

We have not detected any of the following 28 serotypes in IPD cases among children in Germany: 6D, 7B, 7C, 10C, 11C, 11D, 11E, 11F, 16A, 17A, 22A, 25A, 25F, 32A, 32F, 33C, 33D, 40, 41A, 41F, 42, 43, 44, 45, 46, 47A, 47F, 48. Of these 28 serotypes, the following 17 serotypes have not been found among adult IPD cases either: 10C, 11D, 11E, 16A, 32A, 32F, 33C, 33D, 40, 41A, 41F, 42, 43, 44, 46, 47A, 47F.

### Serotypes in transition season 2006–2007

Two serotypes were found only once and only in the transition season 2006–2007. This concerned a case of serotype 10F in a child and a case of the new serotype 6G (see below) in an adult.

### New serotypes

In the course of this study, two new serogroup 6 serotypes (6F and 6G) were discovered, which have been described by Melissa Oliver and our group: [19].

### Dynamics of serotype distribution over time

**Fig 3:**
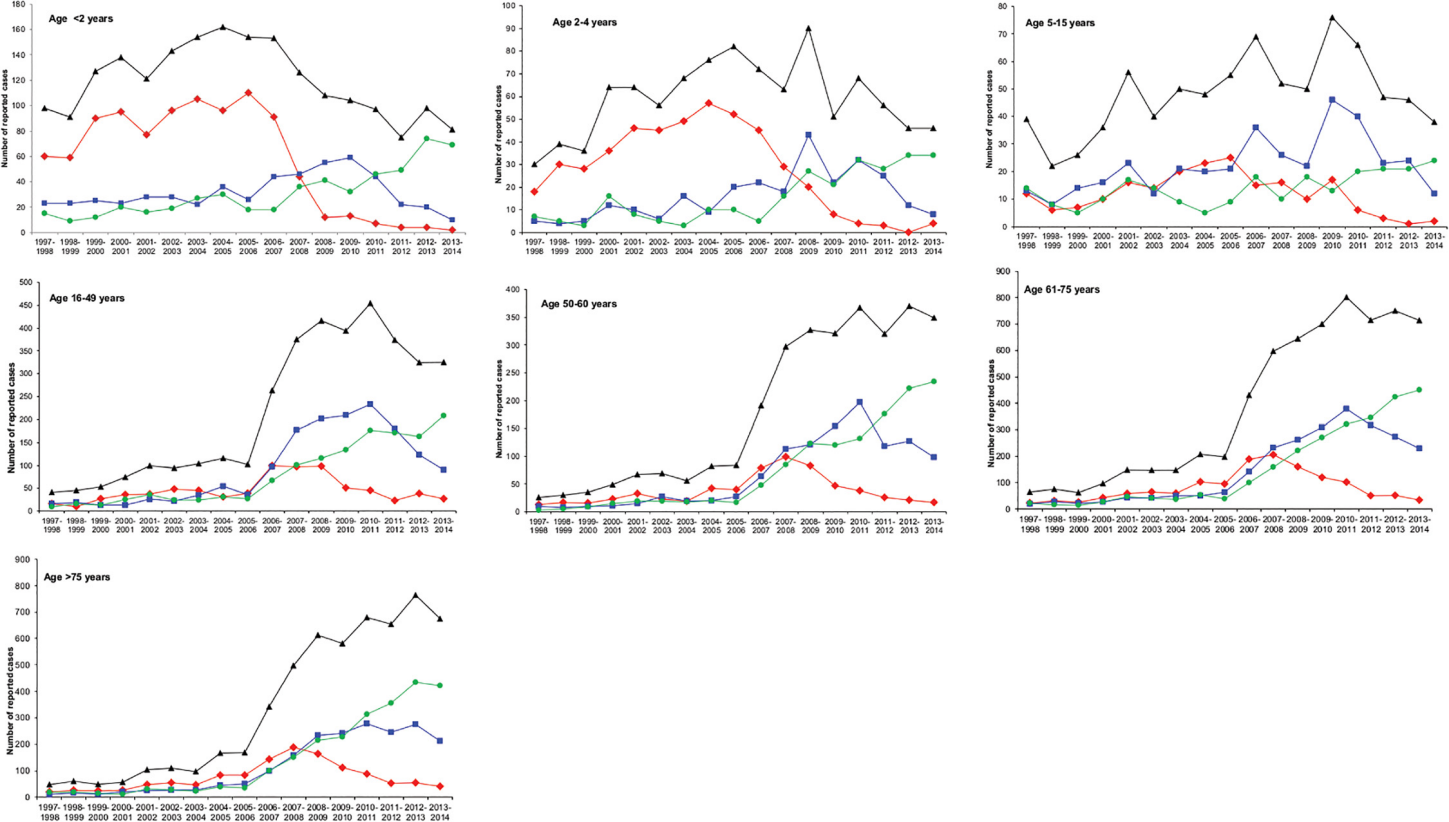
Isolates from IPD cases in Germany from 1997/1998 to 2013/2014 according to age groups and PCV7 vaccine-type (red), PCV13-non-PCV7 vaccine-type (blue) or non-PCV13 vaccine-type (green). The total number of IPD cases is shown in black.

The development over time of the serotype distribution for seven different age groups is presented in Fig 3. Immediately after the start of PCV7 vaccination, a steep drop in reported cases with PCV7 serotypes was observed for children <2 years of age, resulting in almost no reported PCV7 cases in 2013–2014. Among 2–4 year olds, a similar decrease was observed, although it was slower. Among children aged 5–15 years, a decrease in PCV7 serotypes was only observed starting in 2010–2011. In all three age groups, the PCV13-non-PCV7 serotypes increased in the early vaccination period, but decreased in the late vaccination period. Non-PCV13 serotypes increased among all three childhood age groups, but to a lesser extent among older children (5–16 years of age). The increase in non-PCV13 serotypes was most pronounced for children <2 years and from 2011–2014. The total amount of reported IPD cases has decreased in all childhood age groups in the vaccination period.

Among adults, a reduction of PCV7 serotypes was observed in all four age groups starting from 2008–2009. PCV13-non-PCV7 serotypes increased considerably in all age groups in the early vaccination period, and decreased again in the late vaccination period. The decrease was much less pronounced among older adults (>75 years of age). Cases with non-PCV13 serotypes have increased in all four adult age groups and during the whole vaccination period (2007–2014). A reduction in the total amount of reported IPD cases was not observed in any of the four adult age groups (2007–2014).

**Fig 4:**
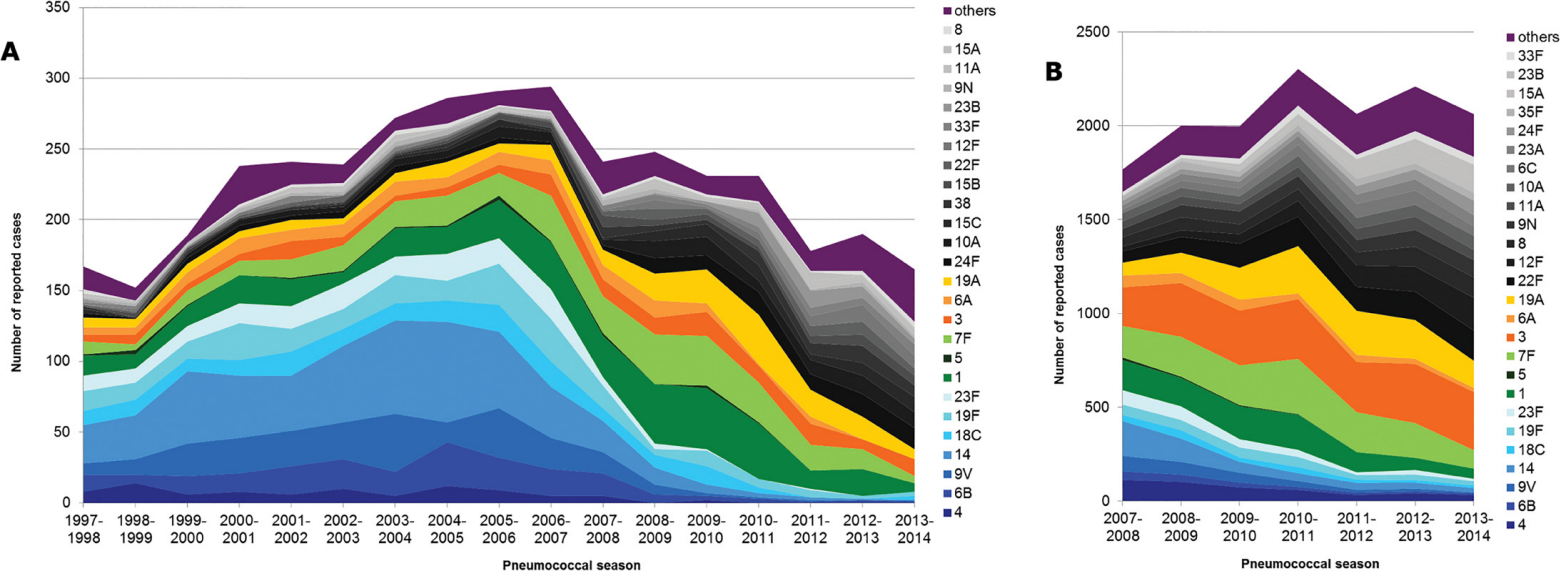
Serotype distribution of IPD cases among children (1997–2014, A) and adults (2007–2014, B) in Germany. Blue: PCV7 serotypes, Green: PCV10-PCV7 serotypes, Orange: PCV13-PCV10 serotypes, Black/Gray: major non-PCV13 serotypes, Purple: other non-PCV13 serotypes.

Fig 4A shows the dynamics of the individual serotypes among children over the surveillance period (1997–2014). In the early vaccination period, PCV7 serotypes (blue) decreased, whereas PCV13-non-PCV7 serotypes (green and orange), as well as non-PCV13 serotypes (black, grey and purple), increased. In the late vaccination period, PCV13-non-PCV7 serotypes decreased, while the non-PCV13 serotypes continued to increase. The amount of reported cases per season has clearly decreased from 2006–2007 to 2013–2014. In Fig 4B, the dynamics of the serotype distribution in the vaccination period (2007–2014) among adults is presented. Here, PCV7 serotypes lost importance as well, while PCV13-non-PCV7 serotypes increased in the early vaccination period and decreased in the late vaccination period. Non-PCV13 serotypes continuously increased over the whole vaccination period (2007–2014). A reduction in the number of reported cases per pneumococcal season is not observed.

## Discussion

Our study has several limitations. Since IPD is not a notifiable disease in Germany, isolates were sent in by clinical microbiological laboratories on a voluntary basis, which bears the risk of under-reporting. Furthermore, the systematic sampling of invasive isolates from adults (1992) and children (1997) was taken up at different times, and for adults, the surveillance included population-based studies in three German federal states: North Rhine-Westphalia, started in 2001; Bavaria, started in 2006; Saxony, started in 2006. Over the years, the surveillance project has been continuously intensified, particularly after the recommendation for universal infant vaccination against pneumococci was issued. This recommendation unavoidably led to an increased awareness of IPD among clinical microbiologists and pediatricians. Finally, in 2007, the introduction of PneumoWeb, a web-based reporting system contributed to an increased number of reported cases of IPD among adults, from 200–500 cases per season to over 2000 cases per season. The introduction of PneumoWeb did not lead to increased reporting among children, reflecting the good level of reporting already reached in previous years.

The impact of pneumococcal conjugate vaccination on IPD caused by PCV7 serotypes has been vast, with PCV7 serotypes having almost disappeared among children <2 years of age. Similarly, PCV7 serotypes have been strongly reduced among older children and adults, indicating herd protection. These effects are in accordance with reductions of IPD incidence among children published by our group [20] and with reports from other countries that have introduced pneumococcal conjugate vaccination [21]. Among the PCV13-non-PCV7 serotypes, serotypes 1, 3, 7F and 19A increased in the early vaccination period (in all age groups), when PCV7 was used. These serotypes apparently occupied the niche vacated by the PCV7 serotypes. This replacement was observed in other countries as well [10]. After the introduction of higher-valent vaccination, serotypes 1, 7F and 6A decreased in all age groups. This immediate effect of higher-valent vaccination was also observed in other countries [22–25]. The steep rise in serotype 19A cases, which occurred in the early vaccination period, was only reverted in 2013–2014. Effects on serotype 19A have been more direct in other countries [26]. An explanation for the delayed effect in Germany could be the late onset of the increase in 19A after the start of PCV7 vaccination [27]. Therefore, when higher-valent vaccines were introduced, 19A was still steeply increasing, and it took time for this increase to first diminish and then be reversed. Obviously, the decrease in non-targeted age groups appeared with even more delay.

A decrease of serotype 3 cases was observed among children <2 years of age, in the late vaccination period, but it did not reach significance. Case numbers of serotype 3 among children are very low, and therefore it is difficult to judge whether there is a vaccination effect. However, serotype 3 cases did not increase, which might have been expected if the vaccine had no effect on serotype 3 at all. Interestingly, a herd protection effect towards the older age groups was not observed either. Among adults, serotype 3 has increased strongly and is now the most prevalent serotype. Steens *et al*. report very low numbers of serotype 3 cases among children <2 years in Norway, and also did not observe an effect of PCV13 on serotype 3 in non-targeted age groups [24]. Harboe *et al*. report no change in serotype 3 following PCV13 vaccination in Denmark [25]. Miller *et al*. report a non-significant reduction of serotype 3 since PCV13 introduction, whereas Kaplan *et al*. report a 68% decrease in serotype 3 among patients from eight US children’s hospitals, although numbers were low [28].

The significant increase of non-PCV serotypes shows that replacement is an issue after each PCV introduction, even though the net effect of vaccination remains positive. Several of the upcoming serotypes in this study (6C, 10A, 12F, 15A, 22F, 23B, 24F and 38), have been described in other studies as well [24, 25]. The most strongly increasing serotypes, 23B and 15A, were also found to be increasing in Norway [24] and in Hong Kong [29], respectively.

The immediate, strong and lasting decrease in serotype 1 cases in all age groups in the late vaccination period is enigmatic. For other serotypes, the herd protection effects among non-targeted age-groups came with a delay in time of about one year. For serotype 1, the observed reduction was as fast as in the directly-vaccinated age groups. A reduction in serotype 1 was not observed among adults in Denmark [25] but it was seen in Norway, and appears to have occurred just as fast [24].

In the early vaccination period, an immediate decrease of the PCV7 serotypes was observed among children <2 years of age. Among 2–4 year old children, an immediate decrease was also seen, but it was slower. Among older children (5–15 years), and among adults, the decrease came with a considerable delay (2010–2011 and 2008–2009, respectively). This shows that herd protection often comes into effect with a time delay. For the PCV13-non-PCV7 serotypes, an increase in the early vaccination period, followed by a decrease in the late vaccination period was observed in all age groups. Again, the decrease occurred with a delay in the non-vaccinated age groups. Interestingly, the decrease was less strong among the oldest adults (>75 years).

The share of PPV23 serotypes among adult IPD cases remained the same over the entire prevaccination period, indicating little effect of PPV23 vaccination. This could either be due to limited effectiveness of the PPV23 vaccine, or to low levels of vaccination among adults in Germany (31%, [17]). The PPV23 serotype share changed slightly in the early vaccination period as compared to pre-vaccination times. This was due to the fact that all of the replacement serotypes (1, 3, 7F, 19A) were included in PPV23. In the late vaccination period, the share of PPV23 serotypes decreased sharply, as now only some of the replacement serotypes were included in PPV23 (8, 12F, 22F, 33F), whereas others were not (6C, 15A, 23B).

It is of interest that a total of 17 serotypes have never been detected during our 22 years of surveillance. Obviously, serotype 11E cannot be distinguished from 11A using antisera and therefore was not detected in our study [30]. The remaining 16 serotypes, however, are so rare that they are hardly ever (if at all) reported in any surveillance studies. The current epidemiological relevance of these serotypes therefore remains unclear.

In the course of this study, two new serotypes (6F and 6G) were detected [19]. Both isolates show point mutations in the capsular genes, resulting in new variants of the capsular polysaccharide. Whether these serotypes arose as a consequence of vaccination pressure remains unclear, since one of the variants already appeared in 2006, i.e. before the start of vaccination in Germany.

## Conclusions

Eight years of childhood pneumococcal conjugate vaccination have had a strong effect on the pneumococcal population in Germany, both among vaccinated children as well as among non-vaccinated children and adults. Serotypes included in the vaccines have strongly diminished, but have not disappeared completely. Non-vaccine serotypes have gained importance, with several single serotypes occurring much more frequently than others. These phenomena stress the importance of continued surveillance in order to monitor the dynamics of the pneumococcal population under vaccination pressure and inform the development of higher-valent pneumococcal vaccines.

## Supporting Information

S1 Table Distribution of reporting laboratories and number of reported cases per federal state (2007–2008 to 2013–2014). (DOC)

S1 Text Serotype distributions among isolates from IPD in different age groups of children and adults in Germany. (DOC)
