# Supplementary material for: Effects of Infant Pneumococcal Conjugate Vaccination on Serotype Distribution in Invasive Pneumococcal Disease among Children and Adults in Germany
Source: PLoS One. 2015 Jul 1;10(7):e0131494. doi: 10.1371/journal.pone.0131494 (PMC4488910; doi:10.1371/journal.pone.0131494)
Supplement: S1 Table — (DOC) [file pone.0131494.s001.doc]

**Supporting Information**

**S1 Table: Distribution of reporting laboratories and number of reported cases per federal state (2007-2008 to 2013-2014).**

| **Federal State** | **Laboratories (n)** | **Isolates** | **Population** | **Laboratories per 100,000 inhabitants** | **Reported cases per 100,000 inhabitants** | **Reported cases per 100,000 inhabitants per pneumococcal season** |
| --- | --- | --- | --- | --- | --- | --- |
| Brandenburg | **23** | 497 | 2,449,511 | 0.94 | 20.3 | 2.9 |
| Berlin | **20** | 1,187 | 3,375,222 | 0.59 | 35.2 | 5.0 |
| Baden-Wurttemberg | **40** | 2,438 | 10,569,111 | 0.38 | 23.1 | 3.3 |
| Bavaria | **60** | 2,417 | 12,519,571 | 0.48 | 19.3 | 2.8 |
| Bremen | **5** | 257 | 654,774 | 0.76 | 39.3 | 5.6 |
| Hesse | **22** | 1,007 | 6,016,481 | 0.37 | 16.7 | 2.4 |
| Hamburg | **6** | 432 | 1,734,272 | 0.35 | 24.9 | 3.6 |
| Mecklenburg-Western Pomerania | **13** | 236 | 1,600,327 | 0.81 | 14.7 | 2.1 |
| Lower Saxony | **40** | 1,029 | 7,778,995 | 0.51 | 13.2 | 1.9 |
| North Rhine-Westphalia | **91** | 4,039 | 17,554,329 | 0.52 | 23.0 | 3.3 |
| Rhineland-Palatinate | **9** | 292 | 3,990,278 | 0.23 | 7.3 | 1.0 |
| Saxony-Anhalt | **10** | 321 | 2,806,531 | 0.36 | 11.4 | 1.6 |
| Saarland | **5** | 248 | 994,287 | 0.50 | 24.9 | 3.6 |
| Saxony | **43** | 1,050 | 4,050,204 | 1.06 | 25.9 | 3.7 |
| Schleswig-Holstein | **13** | 114 | 2,259,393 | 0.58 | 5.0 | 0.7 |
| Thuringia | **17** | 312 | 2,170,460 | 0.78 | 14.4 | 2.1 |
| **all** | **417** | **15,876** | **80,523,746** | **0.52** | **19.7** | **2.8** |

For most isolates the federal state of the patient was provided. Whenever this information was not available, the federal state of the laboratory was used, which could lead to imprecision in the case of nationally operating laboratories. Pneumococcal season: from July to June of consecutive year.
