# Supplementary material for: Effects of Infant Pneumococcal Conjugate Vaccination on Serotype Distribution in Invasive Pneumococcal Disease among Children and Adults in Germany
Source: PLoS One. 2015 Jul 1;10(7):e0131494. doi: 10.1371/journal.pone.0131494 (PMC4488910; doi:10.1371/journal.pone.0131494)
Supplement: S1 Text — (DOC) [file pone.0131494.s002.doc]

**Supporting Information**

**Table A: Serotype distribution among isolates from IPD in children (0-15 years of age) in Germany (n=3,853).**

| **Serotype** | **Pre-Vaccination 1997-2006** | | **Early Vaccination 2007-2010** | | **Late Vaccination 2010-2014** | | **Pre-Vaccination vs. Early Vaccination** | | **Early Vaccination vs. Late Vaccination** | | **2010-2011** | | **2013-2014** | | **2010-2011 vs. 2013-2014** | |
| --- | --- | --- | --- | --- | --- | --- | --- | --- | --- | --- | --- | --- | --- | --- | --- | --- |
|  | **n** | **%** | **n** | **%** | **n** | **%** | **p-value** | **direction** | **p-value** | **direction** | **n** | **%** | **n** | **%** | **p-value** | **direction** |
| 4 | 78 | 3.8 | 7 | 1.0 | 0 | 0.0 | **6.92E-05** | **decreasing** | **6.23E-03** | **decreasing** | 0 | 0.0 | 0 | 0.0 | 1.00 |  |
| 6B | 156 | 7.5 | 25 | 3.5 | 7 | 0.9 | **7.05E-05** | **decreasing** | **9.58E-04** | **decreasing** | 3 | 1.3 | 1 | 0.6 | 6.44E-01 | decreasing |
| 9V | 208 | 10.0 | 24 | 3.3 | 3 | 0.4 | **1.60E-09** | **decreasing** | **1.58E-05** | **decreasing** | 1 | 0.4 | 1 | 0.6 | 1.00 |  |
| 14 | 437 | 21.1 | 40 | 5.6 | 6 | 0.8 | **3.11E-25** | **decreasing** | **4.08E-08** | **decreasing** | 3 | 1.3 | 0 | 0.0 | 2.69E-01 | decreasing |
| 18C | 116 | 5.6 | 31 | 4.3 | 7 | 0.9 | 2.08E-01 | decreasing | **4.36E-05** | **decreasing** | 4 | 1.7 | 3 | 1.8 | 1.00 |  |
| 19F | 157 | 7.6 | 31 | 4.3 | 16 | 2.1 | **2.40E-03** | **decreasing** | **1.73E-02** | **decreasing** | 6 | 2.6 | 3 | 1.8 | 7.41E-01 | decreasing |
| 23F | 130 | 6.3 | 11 | 1.5 | 1 | 0.1 | **5.10E-08** | **decreasing** | **2.59E-03** | **decreasing** | 0 | 0.0 | 0 | 0.0 | 1.00 |  |
| PCV7 | 1282 | 61.8 | 169 | 23.5 | 40 | 5.2 | **1.30E-72** | **decreasing** | **4.59E-25** | **decreasing** | 17 | 7.4 | 8 | 4.8 | 4.03E-01 | decreasing |
| 1 | 151 | 7.3 | 114 | 15.8 | 77 | 10.1 | **1.24E-10** | **increasing** | **1.09E-03** | **decreasing** | 39 | 16.9 | 6 | 3.6 | **2.08E-05** | **decreasing** |
| 5 | 12 | 0.6 | 4 | 0.6 | 1 | 0.1 | 1.00 |  | 2.05E-01 | decreasing | 1 | 0.4 | 0 | 0.0 | 1.00 |  |
| 7F | 119 | 5.7 | 96 | 13.3 | 65 | 8.5 | **3.82E-10** | **increasing** | **3.38E-03** | **decreasing** | 28 | 12.1 | 5 | 3.0 | **1.36E-03** | **decreasing** |
| PCV10 | 1564 | 75.4 | 383 | 53.2 | 183 | 24.0 | **1.26E-27** | **decreasing** | **2.15E-31** | **decreasing** | 85 | 36.8 | 19 | 11.5 | **8.42E-09** | **decreasing** |
| PCV10non7 | 282 | 13.6 | 214 | 29.7 | 143 | 18.7 | **7.77E-21** | **increasing** | **8.01E-07** | **decreasing** | 68 | 29.4 | 11 | 6.7 | **8.75E-09** | **decreasing** |
| 3 | 57 | 2.7 | 41 | 5.7 | 46 | 6.0 | **3.86E-04** | **increasing** | 8.26E-01 | increasing | 12 | 5.2 | 12 | 7.3 | 4.02E-01 | increasing |
| 6A | 72 | 3.5 | 28 | 3.9 | 6 | 0.8 | 6.41E-01 | increasing | **7.41E-05** | **decreasing** | 1 | 0.4 | 0 | 0.0 | 1.00 |  |
| 19A | 58 | 2.8 | 54 | 7.5 | 77 | 10.1 | **2.05E-07** | **increasing** | 8.27E-02 | increasing | 35 | 15.2 | 7 | 4.2 | **4.16E-04** | **decreasing** |
| PCV13 | 1751 | 84.4 | 506 | 70.3 | 312 | 40.8 | **1.50E-15** | **decreasing** | **1.96E-30** | **decreasing** | 133 | 57.6 | 38 | 23.0 | **6.34E-12** | **decreasing** |
| PCV13non7 | 469 | 22.6 | 337 | 46.8 | 272 | 35.6 | **3.04E-33** | **increasing** | **1.18E-05** | **decreasing** | 116 | 50.2 | 30 | 18.2 | **3.60E-11** | **decreasing** |
| PCV13non10 | 187 | 9.0 | 123 | 17.1 | 129 | 16.9 | **1.21E-08** | **increasing** | 9.45E-01 | decreasing | 48 | 20.8 | 19 | 11.5 | **2.03E-02** | **decreasing** |
| 2 | 0 | 0.0 | 0 | 0.0 | 0 | 0.0 | 1.00 |  | 1.00 |  | 0 | 0.0 | 0 | 0.0 | 1.00 |  |
| 8 | 16 | 0.8 | 6 | 0.8 | 9 | 1.2 | 8.11E-01 | increasing | 6.08E-01 | increasing | 1 | 0.4 | 4 | 2.4 | 1.65E-01 | increasing |
| 9N | 19 | 0.9 | 7 | 1.0 | 8 | 1.0 | 8.25E-01 | increasing | 1.00 |  | 2 | 0.9 | 2 | 1.2 | 1.00 |  |
| 10A | 32 | 1.5 | 29 | 4.0 | 42 | 5.5 | **2.78E-04** | **increasing** | 2.24E-01 | increasing | 8 | 3.5 | 11 | 6.7 | 1.58E-01 | increasing |
| 11A | 7 | 0.3 | 8 | 1.1 | 18 | 2.4 | **3.18E-02** | **increasing** | 7.63E-02 | increasing | 3 | 1.3 | 5 | 3.0 | 2.86E-01 | increasing |
| 12F | 8 | 0.4 | 8 | 1.1 | 33 | 4.3 | **4.02E-02** | **increasing** | **1.83E-04** | **increasing** | 5 | 2.2 | 9 | 5.5 | 9.97E-02 | increasing |
| 15B | 14 | 0.7 | 16 | 2.2 | 23 | 3.0 | **1.22E-03** | **increasing** | 4.17E-01 | increasing | 3 | 1.3 | 7 | 4.2 | 1.01E-01 | increasing |
| 17F | 5 | 0.2 | 4 | 0.6 | 2 | 0.3 | 2.48E-01 | increasing | 4.39E-01 | decreasing | 0 | 0.0 | 2 | 1.2 | 1.73E-01 | increasing |
| 20 | 4 | 0.2 | 1 | 0.1 | 0 | 0.0 | 1.00 |  | 4.85E-01 | decreasing | 0 | 0.0 | 0 | 0.0 | 1.00 |  |
| 22F | 14 | 0.7 | 15 | 2.1 | 26 | 3.4 | **2.51E-03** | increasing | 1.53E-01 | increasing | 5 | 2.2 | 5 | 3.0 | 7.47E-01 | increasing |
| 33F | 11 | 0.5 | 9 | 1.3 | 23 | 3.0 | 6.84E-02 | increasing | 2.04E-02 | increasing | 4 | 1.7 | 8 | 4.8 | 1.34E-01 | increasing |
| PPV23 | 1809 | 87.2 | 581 | 80.7 | 490 | 64.1 | **3.62E-05** | **decreasing** | **1.17E-12** | **decreasing** | 163 | 70.6 | 91 | 55.2 | **2.02E-03** | **decreasing** |
| 6C | 9 | 0.4 | 3 | 0.4 | 11 | 1.4 | 1.00 |  | 5.77E-02 | increasing | 1 | 0.4 | 5 | 3.0 | 8.62E-02 | increasing |
| 7A | 0 | 0.0 | 1 | 0.1 | 0 | 0.0 | 2.58E-01 | increasing | 4.85E-01 | decreasing | 0 | 0.0 | 0 | 0.0 | 1.00 |  |
| 9A | 20 | 1.0 | 0 | 0.0 | 0 | 0.0 | **3.72E-03** | **decreasing** | 1.00 |  | 0 | 0.0 | 0 | 0.0 | 1.00 |  |
| 9L | 4 | 0.2 | 0 | 0.0 | 1 | 0.1 | 5.78E-01 | decreasing | 1.00 |  | 0 | 0.0 | 0 | 0.0 | 1.00 |  |
| 31 | 0 | 0.0 | 0 | 0.0 | 2 | 0.3 | 1.00 |  | 5.00E-01 | increasing | 0 | 0.0 | 1 | 0.6 | 4.17E-01 | increasing |
| 10B | 0 | 0.0 | 0 | 0.0 | 1 | 0.1 | 1.00 |  | 1.00 |  | 1 | 0.4 | 0 | 0.0 | 1.00 |  |
| 11B | 1 | 0.0 | 1 | 0.1 | 0 | 0.0 | 4.49E-01 | increasing | 4.85E-01 | decreasing | 0 | 0.0 | 0 | 0.0 | 1.00 |  |
| 12A | 3 | 0.1 | 0 | 0.0 | 2 | 0.3 | 5.74E-01 | decreasing | 5.00E-01 | increasing | 0 | 0.0 | 2 | 1.2 | 1.73E-01 | increasing |
| 12B | 2 | 0.1 | 1 | 0.1 | 0 | 0.0 | 1.00 |  | 4.85E-01 | decreasing | 0 | 0.0 | 0 | 0.0 | 1.00 |  |
| 13 | 2 | 0.1 | 1 | 0.1 | 0 | 0.0 | 1.00 |  | 4.85E-01 | decreasing | 0 | 0.0 | 0 | 0.0 | 1.00 |  |
| 15A | 18 | 0.9 | 4 | 0.6 | 10 | 1.3 | 6.24E-01 | decreasing | 1.80E-01 | increasing | 2 | 0.9 | 1 | 0.6 | 1.00 |  |
| 15C | 14 | 0.7 | 17 | 2.4 | 35 | 4.6 | **5.90E-04** | **increasing** | 2.33E-02 | increasing | 11 | 4.8 | 11 | 6.7 | 5.06E-01 | increasing |
| 15F | 1 | 0.0 | 0 | 0.0 | 0 | 0.0 | 1.00 |  | 1.00 |  | 0 | 0.0 | 0 | 0.0 | 1.00 |  |
| 16F | 4 | 0.2 | 4 | 0.6 | 5 | 0.7 | 2.16E-01 | increasing | 1.00 |  | 2 | 0.9 | 2 | 1.2 | 1.00 |  |
| 18A | 7 | 0.3 | 2 | 0.3 | 1 | 0.1 | 1.00 |  | 6.14E-01 | decreasing | 0 | 0.0 | 0 | 0.0 | 1.00 |  |
| 18B | 14 | 0.7 | 0 | 0.0 | 0 | 0.0 | **2.75E-02** | **decreasing** | 1.00 |  | 0 | 0.0 | 0 | 0.0 | 1.00 |  |
| 18F | 4 | 0.2 | 1 | 0.1 | 0 | 0.0 | 1.00 |  | 4.85E-01 | decreasing | 0 | 0.0 | 0 | 0.0 | 1.00 |  |
| 19B | 1 | 0.0 | 0 | 0.0 | 0 | 0.0 | 1.00 |  | 1.00 |  | 0 | 0.0 | 0 | 0.0 | 1.00 |  |
| 19C | 2 | 0.1 | 0 | 0.0 | 0 | 0.0 | 1.00 |  | 1.00 |  | 0 | 0.0 | 0 | 0.0 | 1.00 |  |
| 21 | 1 | 0.0 | 2 | 0.3 | 9 | 1.2 | 1.65E-01 | increasing | 6.57E-02 | increasing | 2 | 0.9 | 0 | 0.0 | 5.13E-01 | decreasing |
| 23A | 6 | 0.3 | 6 | 0.8 | 10 | 1.3 | 8.95E-02 | increasing | 4.55E-01 | increasing | 2 | 0.9 | 4 | 2.4 | 2.40E-01 | increasing |
| 23B | 4 | 0.2 | 3 | 0.4 | 34 | 4.5 | 3.83E-01 | increasing | **1.30E-07** | **increasing** | 10 | 4.3 | 4 | 2.4 | 4.12E-01 | decreasing |
| 24A | 1 | 0.0 | 0 | 0.0 | 0 | 0.0 | 1.00 |  | 1.00 |  | 0 | 0.0 | 0 | 0.0 | 1.00 |  |
| 24B | 0 | 0.0 | 1 | 0.1 | 1 | 0.1 | 2.58E-01 | increasing | 1.00 |  | 0 | 0.0 | 1 | 0.6 | 4.17E-01 | increasing |
| 24F | 32 | 1.5 | 24 | 3.3 | 58 | 7.6 | **5.02E-03** | **increasing** | **3.72E-04** | **increasing** | 16 | 6.9 | 15 | 9.1 | 4.52E-01 | increasing |
| 27 | 0 | 0.0 | 1 | 0.1 | 2 | 0.3 | 2.58E-01 | increasing | 1.00 |  | 0 | 0.0 | 1 | 0.6 | 4.17E-01 | increasing |
| 29 | 2 | 0.1 | 0 | 0.0 | 0 | 0.0 | 1.00 |  | 1.00 |  | 0 | 0.0 | 0 | 0.0 | 1.00 |  |
| 28A | 2 | 0.1 | 1 | 0.1 | 3 | 0.4 | 1.00 |  | 6.25E-01 | increasing | 0 | 0.0 | 0 | 0.0 | 1.00 |  |
| 28F | 1 | 0.0 | 3 | 0.4 | 6 | 0.8 | 5.50E-02 | increasing | 5.08E-01 | increasing | 1 | 0.4 | 3 | 1.8 | 3.12E-01 | increasing |
| 33A | 6 | 0.3 | 1 | 0.1 | 0 | 0.0 | 6.85E-01 | decreasing | 4.85E-01 | decreasing | 0 | 0.0 | 0 | 0.0 | 1.00 |  |
| 33B | 2 | 0.1 | 1 | 0.1 | 0 | 0.0 | 1.00 |  | 4.85E-01 | decreasing | 0 | 0.0 | 0 | 0.0 | 1.00 |  |
| 34 | 3 | 0.1 | 2 | 0.3 | 7 | 0.9 | 6.08E-01 | increasing | 1.80E-01 | increasing | 0 | 0.0 | 5 | 3.0 | **1.21E-02** | **increasing** |
| 35A | 1 | 0.0 | 1 | 0.1 | 1 | 0.1 | 4.49E-01 | increasing | 1.00 |  | 0 | 0.0 | 0 | 0.0 | 1.00 |  |
| 35B | 2 | 0.1 | 4 | 0.6 | 13 | 1.7 | **4.16E-02** | **increasing** | **4.94E-02** | **increasing** | 5 | 2.2 | 3 | 1.8 | 1.00 |  |
| 35C | 1 | 0.0 | 0 | 0.0 | 2 | 0.3 | 1.00 |  | 5.00E-01 | increasing | 0 | 0.0 | 2 | 1.2 | 1.73E-01 | increasing |
| 35F | 5 | 0.2 | 5 | 0.7 | 8 | 1.0 | 1.38E-01 | increasing | 5.82E-01 | increasing | 1 | 0.4 | 4 | 2.4 | 1.65E-01 | increasing |
| 36 | 1 | 0.0 | 0 | 0.0 | 0 | 0.0 | 1.00 |  | 1.00 |  | 0 | 0.0 | 0 | 0.0 | 1.00 |  |
| 37 | 2 | 0.1 | 1 | 0.1 | 7 | 0.9 | 1.00 |  | 7.05E-02 | increasing | 3 | 1.3 | 2 | 1.2 | 1.00 |  |
| 38 | 12 | 0.6 | 15 | 2.1 | 38 | 5.0 | **1.20E-03** | **increasing** | **3.02E-03** | **increasing** | 10 | 4.3 | 8 | 4.8 | 8.11E-01 | increasing |
| 39 | 0 | 0.0 | 1 | 0.1 | 0 | 0.0 | 2.58E-01 | increasing | 4.85E-01 | decreasing | 0 | 0.0 | 0 | 0.0 | 1.00 |  |
| NT | 4 | 0.2 | 4 | 0.6 | 1 | 0.1 | 2.16E-01 | increasing | 2.05E-01 | decreasing | 0 | 0.0 | 0 | 0.0 | 1.00 |  |
| nonPCV13 | 324 | 15.6 | 214 | 29.7 | 452 | 59.2 | **1.50E-15** | **increasing** | **1.96E-30** | **increasing** | 98 | 42.4 | 127 | 77.0 | **6.34E-12** | **increasing** |
| total | 2075 | 100.0 | 720 | 100.0 | 764 | 100.0 |  |  |  |  | 231 | 100.0 | 165 | 100.0 |  |  |

**Table B: Serotype distribution among isolates from IPD in children (<2 years of age) in Germany (n=2,030).**

| **Serotype** | **Pre-Vaccination 1997-2006** | | **Early Vaccination 2007-2010** | | **Late Vaccination 2010-2014** | | **Pre-Vaccination vs. Early Vaccination** | | **Early Vaccination vs. Late Vaccination** | | **2010-2011** | | **2013-2014** | | **2010-2011 vs. 2013-2014** | |
| --- | --- | --- | --- | --- | --- | --- | --- | --- | --- | --- | --- | --- | --- | --- | --- | --- |
|  | **n** | **%** | **n** | **%** | **n** | **%** | **p-value** | **direction** | **p-value** | **direction** | **n** | **%** | **n** | **%** | **p-value** | **direction** |
| 4 | 45 | 3.8 | 3 | 0.9 | 0 | 0.0 | **4.34E-03** | **decreasing** | 1.18E-01 | decreasing | 0 | 0.0 | 0 | 0.0 | 1.00 |  |
| 6B | 105 | 8.8 | 15 | 4.4 | 5 | 1.4 | **8.05E-03** | **decreasing** | **2.24E-02** | **decreasing** | 2 | 2.1 | 0 | 0.0 | 5.01E-01 | decreasing |
| 9V | 41 | 3.5 | 2 | 0.6 | 0 | 0.0 | **2.53E-03** | **decreasing** | 2.40E-01 | decreasing | 0 | 0.0 | 0 | 0.0 | 1.00 |  |
| 14 | 356 | 30.0 | 20 | 5.9 | 2 | 0.6 | **1.69E-23** | **decreasing** | **3.97E-05** | **decreasing** | 0 | 0.0 | 0 | 0.0 | 1.00 |  |
| 18C | 51 | 4.3 | 8 | 2.4 | 1 | 0.3 | 1.12E-01 | decreasing | **1.87E-02** | **decreasing** | 1 | 1.0 | 0 | 0.0 | 1.00 |  |
| 19F | 103 | 8.7 | 18 | 5.3 | 9 | 2.6 | 5.18E-02 | decreasing | 7.68E-02 | decreasing | 4 | 4.1 | 2 | 2.5 | 6.90E-01 | decreasing |
| 23F | 87 | 7.3 | 3 | 0.9 | 0 | 0.0 | **5.83E-07** | **decreasing** | 1.18E-01 | decreasing | 0 | 0.0 | 0 | 0.0 | 1.00 |  |
| **PCV7** | **788** | **66.3** | **69** | **20.4** | **17** | **4.8** | **3.46E-52** | **decreasing** | **3.20E-10** | **decreasing** | **7** | **7.2** | **2** | **2.5** | 1.85E-01 | decreasing |
| 1 | 30 | 2.5 | 27 | 8.0 | 11 | 3.1 | **2.39E-05** | **increasing** | **6.91E-03** | **decreasing** | 6 | 6.2 | 1 | 1.2 | 1.28E-01 | decreasing |
| 5 | 7 | 0.6 | 1 | 0.3 | 1 | 0.3 | 1.00 |  | 1.00 | decreasing | 1 | 1.0 | 0 | 0.0 | 1.00 |  |
| 7F | 75 | 6.3 | 53 | 15.7 | 20 | 5.7 | **3.19E-07** | **increasing** | **2.03E-05** | **decreasing** | 9 | 9.3 | 1 | 1.2 | **2.29E-02** | **decreasing** |
| PCV10 | 900 | 75.8 | 150 | 44.4 | 49 | 14.0 | **2.07E-26** | **decreasing** | **5.49E-19** | **decreasing** | 23 | 23.7 | 4 | 4.9 | **5.77E-04** | **decreasing** |
| PCV10non7 | 112 | 9.4 | 81 | 24.0 | 32 | 9.1 | **3.02E-11** | **increasing** | **1.17E-07** | **decreasing** | 16 | 16.5 | 2 | 2.5 | **2.06E-03** | **decreasing** |
| 3 | 30 | 2.5 | 21 | 6.2 | 17 | 4.8 | **1.77E-03** | **increasing** | 5.05E-01 | decreasing | 5 | 5.2 | 4 | 4.9 | 1.00 |  |
| 6A | 47 | 4.0 | 16 | 4.7 | 5 | 1.4 | 5.36E-01 | increasing | **1.38E-02** | **decreasing** | 1 | 1.0 | 0 | 0.0 | 1.00 |  |
| 19A | 45 | 3.8 | 42 | 12.4 | 42 | 12.0 | **3.03E-08** | **increasing** | 9.07E-01 | decreasing | 22 | 22.7 | 4 | 4.9 | **1.06E-03** | **decreasing** |
| PCV13 | 1022 | 86.0 | 229 | 67.8 | 113 | 32.2 | **3.34E-13** | **decreasing** | **6.01E-21** | **decreasing** | 51 | 52.6 | 12 | 14.8 | **1.29E-07** | **decreasing** |
| PCV13non7 | 234 | 19.7 | 160 | 47.3 | 96 | 27.4 | **9.90E-23** | **increasing** | **7.25E-08** | **decreasing** | 44 | 45.4 | 10 | 12.3 | **1.45E-06** | **decreasing** |
| PCV13non10 | 122 | 10.3 | 79 | 23.4 | 64 | 18.2 | **3.00E-09** | **increasing** | 1.10E-01 | decreasing | 28 | 28.9 | 8 | 9.9 | **2.40E-03** | **decreasing** |
| 2 | 0 | 0.0 | 0 | 0.0 | 0 | 0.0 | 1.00 |  | 1.00 |  | 0 | 0.0 | 0 | 0.0 | 1.00 |  |
| 8 | 10 | 0.8 | 3 | 0.9 | 5 | 1.4 | 1.00 |  | 7.25E-01 | increasing | 1 | 1.0 | 2 | 2.5 | 5.92E-01 | increasing |
| 9N | 10 | 0.8 | 2 | 0.6 | 7 | 2.0 | 1.00 |  | 1.78E-01 | increasing | 2 | 2.1 | 1 | 1.2 | 1.00 |  |
| 10A | 20 | 1.7 | 21 | 6.2 | 30 | 8.5 | **3.29E-05** | **increasing** | 2.49E-01 | increasing | 4 | 4.1 | 7 | 8.6 | 2.31E-01 | increasing |
| 11A | 2 | 0.2 | 3 | 0.9 | 4 | 1.1 | 7.54E-02 | increasing | 1.00 |  | 0 | 0.0 | 1 | 1.2 | 4.55E-01 | increasing |
| 12F | 4 | 0.3 | 4 | 1.2 | 14 | 4.0 | 7.76E-02 | increasing | **2.93E-02** | **increasing** | 1 | 1.0 | 5 | 6.2 | 9.36E-02 | increasing |
| 15B | 5 | 0.4 | 10 | 3.0 | 16 | 4.6 | **2.58E-04** | **increasing** | 3.20E-01 | increasing | 3 | 3.1 | 5 | 6.2 | 4.71E-01 | increasing |
| 17F | 2 | 0.2 | 3 | 0.9 | 1 | 0.3 | 7.54E-02 | increasing | 3.65E-01 | decreasing | 0 | 0.0 | 1 | 1.2 | 4.55E-01 | increasing |
| 20 | 2 | 0.2 | 1 | 0.3 | 0 | 0.0 | 5.28E-01 | increasing | 4.91E-01 | decreasing | 0 | 0.0 | 0 | 0.0 | 1.00 |  |
| 22F | 7 | 0.6 | 6 | 1.8 | 13 | 3.7 | 4.69E-02 | increasing | 1.63E-01 | increasing | 2 | 2.1 | 3 | 3.7 | 6.60E-01 | increasing |
| 33F | 7 | 0.6 | 6 | 1.8 | 12 | 3.4 | 4.69E-02 | increasing | 2.33E-01 | increasing | 3 | 3.1 | 3 | 3.7 | 1.00 |  |
| PPV23 | 1044 | 87.9 | 272 | 80.5 | 210 | 59.8 | **8.75E-04** | **decreasing** | **3.00E-09** | **decreasing** | 66 | 68.0 | 40 | 49.4 | **1.42E-02** | **decreasing** |
| 6C | 5 | 0.4 | 2 | 0.6 | 3 | 0.9 | 6.54E-01 | increasing | 1.00 |  | 1 | 1.0 | 1 | 1.2 | 1.00 |  |
| 9A | 11 | 0.9 | 0 | 0.0 | 0 | 0.0 | 1.36E-01 | decreasing | 1.00 |  | 0 | 0.0 | 0 | 0.0 | 1.00 |  |
| 9L | 2 | 0.2 | 0 | 0.0 | 0 | 0.0 | 1.00 |  | 1.00 |  | 0 | 0.0 | 0 | 0.0 | 1.00 |  |
| 12A | 2 | 0.2 | 0 | 0.0 | 2 | 0.6 | 1.00 |  | 4.99E-01 | increasing | 0 | 0.0 | 2 | 2.5 | 2.06E-01 | increasing |
| 12B | 1 | 0.1 | 0 | 0.0 | 0 | 0.0 | 1.00 |  | 1.00 |  | 0 | 0.0 | 0 | 0.0 | 1.00 |  |
| 13 | 2 | 0.2 | 1 | 0.3 | 0 | 0.0 | 5.28E-01 | increasing | 4.91E-01 | decreasing | 0 | 0.0 | 0 | 0.0 | 1.00 |  |
| 15A | 7 | 0.6 | 1 | 0.3 | 3 | 0.9 | 1.00 |  | 6.24E-01 | increasing | 1 | 1.0 | 0 | 0.0 | 1.00 |  |
| 15C | 6 | 0.5 | 8 | 2.4 | 20 | 5.7 | **4.54E-03** | **increasing** | **3.29E-02** | **increasing** | 6 | 6.2 | 6 | 7.4 | 7.72E-01 | increasing |
| 15F | 1 | 0.1 | 0 | 0.0 | 0 | 0.0 | 1.00 |  | 1.00 |  | 0 | 0.0 | 0 | 0.0 | 1.00 |  |
| 16F | 2 | 0.2 | 1 | 0.3 | 4 | 1.1 | 5.28E-01 | increasing | 3.74E-01 | increasing | 2 | 2.1 | 1 | 1.2 | 1.00 |  |
| 18A | 4 | 0.3 | 2 | 0.6 | 0 | 0.0 | 6.19E-01 | increasing | 2.40E-01 | decreasing | 0 | 0.0 | 0 | 0.0 | 1.00 |  |
| 18B | 4 | 0.3 | 0 | 0.0 | 0 | 0.0 | 5.81E-01 | decreasing | 1.00 |  | 0 | 0.0 | 0 | 0.0 | 1.00 |  |
| 18F | 2 | 0.2 | 0 | 0.0 | 0 | 0.0 | 1.00 |  | 1.00 |  | 0 | 0.0 | 0 | 0.0 | 1.00 |  |
| 19C | 1 | 0.1 | 0 | 0.0 | 0 | 0.0 | 1.00 |  | 1.00 |  | 0 | 0.0 | 0 | 0.0 | 1.00 |  |
| 21 | 0 | 0.0 | 1 | 0.3 | 5 | 1.4 | 2.21E-01 | increasing | 2.17E-01 | increasing | 0 | 0.0 | 0 | 0.0 | 1.00 |  |
| 23A | 3 | 0.3 | 2 | 0.6 | 6 | 1.7 | 3.07E-01 | increasing | 2.87E-01 | increasing | 1 | 1.0 | 3 | 3.7 | 3.32E-01 | increasing |
| 23B | 3 | 0.3 | 0 | 0.0 | 15 | 4.3 | 1.00 |  | **5.43E-05** | **increasing** | 5 | 5.2 | 0 | 0.0 | 6.40E-02 | decreasing |
| 24A | 1 | 0.1 | 0 | 0.0 | 0 | 0.0 | 1.00 |  | 1.00 |  | 0 | 0.0 | 0 | 0.0 | 1.00 |  |
| 24B | 0 | 0.0 | 1 | 0.3 | 0 | 0.0 | 2.21E-01 | increasing | 4.91E-01 | decreasing | 0 | 0.0 | 0 | 0.0 | 1.00 | increasing |
| 24F | 20 | 1.7 | 12 | 3.6 | 35 | 10.0 | **4.98E-02** | **increasing** | **8.02E-04** | **increasing** | 8 | 8.2 | 12 | 14.8 | 2.33E-01 | increasing |
| 27 | 0 | 0.0 | 1 | 0.3 | 2 | 0.6 | 2.21E-01 | increasing | 1.00 |  | 0 | 0.0 | 1 | 1.2 | 4.55E-01 | increasing |
| 29 | 1 | 0.1 | 0 | 0.0 | 0 | 0.0 | 1.00 |  | 1.00 |  | 0 | 0.0 | 0 | 0.0 | 1.00 |  |
| 28A | 1 | 0.1 | 0 | 0.0 | 1 | 0.3 | 1.00 |  | 1.00 |  | 0 | 0.0 | 0 | 0.0 | 1.00 |  |
| 28F | 1 | 0.1 | 2 | 0.6 | 4 | 1.1 | 1.25E-01 | increasing | 6.86E-01 | increasing | 0 | 0.0 | 3 | 3.7 | 9.23E-02 | increasing |
| 31 | 0 | 0.0 | 0 | 0.0 | 1 | 0.3 | 1.00 |  | 1.00 |  | 0 | 0.0 | 1 | 1.2 | 4.55E-01 | increasing |
| 33A | 3 | 0.3 | 1 | 0.3 | 0 | 0.0 | 1.00 |  | 4.91E-01 | decreasing | 0 | 0.0 | 0 | 0.0 | 1.00 |  |
| 33B | 1 | 0.1 | 0 | 0.0 | 0 | 0.0 | 1.00 |  | 1.00 |  | 0 | 0.0 | 0 | 0.0 | 1.00 |  |
| 34 | 2 | 0.2 | 0 | 0.0 | 1 | 0.3 | 1.00 |  | 1.00 |  | 0 | 0.0 | 1 | 1.2 | 4.55E-01 | increasing |
| 35A | 1 | 0.1 | 0 | 0.0 | 1 | 0.3 | 1.00 |  | 1.00 |  | 0 | 0.0 | 0 | 0.0 | 1.00 |  |
| 35B | 0 | 0.0 | 0 | 0.0 | 5 | 1.4 | 1.00 |  | 6.18E-02 | increasing | 0 | 0.0 | 3 | 3.7 | 9.23E-02 | increasing |
| 35F | 0 | 0.0 | 4 | 1.2 | 3 | 0.9 | **2.37E-03** | **increasing** | 7.20E-01 | decreasing | 0 | 0.0 | 2 | 2.5 | 2.06E-01 | increasing |
| 37 | 1 | 0.1 | 0 | 0.0 | 1 | 0.3 | 1.00 |  | 1.00 |  | 1 | 1.0 | 0 | 0.0 | 1.00 |  |
| 38 | 7 | 0.6 | 8 | 2.4 | 23 | 6.6 | **7.87E-03** | **increasing** | **9.41E-03** | **increasing** | 5 | 5.2 | 5 | 6.2 | 1.00 |  |
| NT | 2 | 0.2 | 3 | 0.9 | 1 | 0.3 | 7.54E-02 | increasing | 3.65E-01 | decreasing | 0 | 0.0 | 0 | 0.0 | 1.00 |  |
| nonPCV13 | 166 | 14.0 | 109 | 32.2 | 238 | 67.8 | **3.34E-13** | **increasing** | **6.01E-21** | **increasing** | 46 | 47.4 | 69 | 85.2 | **1.29E-07** | **increasing** |
| total | 1188 | 100.0 | 338 | 100.0 | 351 | 100.0 |  |  |  |  | 97 | 100.0 | 81 | 100.0 |  |  |

**Table C: Serotype distribution among isolates from IPD in children (2-4 years of age) in Germany (n=1,007)**.

| **Serotype** | **Pre-Vaccination 1997-2006** | | **Early Vaccination 2007-2010** | | **Late Vaccination 2010-2014** | | **Pre-Vaccination vs. Early Vaccination** | | **Early Vaccination vs. Late Vaccination** | | **2010-2011** | | **2013-2014** | | **2010-2011 vs. 2013-2014** | |
| --- | --- | --- | --- | --- | --- | --- | --- | --- | --- | --- | --- | --- | --- | --- | --- | --- |
|  | **n** | **%** | **n** | **%** | **n** | **%** | **p-value** | **direction** | **p-value** | **direction** | **n** | **%** | **n** | **%** | **p-value** | **direction** |
| 4 | 19 | 3.7 | 1 | 0.5 | 0 | 0.0 | **2.06E-02** | **decreasing** | 4.86E-01 | decreasing | 0 | 0.0 | 0 | 0.0 | 1.00 |  |
| 6B | 43 | 8.3 | 3 | 1.5 | 1 | 0.5 | **2.83E-04** | **decreasing** | 3.59E-01 | decreasing | 0 | 0.0 | 1 | 2.2 | 4.04E-01 | increase |
| 9V | 21 | 4.1 | 5 | 2.5 | 0 | 0.0 | 3.78E-01 | decreasing | **2.64E-02** | **decreasing** | 0 | 0.0 | 0 | 0.0 | 1.00 |  |
| 14 | 162 | 31.5 | 17 | 8.3 | 2 | 0.9 | **5.15E-12** | **decreasing** | **2.21E-04** | **decreasing** | 0 | 0.0 | 1 | 2.2 | 4.04E-01 | increase |
| 18C | 37 | 7.2 | 16 | 7.8 | 2 | 0.9 | 7.53E-01 | increasing | **4.25E-04** | **decreasing** | 2 | 2.9 | 0 | 0.0 | 5.14E-01 | decrease |
| 19F | 33 | 6.4 | 10 | 4.9 | 4 | 1.9 | 4.91E-01 | decreasing | 1.04E-01 | decreasing | 2 | 2.9 | 2 | 4.3 | 1.00 |  |
| 23F | 46 | 8.9 | 5 | 2.5 | 2 | 0.9 | **1.85E-03** | **decreasing** | 2.73E-01 | decreasing | 0 | 0.0 | 0 | 0.0 | 1.00 |  |
| PCV7 | 361 | 70.1 | 57 | 27.9 | 11 | 5.1 | **5.30E-25** | **decreasing** | **9.64E-11** | **decreasing** | 4 | 5.9 | 4 | 8.7 | 7.12E-01 | increase |
| 1 | 31 | 6.0 | 32 | 15.7 | 20 | 9.3 | **1.03E-04** | **increasing** | 5.39E-02 | decreasing | 11 | 16.2 | 0 | 0.0 | **2.83E-03** | **decrease** |
| 5 | 4 | 0.8 | 0 | 0.0 | 0 | 0.0 | 5.82E-01 | decreasing | 1.00 |  | 0 | 0.0 | 0 | 0.0 | 1.00 |  |
| 7F | 25 | 4.9 | 24 | 11.8 | 18 | 8.3 | **1.61E-03** | **increasing** | 2.58E-01 | decreasing | 9 | 13.2 | 0 | 0.0 | 1.05E-02 | decrease |
| PCV10 | 421 | 81.7 | 113 | 55.4 | 49 | 22.7 | **1.90E-12** | **decreasing** | **5.54E-12** | **decreasing** | 24 | 35.3 | 4 | 8.7 | **1.58E-03** | **decrease** |
| PCV10non7 | 60 | 11.7 | 56 | 27.5 | 38 | 17.6 | **8.72E-07** | **increasing** | **1.90E-02** | **decreasing** | 20 | 29.4 | 0 | 0.0 | **9.24E-06** | **decrease** |
| 3 | 8 | 1.6 | 12 | 5.9 | 13 | 6.0 | **3.85E-03** | **increasing** | 1.00 |  | 2 | 2.9 | 5 | 10.9 | 1.16E-01 | increase |
| 6A | 12 | 2.3 | 7 | 3.4 | 1 | 0.5 | 4.41E-01 | increasing | **3.28E-02** | **decreasing** | 0 | 0.0 | 0 | 0.0 | 1.00 |  |
| 19A | 7 | 1.4 | 8 | 3.9 | 25 | 11.6 | **4.13E-02** | **increasing** | **3.59E-03** | **increasing** | 10 | 14.7 | 3 | 6.5 | 2.36E-01 | decrease |
| PCV13 | 448 | 87.0 | 140 | 68.6 | 88 | 40.7 | **4.86E-08** | **decreasing** | **1.10E-08** | **decreasing** | 36 | 52.9 | 12 | 26.1 | **6.52E-03** | **decrease** |
| PCV13non7 | 87 | 16.9 | 83 | 40.7 | 77 | 35.6 | **6.36E-11** | **increasing** | 3.15E-01 | decreasing | 32 | 47.1 | 8 | 17.4 | **1.31E-03** | **decrease** |
| PCV13non10 | 27 | 5.2 | 27 | 13.2 | 39 | 18.1 | **4.76E-04** | **increasing** | 1.83E-01 | increasing | 12 | 17.6 | 8 | 17.4 | 1.00 |  |
| 2 | 0 | 0.0 | 0 | 0.0 | 0 | 0.0 | 1.00 |  | 1.00 |  | 0 | 0.0 | 0 | 0.0 | 1.00 |  |
| 8 | 1 | 0.2 | 2 | 1.0 | 2 | 0.9 | 1.95E-01 | increasing | 1.00 |  | 0 | 0.0 | 2 | 4.3 | 1.61E-01 | increase |
| 9N | 4 | 0.8 | 4 | 2.0 | 1 | 0.5 | 2.33E-01 | increasing | 2.04E-01 | decreasing | 0 | 0.0 | 1 | 2.2 | 4.04E-01 | increase |
| 10A | 4 | 0.8 | 5 | 2.5 | 9 | 4.2 | 1.27E-01 | increasing | 4.19E-01 | increasing | 2 | 2.9 | 3 | 6.5 | 3.91E-01 | increase |
| 11A | 0 | 0.0 | 0 | 0.0 | 7 | 3.2 | 1.00E+00 |  | **1.51E-02** | **increasing** | 2 | 2.9 | 1 | 2.2 | 1.00 |  |
| 12F | 2 | 0.4 | 3 | 1.5 | 9 | 4.2 | 1.41E-01 | increasing | 1.42E-01 | increasing | 2 | 2.9 | 2 | 4.3 | 1.00 |  |
| 15B | 8 | 1.6 | 5 | 2.5 | 6 | 2.8 | 5.34E-01 | increasing | 1.00 |  | 0 | 0.0 | 1 | 2.2 | 4.04E-01 | increase |
| 17F | 0 | 0.0 | 0 | 0.0 | 1 | 0.5 | 1.00 |  | 1.00 |  | 0 | 0.0 | 1 | 2.2 | 4.04E-01 | increase |
| 20 | 0 | 0.0 | 0 | 0.0 | 0 | 0.0 | 1.00 |  | 1.00 |  | 0 | 0.0 | 0 | 0.0 | 1.00 |  |
| 22F | 5 | 1.0 | 4 | 2.0 | 7 | 3.2 | 2.82E-01 | increasing | 5.45E-01 | increasing | 1 | 1.5 | 0 | 0.0 | 1.00 |  |
| 33F | 1 | 0.2 | 1 | 0.5 | 6 | 2.8 | 4.87E-01 | increasing | 1.23E-01 | increasing | 1 | 1.5 | 2 | 4.3 | 5.64E-01 | increase |
| PPV23 | 461 | 89.5 | 157 | 77.0 | 135 | 62.5 | **2.64E-05** | **decreasing** | **1.45E-03** | **decreasing** | 44 | 64.7 | 25 | 54.3 | 3.30E-01 | decrease |
| 6C | 1 | 0.2 | 1 | 0.5 | 3 | 1.4 | 4.87E-01 | increasing | 6.24E-01 | increasing | 0 | 0.0 | 0 | 0.0 | 1.00 |  |
| 9A | 3 | 0.6 | 0 | 0.0 | 0 | 0.0 | 5.62E-01 | decreasing | 1.00 |  | 0 | 0.0 | 0 | 0.0 | 1.00 |  |
| 9L | 1 | 0.2 | 0 | 0.0 | 1 | 0.5 | 1.00 |  | 1.00 |  | 0 | 0.0 | 0 | 0.0 | 1.00 |  |
| 10B | 0 | 0.0 | 0 | 0.0 | 1 | 0.5 | 1.00 |  | 1.00 |  | 1 | 1.5 | 0 | 0.0 | 1.00 |  |
| 12B | 1 | 0.2 | 1 | 0.5 | 0 | 0.0 | 4.87E-01 | increasing | 4.86E-01 | decreasing | 0 | 0.0 | 0 | 0.0 | 1.00 |  |
| 15A | 2 | 0.4 | 1 | 0.5 | 4 | 1.9 | 1.00 |  | 3.73E-01 | increasing | 0 | 0.0 | 1 | 2.2 | 4.04E-01 | increase |
| 15C | 6 | 1.2 | 8 | 3.9 | 14 | 6.5 | **3.04E-02** | **increasing** | 2.78E-01 | increasing | 5 | 7.4 | 5 | 10.9 | 5.21E-01 | increase |
| 16F | 2 | 0.4 | 3 | 1.5 | 1 | 0.5 | 1.41E-01 | increasing | 3.59E-01 | decreasing | 0 | 0.0 | 1 | 2.2 | 4.04E-01 | increase |
| 18A | 1 | 0.2 | 0 | 0.0 | 0 | 0.0 | 1.00 |  | 1.00 |  | 0 | 0.0 | 0 | 0.0 | 1.00 |  |
| 18B | 9 | 1.7 | 0 | 0.0 | 0 | 0.0 | 6.71E-02 | decreasing | 1.00 |  | 0 | 0.0 | 0 | 0.0 | 1.00 |  |
| 18F | 2 | 0.4 | 1 | 0.5 | 0 | 0.0 | 1.00 |  | 4.86E-01 | decreasing | 0 | 0.0 | 0 | 0.0 | 1.00 |  |
| 19B | 1 | 0.2 | 0 | 0.0 | 0 | 0.0 | 1.00 |  | 1.00 |  | 0 | 0.0 | 0 | 0.0 | 1.00 |  |
| 19C | 1 | 0.2 | 0 | 0.0 | 0 | 0.0 | 1.00 |  | 1.00 |  | 0 | 0.0 | 0 | 0.0 | 1.00 |  |
| 21 | 0 | 0.0 | 1 | 0.5 | 2 | 0.9 | 2.84E-01 | increasing | 1.00 |  | 2 | 2.9 | 0 | 0.0 | 5.14E-01 | decrease |
| 23A | 0 | 0.0 | 1 | 0.5 | 2 | 0.9 | 2.84E-01 | increasing | 1.00 |  | 1 | 1.5 | 0 | 0.0 | 1.00 |  |
| 23B | 1 | 0.2 | 3 | 1.5 | 12 | 5.6 | 7.14E-02 | increasing | **3.31E-02** | **increasing** | 3 | 4.4 | 4 | 8.7 | 4.37E-01 | increase |
| 24B | 0 | 0.0 | 0 | 0.0 | 1 | 0.5 | 1.00 |  | 1.00 |  | 0 | 0.0 | 1 | 2.2 | 4.04E-01 | increase |
| 24F | 3 | 0.6 | 9 | 4.4 | 15 | 6.9 | **9.85E-04** | **increasing** | 2.98E-01 | increasing | 7 | 10.3 | 1 | 2.2 | 1.40E-01 | decrease |
| 28A | 0 | 0.0 | 0 | 0.0 | 1 | 0.5 | 1.00 |  | 1.00 |  | 0 | 0.0 | 0 | 0.0 | 1.00 |  |
| 33A | 2 | 0.4 | 0 | 0.0 | 0 | 0.0 | 1.00 |  | 1.00 |  | 0 | 0.0 | 0 | 0.0 | 1.00 |  |
| 33B | 0 | 0.0 | 1 | 0.5 | 0 | 0.0 | 2.84E-01 | increasing | 4.86E-01 | decreasing | 0 | 0.0 | 0 | 0.0 | 1.00 |  |
| 34 | 0 | 0.0 | 1 | 0.5 | 2 | 0.9 | 2.84E-01 | increasing | 1.00 |  | 0 | 0.0 | 2 | 4.3 | 1.61E-01 | increase |
| 35B | 1 | 0.2 | 1 | 0.5 | 2 | 0.9 | 4.87E-01 | increasing | 1.00 |  | 1 | 1.5 | 0 | 0.0 | 1.00 |  |
| 35F | 0 | 0.0 | 0 | 0.0 | 3 | 1.4 | 1.00 |  | 2.49E-01 | increasing | 0 | 0.0 | 2 | 4.3 | 1.61E-01 | increase |
| 37 | 1 | 0.2 | 0 | 0.0 | 2 | 0.9 | 1.00 |  | 4.99E-01 | increasing | 0 | 0.0 | 1 | 2.2 | 4.04E-01 | increase |
| 38 | 3 | 0.6 | 7 | 3.4 | 14 | 6.5 | **7.20E-03** | **increasing** | 1.82E-01 | increasing | 4 | 5.9 | 3 | 6.5 | 1.00 |  |
| 39 | 0 | 0.0 | 1 | 0.5 | 0 | 0.0 | 2.84E-01 | increasing | 4.86E-01 | decreasing | 0 | 0.0 | 0 | 0.0 | 1.00 |  |
| NT | 1 | 0.2 | 0 | 0.0 | 0 | 0.0 | 1.00 |  | 1.00 |  | 0 | 0.0 | 0 | 0.0 | 1.00 |  |
| nonPCV13 | 67 | 13.0 | 64 | 31.4 | 128 | 59.3 | **4.86E-08** | **increasing** | **1.10E-08** | **increasing** | 32 | 47.1 | 34 | 73.9 | **6.52E-03** | **increase** |
| total | 515 | 100.0 | 204 | 100.0 | 216 | 100.0 |  |  |  |  | 68 | 100.0 | 46 | 100.0 |  |  |

**Table D: Serotype distribution among isolates from IPD in children (5-15 years of age) in Germany (n=816)**.

| **Serotype** | **Pre-Vaccination 1997-2006** | | **Early Vaccination 2007-2010** | | **Late Vaccination 2010-2014** | | **Pre-Vaccination vs. Early Vaccination** | | **Early Vaccination vs. Late Vaccination** | | **2010-2011** | | **2013-2014** | | **2010-2011 vs. 2013-2014** | |
| --- | --- | --- | --- | --- | --- | --- | --- | --- | --- | --- | --- | --- | --- | --- | --- | --- |
|  | **n** | **%** | **n** | **%** | **n** | **%** | **p-value** | **direction** | **p-value** | **direction** | **n** | **%** | **n** | **%** | **p-value** | **direction** |
| 4 | 14 | 3.8 | 3 | 1.7 | 0 | 0.0 | 2.92E-01 | decreasing | 1.06E-01 | decreasing | 0 | 0.0 | 0 | 0.0 | 1.00 |  |
| 6B | 8 | 2.2 | 7 | 3.9 | 1 | 0.5 | 2.66E-01 | increasing | **2.96E-02** | decreasing | 1 | 1.5 | 0 | 0.0 | 1.00 |  |
| 9V | 19 | 5.1 | 5 | 2.8 | 1 | 0.5 | 2.69E-01 | decreasing | 1.06E-01 | decreasing | 1 | 1.5 | 0 | 0.0 | 1.00 |  |
| 14 | 30 | 8.1 | 4 | 2.2 | 2 | 1.0 | **7.37E-03** | decreasing | 4.29E-01 | decreasing | 1 | 1.5 | 0 | 0.0 | 1.00 |  |
| 18C | 32 | 8.6 | 13 | 7.3 | 2 | 1.0 | 7.40E-01 | decreasing | **2.48E-03** | decreasing | 1 | 1.5 | 1 | 2.6 | 1.00 |  |
| 19F | 13 | 3.5 | 8 | 4.5 | 5 | 2.5 | 6.36E-01 | increasing | 3.99E-01 | decreasing | 2 | 3.0 | 1 | 2.6 | 1.00 |  |
| 23F | 17 | 4.6 | 3 | 1.7 | 1 | 0.5 | 1.41E-01 | decreasing | 3.49E-01 | decreasing | 0 | 0.0 | 0 | 0.0 | 1.00 |  |
| PCV7 | 133 | 35.8 | 43 | 24.2 | 12 | 6.1 | **6.31E-03** | decreasing | **7.52E-07** | decreasing | 6 | 9.1 | 2 | 5.3 | 7.07E-01 | decrease |
| 1 | 90 | 24.2 | 55 | 30.9 | 46 | 23.4 | 9.89E-02 | increasing | 1.04E-01 | decreasing | 22 | 33.3 | 5 | 13.2 | **3.54E-02** | decrease |
| 5 | 1 | 0.3 | 3 | 1.7 | 0 | 0.0 | 1.02E-01 | increasing | 1.06E-01 | decreasing | 0 | 0.0 | 0 | 0.0 | 1.00 |  |
| 7F | 19 | 5.1 | 19 | 10.7 | 27 | 13.7 | **1.96E-02** | increasing | 4.32E-01 | increasing | 10 | 15.2 | 4 | 10.5 | 5.67E-01 | decrease |
| PCV10 | 243 | 65.3 | 120 | 67.4 | 85 | 43.1 | 7.00E-01 | increasing | **2.75E-06** | decreasing | 38 | 57.6 | 11 | 28.9 | **7.64E-03** | decrease |
| PCV10non7 | 110 | 29.6 | 77 | 43.3 | 73 | 37.1 | **2.03E-03** | increasing | 2.46E-01 | decreasing | 32 | 48.5 | 9 | 23.7 | **1.37E-02** | decrease |
| 3 | 19 | 5.1 | 8 | 4.5 | 16 | 8.1 | 8.36E-01 | decreasing | 2.05E-01 | increasing | 5 | 7.6 | 3 | 7.9 | 1.00 |  |
| 6A | 13 | 3.5 | 5 | 2.8 | 0 | 0.0 | 8.01E-01 | decreasing | **2.34E-02** | decreasing | 0 | 0.0 | 0 | 0.0 | 1.00 |  |
| 19A | 6 | 1.6 | 4 | 2.2 | 10 | 5.1 | 7.34E-01 | increasing | 1.79E-01 | increasing | 3 | 4.5 | 0 | 0.0 | 2.98E-01 | decrease |
| PCV13 | 281 | 75.5 | 137 | 77.0 | 111 | 56.3 | 7.50E-01 | increasing | **3.03E-05** | decreasing | 46 | 69.7 | 14 | 36.8 | **1.82E-03** | decrease |
| PCV13non7 | 148 | 39.8 | 94 | 52.8 | 99 | 50.3 | **4.44E-03** | increasing | 6.79E-01 | decreasing | 40 | 60.6 | 12 | 31.6 | **7.71E-03** | decrease |
| PCV13non10 | 38 | 10.2 | 17 | 9.6 | 26 | 13.2 | 8.80E-01 | decreasing | 3.31E-01 | increasing | 8 | 12.1 | 3 | 7.9 | 7.42E-01 | decrease |
| 2 | 0 | 0.0 | 0 | 0.0 | 0 | 0.0 | 1.00 | increasing | 1.00 |  | 0 | 0.0 | 0 | 0.0 | 1.00 |  |
| 8 | 5 | 1.3 | 1 | 0.6 | 2 | 1.0 | 6.69E-01 | decreasing | 1.00 |  | 0 | 0.0 | 0 | 0.0 | 1.00 |  |
| 9N | 5 | 1.3 | 1 | 0.6 | 0 | 0.0 | 6.69E-01 | decreasing | 4.75E-01 | decreasing | 0 | 0.0 | 0 | 0.0 | 1.00 |  |
| 10A | 8 | 2.2 | 3 | 1.7 | 3 | 1.5 | 1.00 |  | 1.00 |  | 2 | 3.0 | 1 | 2.6 | 1.00 |  |
| 11A | 5 | 1.3 | 5 | 2.8 | 7 | 3.6 | 3.05E-01 | increasing | 7.74E-01 | increasing | 1 | 1.5 | 3 | 7.9 | 1.37E-01 | increase |
| 12F | 2 | 0.5 | 1 | 0.6 | 10 | 5.1 | 1.00 |  | **1.17E-02** | increasing | 2 | 3.0 | 2 | 5.3 | 6.22E-01 | increase |
| 15B | 1 | 0.3 | 1 | 0.6 | 1 | 0.5 | 5.43E-01 | increasing | 1.00 |  | 0 | 0.0 | 1 | 2.6 | 3.65E-01 | increase |
| 17F | 3 | 0.8 | 1 | 0.6 | 0 | 0.0 | 1.00 |  | 4.75E-01 | decreasing | 0 | 0.0 | 0 | 0.0 | 1.00 |  |
| 20 | 2 | 0.5 | 0 | 0.0 | 0 | 0.0 | 1.00 |  | 1.00 |  | 0 | 0.0 | 0 | 0.0 | 1.00 |  |
| 22F | 2 | 0.5 | 5 | 2.8 | 6 | 3.0 | **3.89E-02** | increasing | 1.00 |  | 2 | 3.0 | 2 | 5.3 | 6.22E-01 | increase |
| 33F | 3 | 0.8 | 2 | 1.1 | 5 | 2.5 | 6.61E-01 | increasing | 4.53E-01 | increasing | 0 | 0.0 | 3 | 7.9 | **4.63E-02** | increase |
| PPV23 | 304 | 81.7 | 152 | 85.4 | 145 | 73.6 | 3.33E-01 | increasing | **5.15E-03** | decreasing | 53 | 80.3 | 26 | 68.4 | 2.33E-01 | decrease |
| 6C | 3 | 0.8 | 0 | 0.0 | 5 | 2.5 | 5.55E-01 | decreasing | 6.24E-02 | increasing | 0 | 0.0 | 4 | 10.5 | **1.61E-02** | increase |
| 7A | 0 | 0.0 | 1 | 0.6 | 0 | 0.0 | 3.24E-01 | increasing | 4.75E-01 | decreasing | 0 | 0.0 | 0 | 0.0 | 1.00 |  |
| 9A | 6 | 1.6 | 0 | 0.0 | 0 | 0.0 | 1.84E-01 | decreasing | 1.00 |  | 0 | 0.0 | 0 | 0.0 | 1.00 |  |
| 9L | 1 | 0.3 | 0 | 0.0 | 0 | 0.0 | 1.00 |  | 1.00 |  | 0 | 0.0 | 0 | 0.0 | 1.00 |  |
| 11B | 1 | 0.3 | 1 | 0.6 | 0 | 0.0 | 5.43E-01 | increasing | 4.75E-01 | decreasing | 0 | 0.0 | 0 | 0.0 | 1.00 |  |
| 12A | 1 | 0.3 | 0 | 0.0 | 0 | 0.0 | 1.00 |  | 1.00 |  | 0 | 0.0 | 0 | 0.0 | 1.00 |  |
| 15A | 9 | 2.4 | 2 | 1.1 | 3 | 1.5 | 5.16E-01 | decreasing | 1.00 |  | 1 | 1.5 | 0 | 0.0 | 1.00 |  |
| 15C | 2 | 0.5 | 1 | 0.6 | 1 | 0.5 | 1.00 |  | 1.00 |  | 0 | 0.0 | 0 | 0.0 | 1.00 |  |
| 18A | 2 | 0.5 | 0 | 0.0 | 1 | 0.5 | 1.00 |  | 1.00 |  | 0 | 0.0 | 0 | 0.0 | 1.00 |  |
| 18B | 1 | 0.3 | 0 | 0.0 | 0 | 0.0 | 1.00 |  | 1.00 |  | 0 | 0.0 | 0 | 0.0 | 1.00 |  |
| 21 | 1 | 0.3 | 0 | 0.0 | 2 | 1.0 | 1.00 |  | 5.00E-01 | increasing | 0 | 0.0 | 0 | 0.0 | 1.00 |  |
| 23A | 3 | 0.8 | 3 | 1.7 | 2 | 1.0 | 3.94E-01 | increasing | 6.71E-01 | decreasing | 0 | 0.0 | 1 | 2.6 | 3.65E-01 | increase |
| 23B | 0 | 0.0 | 0 | 0.0 | 7 | 3.6 | 1.00 |  | **1.56E-02** | increasing | 2 | 3.0 | 0 | 0.0 | 5.32E-01 | decrease |
| 24F | 9 | 2.4 | 3 | 1.7 | 8 | 4.1 | 7.60E-01 | decreasing | 2.26E-01 | increasing | 1 | 1.5 | 2 | 5.3 | 5.52E-01 | increase |
| 28A | 1 | 0.3 | 1 | 0.6 | 1 | 0.5 | 5.43E-01 | increasing | 1.00 |  | 0 | 0.0 | 0 | 0.0 | 1.00 |  |
| 28F | 0 | 0.0 | 1 | 0.6 | 2 | 1.0 | 3.24E-01 | increasing | 1.00 |  | 1 | 1.5 | 0 | 0.0 | 1.00 |  |
| 29 | 1 | 0.3 | 0 | 0.0 | 0 | 0.0 | 1.00 |  | 1.00 |  | 0 | 0.0 | 0 | 0.0 | 1.00 |  |
| 31 | 0 | 0.0 | 0 | 0.0 | 1 | 0.5 | 1.00 |  | 1.00 |  | 0 | 0.0 | 0 | 0.0 | 1.00 |  |
| 33A | 1 | 0.3 | 0 | 0.0 | 0 | 0.0 | 1.00 |  | 1.00 |  | 0 | 0.0 | 0 | 0.0 | 1.00 |  |
| 33B | 1 | 0.3 | 0 | 0.0 | 0 | 0.0 | 1.00 |  | 1.00 |  | 0 | 0.0 | 0 | 0.0 | 1.00 |  |
| 34 | 1 | 0.3 | 1 | 0.6 | 4 | 2.0 | 5.43E-01 | increasing | 3.75E-01 | increasing | 0 | 0.0 | 2 | 5.3 | 1.31E-01 | increase |
| 35A | 0 | 0.0 | 1 | 0.6 | 0 | 0.0 | 3.24E-01 | increasing | 4.75E-01 | decreasing | 0 | 0.0 | 0 | 0.0 | 1.00 |  |
| 35B | 1 | 0.3 | 3 | 1.7 | 6 | 3.0 | 1.02E-01 | increasing | 5.08E-01 | increasing | 4 | 6.1 | 0 | 0.0 | 2.94E-01 | decrease |
| 35C | 1 | 0.3 | 0 | 0.0 | 2 | 1.0 | 1.00 |  | 5.00E-01 | increasing | 0 | 0.0 | 2 | 5.3 | 1.31E-01 | increase |
| 35F | 5 | 1.3 | 1 | 0.6 | 2 | 1.0 | 6.69E-01 | decreasing | 1.00 |  | 1 | 1.5 | 0 | 0.0 | 1.00 |  |
| 36 | 1 | 0.3 | 0 | 0.0 | 0 | 0.0 | 1.00 |  | 1.00 |  | 0 | 0.0 | 0 | 0.0 | 1.00 |  |
| 37 | 0 | 0.0 | 1 | 0.6 | 4 | 2.0 | 3.24E-01 | increasing | 3.75E-01 | increasing | 2 | 3.0 | 1 | 2.6 | 1.00 |  |
| 38 | 2 | 0.5 | 0 | 0.0 | 1 | 0.5 | 1.00 |  | 1.00 |  | 1 | 1.5 | 0 | 0.0 | 1.00 |  |
| NT | 1 | 0.3 | 1 | 0.6 | 0 | 0.0 | 5.43E-01 | increasing | 4.75E-01 | decreasing | 0 | 0.0 | 0 | 0.0 | 1.00 |  |
| nonPCV13 | 91 | 24.5 | 41 | 23.0 | 86 | 43.7 | 7.50E-01 | decreasing | **3.03E-05** | increasing | 20 | 30.3 | 24 | 63.2 | **1.82E-03** | increase |
| total | 372 | 100.0 | 178 | 100.0 | 197 | 100.0 |  |  |  |  | 66 | 100.0 | 38 | 100.0 |  |  |

**Table E: Serotype distribution among isolates from IPD in adults (>15 years of age) in Germany (n=20,104).**

| **Serotype** | **Pre-Vaccination 1992-2006** | | **Early Vaccination 2007-2010** | | **Late Vaccination 2010-2014** | | **Pre-Vaccination vs. Early Vaccination** | | **Early Vaccination vs. Late Vaccination** | | **2010-2011** | | **2013-2014** | | **2010-2011 vs. 2013-2014** | |
| --- | --- | --- | --- | --- | --- | --- | --- | --- | --- | --- | --- | --- | --- | --- | --- | --- |
|  | **n** | **%** | **n** | **%** | **n** | **%** | **p-value** | **direction** | **p-value** | **direction** | **n** | **%** | **n** | **%** | **p-value** | **direction** |
| 4 | 368 | 8.2 | 288 | 5.0 | 164 | 1.9 | **6.15E-11** | **decreasing** | **7.61E-25** | **decreasing** | 59 | 2.6 | 31 | 1.5 | **1.42E-02** | **decreasing** |
| 6B | 183 | 4.1 | 110 | 1.9 | 49 | 0.6 | **6.99E-11** | **decreasing** | **9.38E-14** | **decreasing** | 16 | 0.7 | 11 | 0.5 | 5.65E-01 | decreasing |
| 9V | 286 | 6.4 | 203 | 3.5 | 66 | 0.8 | **2.40E-11** | **decreasing** | **1.12E-32** | **decreasing** | 32 | 1.4 | 4 | 0.2 | **5.35E-06** | **decreasing** |
| 14 | 591 | 13.2 | 367 | 6.4 | 133 | 1.5 | **1.09E-31** | **decreasing** | **2.05E-53** | **decreasing** | 41 | 1.8 | 23 | 1.1 | 7.73E-02 | decreasing |
| 18C | 105 | 2.3 | 101 | 1.8 | 78 | 0.9 | **3.94E-02** | **decreasing** | **1.02E-05** | **decreasing** | 33 | 1.4 | 14 | 0.7 | **1.82E-02** | **decreasing** |
| 19F | 153 | 3.4 | 163 | 2.8 | 131 | 1.5 | 9.47E-02 | decreasing | **9.63E-08** | **decreasing** | 54 | 2.3 | 23 | 1.1 | **2.53E-03** | **decreasing** |
| 23F | 257 | 5.7 | 194 | 3.4 | 90 | 1.0 | **8.47E-09** | **decreasing** | **3.33E-22** | **decreasing** | 38 | 1.7 | 13 | 0.6 | **1.72E-03** | **decreasing** |
| PCV7 | 1943 | 43.4 | 1426 | 24.7 | 711 | 8.2 | **3.78E-88** | **decreasing** | **5.97E-161** | **decreasing** | 273 | 11.9 | 119 | 5.8 | **1.18E-12** | **decreasing** |
| 1 | 307 | 6.9 | 487 | 8.4 | 413 | 4.8 | **2.87E-03** | **increasing** | **1.35E-18** | **decreasing** | 187 | 8.1 | 54 | 2.6 | **4.16E-16** | **decreasing** |
| 5 | 35 | 0.8 | 26 | 0.5 | 4 | 0.0 | **3.77E-02** | **decreasing** | **1.75E-07** | **decreasing** | 3 | 0.1 | 0 | 0.0 | 2.52E-01 | decreasing |
| 7F | 296 | 6.6 | 592 | 10.3 | 788 | 9.1 | **5.02E-11** | **increasing** | **2.25E-02** | **decreasing** | 294 | 12.8 | 98 | 4.8 | **4.14E-21** | **decreasing** |
| PCV10 | 2581 | 57.7 | 2531 | 43.9 | 1916 | 22.2 | **2.30E-43** | **decreasing** | **2.29E-166** | **decreasing** | 757 | 32.9 | 271 | 13.1 | **8.67E-55** | **decreasing** |
| PCV10non7 | 638 | 14.3 | 1105 | 19.2 | 1205 | 14.0 | **3.91E-11** | **increasing** | **1.15E-16** | **decreasing** | 484 | 21.0 | 152 | 7.4 | **5.37E-39** | **decreasing** |
| 3 | 389 | 8.7 | 785 | 13.6 | 1213 | 14.0 | **4.44E-15** | **increasing** | 4.76E-01 | increasing | 319 | 13.9 | 310 | 15.0 | 2.81E-01 | increasing |
| 6A | 140 | 3.1 | 174 | 3.0 | 118 | 1.4 | 7.73E-01 | decreasing | **1.19E-11** | **decreasing** | 31 | 1.3 | 22 | 1.1 | 4.11E-01 | decreasing |
| 19A | 128 | 2.9 | 349 | 6.1 | 839 | 9.7 | **7.51E-15** | **increasing** | **1.98E-15** | **increasing** | 252 | 10.9 | 145 | 7.0 | **6.98E-06** | **decreasing** |
| PCV13 | 3238 | 72.3 | 3839 | 66.6 | 4086 | 47.3 | **5.24E-10** | **decreasing** | **1.90E-116** | **decreasing** | 1359 | 59.0 | 748 | 36.3 | **2.16E-51** | **decreasing** |
| PCV13non7 | 1295 | 28.9 | 2413 | 41.9 | 3375 | 39.1 | **5.21E-42** | **increasing** | **8.68E-04** | **decreasing** | 1086 | 47.2 | 629 | 30.5 | **1.36E-29** | **decreasing** |
| PCV13non10 | 657 | 14.7 | 1308 | 22.7 | 2170 | 25.1 | **6.97E-25** | **increasing** | **8.42E-04** | **increasing** | 602 | 26.2 | 477 | 23.1 | **2.23E-02** | **decreasing** |
| 2 | 4 | 0.1 | 8 | 0.1 | 0 | 0.0 | 5.68E-01 | increasing | **6.57E-04** | **decreasing** | 0 | 0.0 | 0 | 0.0 | 1.00 |  |
| 8 | 160 | 3.6 | 171 | 3.0 | 349 | 4.0 | 9.10E-02 | decreasing | **7.29E-04** | **increasing** | 63 | 2.7 | 109 | 5.3 | **1.62E-05** | **increasing** |
| 9N | 119 | 2.7 | 178 | 3.1 | 317 | 3.7 | 2.13E-01 | increasing | 6.19E-02 | increasing | 70 | 3.0 | 92 | 4.5 | **1.59E-02** | **increasing** |
| 10A | 80 | 1.8 | 131 | 2.3 | 256 | 3.0 | 9.24E-02 | increasing | **1.16E-02** | **increasing** | 59 | 2.6 | 76 | 3.7 | **3.55E-02** | **increasing** |
| 11A | 101 | 2.3 | 126 | 2.2 | 232 | 2.7 | 8.39E-01 | decreasing | 6.32E-02 | increasing | 48 | 2.1 | 54 | 2.6 | 2.70E-01 | increasing |
| 12F | 120 | 2.7 | 112 | 1.9 | 505 | 5.8 | **1.33E-02** | **decreasing** | **1.28E-32** | **increasing** | 84 | 3.6 | 174 | 8.4 | **2.03E-11** | **increasing** |
| 15B | 24 | 0.5 | 63 | 1.1 | 97 | 1.1 | **2.25E-03** | **increasing** | 9.35E-01 | increasing | 25 | 1.1 | 19 | 0.9 | 6.50E-01 | decreasing |
| 17F | 29 | 0.6 | 16 | 0.3 | 65 | 0.8 | **6.23E-03** | **decreasing** | **1.42E-04** | **increasing** | 14 | 0.6 | 17 | 0.8 | 4.71E-01 | increasing |
| 20 | 38 | 0.8 | 41 | 0.7 | 72 | 0.8 | 4.28E-01 | decreasing | 4.42E-01 | increasing | 23 | 1.0 | 18 | 0.9 | 7.54E-01 | decreasing |
| 22F | 103 | 2.3 | 272 | 4.7 | 597 | 6.9 | **4.73E-11** | **increasing** | **4.34E-08** | **increasing** | 157 | 6.8 | 161 | 7.8 | 2.21E-01 | increasing |
| 33F | 35 | 0.8 | 65 | 1.1 | 150 | 1.7 | 8.51E-02 | increasing | **3.15E-03** | **increasing** | 44 | 1.9 | 42 | 2.0 | 8.28E-01 | increasing |
| PPV23 | 3911 | 87.4 | 4848 | 84.1 | 6608 | 76.5 | **3.38E-06** | **decreasing** | **5.00E-29** | **decreasing** | 1557 | 67.6 | 1488 | 72.2 | **1.70E-18** | **decreasing** |
| 6C | 32 | 0.7 | 99 | 1.7 | 261 | 3.0 | **4.89E-06** | **increasing** | **6.26E-07** | **increasing** | 60 | 2.6 | 50 | 2.4 | 7.72E-01 | decreasing |
| 6D | 0 | 0.0 | 2 | 0.0 | 3 | 0.0 | 5.08E-01 | increasing | 1.00 |  | 2 | 0.1 | 0 | 0.0 | 5.01E-01 | decreasing |
| 7A | 0 | 0.0 | 1 | 0.0 | 0 | 0.0 | 1.00 |  | 4.00E-01 | decreasing | 0 | 0.0 | 0 | 0.0 | 1.00 |  |
| 7B | 1 | 0.0 | 5 | 0.1 | 1 | 0.0 | 2.41E-01 | increasing | **4.11E-02** | **decreasing** | 1 | 0.0 | 0 | 0.0 | 1.00 |  |
| 7C | 6 | 0.1 | 5 | 0.1 | 8 | 0.1 | 5.50E-01 | decreasing | 1.00 |  | 2 | 0.1 | 3 | 0.1 | 6.72E-01 | increasing |
| 9A | 21 | 0.5 | 5 | 0.1 | 1 | 0.0 | **1.94E-04** | **decreasing** | **4.11E-02** | **decreasing** | 0 | 0.0 | 0 | 0.0 | 1.00 |  |
| 9L | 7 | 0.2 | 3 | 0.1 | 0 | 0.0 | 1.16E-01 | decreasing | 6.41E-02 | decreasing | 0 | 0.0 | 0 | 0.0 | 1.00 |  |
| 6F | 0 | 0.0 | 0 | 0.0 | 1 | 0.0 | 1.00 |  | 1.00 |  | 1 | 0.0 | 0 | 0.0 | 1.00 |  |
| 10B | 5 | 0.1 | 6 | 0.1 | 9 | 0.1 | 1.00 |  | 1.00 |  | 3 | 0.1 | 3 | 0.1 | 1.00 |  |
| 10F | 4 | 0.1 | 2 | 0.0 | 4 | 0.0 | 4.14E-01 | decreasing | 1.00 |  | 2 | 0.1 | 1 | 0.0 | 1.00 |  |
| 11B | 2 | 0.0 | 3 | 0.1 | 3 | 0.0 | 1.00 |  | 6.89E-01 | decreasing | 0 | 0.0 | 0 | 0.0 | 1.00 |  |
| 11C | 0 | 0.0 | 0 | 0.0 | 1 | 0.0 | 1.00 |  | 1.00 |  | 0 | 0.0 | 0 | 0.0 | 1.00 |  |
| 11F | 0 | 0.0 | 3 | 0.1 | 1 | 0.0 | 2.62E-01 | increasing | 3.09E-01 | decreasing | 1 | 0.0 | 0 | 0.0 | 1.00 |  |
| 12A | 6 | 0.1 | 3 | 0.1 | 13 | 0.2 | 1.92E-01 | decreasing | 1.23E-01 | increasing | 0 | 0.0 | 7 | 0.3 | **5.23E-03** | **increasing** |
| 12B | 1 | 0.0 | 4 | 0.1 | 0 | 0.0 | 3.94E-01 | increasing | **2.57E-02** | **decreasing** | 0 | 0.0 | 0 | 0.0 | 1.00 |  |
| 13 | 20 | 0.4 | 9 | 0.2 | 5 | 0.1 | **7.83E-03** | **decreasing** | 9.80E-02 | decreasing | 1 | 0.0 | 0 | 0.0 | 1.00 |  |
| 15A | 27 | 0.6 | 31 | 0.5 | 216 | 2.5 | 6.92E-01 | decreasing | **1.48E-21** | **increasing** | 30 | 1.3 | 75 | 3.6 | **4.85E-07** | **increasing** |
| 15C | 19 | 0.4 | 24 | 0.4 | 70 | 0.8 | 1.00 |  | **4.13E-03** | **increasing** | 15 | 0.7 | 20 | 1.0 | 3.08E-01 | increasing |
| 15F | 5 | 0.1 | 1 | 0.0 | 4 | 0.0 | 9.26E-02 | decreasing | 6.54E-01 | increasing | 1 | 0.0 | 2 | 0.1 | 6.05E-01 | increasing |
| 16F | 20 | 0.4 | 25 | 0.4 | 84 | 1.0 | 1.00 |  | **2.46E-04** | **increasing** | 12 | 0.5 | 24 | 1.2 | **2.79E-02** | **increasing** |
| 17A | 1 | 0.0 | 0 | 0.0 | 0 | 0.0 | 4.37E-01 | decreasing | 1.00 |  | 0 | 0.0 | 0 | 0.0 | 1.00 |  |
| 18A | 12 | 0.3 | 9 | 0.2 | 14 | 0.2 | 2.71E-01 | decreasing | 1.00 |  | 0 | 0.0 | 4 | 0.2 | **4.98E-02** | **increasing** |
| 18B | 1 | 0.0 | 1 | 0.0 | 4 | 0.0 | 1.00 |  | 6.54E-01 | increasing | 1 | 0.0 | 0 | 0.0 | 1.00 |  |
| 18F | 7 | 0.2 | 4 | 0.1 | 0 | 0.0 | 2.29E-01 | decreasing | **2.57E-02** | **decreasing** | 0 | 0.0 | 0 | 0.0 | 1.00 |  |
| 19B | 1 | 0.0 | 0 | 0.0 | 1 | 0.0 | 4.37E-01 | decreasing | 1.00 |  | 0 | 0.0 | 1 | 0.0 | 4.73E-01 | increasing |
| 19C | 2 | 0.0 | 0 | 0.0 | 0 | 0.0 | 1.91E-01 | decreasing | 1.00 |  | 0 | 0.0 | 0 | 0.0 | 1.00 |  |
| 21 | 0 | 0.0 | 1 | 0.0 | 5 | 0.1 | 1.00 |  | 4.12E-01 | increasing | 1 | 0.0 | 2 | 0.1 | 6.05E-01 | increasing |
| 22A | 1 | 0.0 | 2 | 0.0 | 3 | 0.0 | 1.00 |  | 1.00 |  | 3 | 0.1 | 0 | 0.0 | 2.52E-01 | decreasing |
| 23A | 31 | 0.7 | 95 | 1.6 | 215 | 2.5 | **1.13E-05** | **increasing** | **6.55E-04** | **increasing** | 46 | 2.0 | 59 | 2.9 | 7.45E-02 | increasing |
| 23B | 6 | 0.1 | 31 | 0.5 | 231 | 2.7 | **6.70E-04** | **increasing** | **3.76E-24** | **increasing** | 31 | 1.3 | 75 | 3.6 | **9.32E-07** | **increasing** |
| 24A | 2 | 0.0 | 0 | 0.0 | 2 | 0.0 | 1.91E-01 | decreasing | 5.20E-01 | increasing | 0 | 0.0 | 0 | 0.0 | 1.00 |  |
| 24B | 1 | 0.0 | 2 | 0.0 | 0 | 0.0 | 1.00E+00 |  | 1.60E-01 | decreasing | 0 | 0.0 | 0 | 0.0 | 1.00 |  |
| 24F | 47 | 1.0 | 78 | 1.4 | 204 | 2.4 | 1.74E-01 | increasing | **1.57E-05** | **increasing** | 28 | 1.2 | 78 | 3.8 | **3.18E-08** | **increasing** |
| 25A | 0 | 0.0 | 2 | 0.0 | 1 | 0.0 | 5.08E-01 | increasing | 5.68E-01 | decreasing | 1 | 0.0 | 0 | 0.0 | 1.00 |  |
| 25F | 1 | 0.0 | 3 | 0.1 | 1 | 0.0 | 6.37E-01 | increasing | 3.09E-01 | decreasing | 0 | 0.0 | 0 | 0.0 | 1.00 |  |
| 27 | 0 | 0.0 | 0 | 0.0 | 2 | 0.0 | 1.00 |  | 5.20E-01 | increasing | 1 | 0.0 | 0 | 0.0 | 1.00 |  |
| 28A | 3 | 0.1 | 9 | 0.2 | 16 | 0.2 | 2.50E-01 | increasing | 8.39E-01 | increasing | 6 | 0.3 | 2 | 0.1 | 2.95E-01 | decreasing |
| 28F | 3 | 0.1 | 3 | 0.1 | 10 | 0.1 | 1.00 |  | 2.66E-01 | increasing | 2 | 0.1 | 4 | 0.2 | 4.31E-01 | increasing |
| 29 | 3 | 0.1 | 3 | 0.1 | 7 | 0.1 | 1.00 |  | 7.49E-01 | increasing | 3 | 0.1 | 1 | 0.0 | 6.27E-01 | decreasing |
| 31 | 24 | 0.5 | 36 | 0.6 | 93 | 1.1 | 6.03E-01 | increasing | **4.97E-03** | **increasing** | 18 | 0.8 | 23 | 1.1 | 2.74E-01 | increasing |
| 33A | 8 | 0.2 | 5 | 0.1 | 0 | 0.0 | 2.64E-01 | decreasing | **1.03E-02** | **decreasing** | 0 | 0.0 | 0 | 0.0 | 1.00 |  |
| 33B | 2 | 0.0 | 2 | 0.0 | 1 | 0.0 | 1.00 |  | 5.68E-01 | decreasing | 0 | 0.0 | 0 | 0.0 | 1.00 |  |
| 34 | 15 | 0.3 | 24 | 0.4 | 42 | 0.5 | 6.28E-01 | increasing | 6.15E-01 | increasing | 9 | 0.4 | 10 | 0.5 | 6.53E-01 | increasing |
| 35A | 2 | 0.0 | 5 | 0.1 | 3 | 0.0 | 4.78E-01 | increasing | 2.80E-01 | decreasing | 1 | 0.0 | 1 | 0.0 | 1.00 |  |
| 35B | 3 | 0.1 | 32 | 0.6 | 105 | 1.2 | **8.37E-06** | **increasing** | **4.77E-05** | **increasing** | 17 | 0.7 | 34 | 1.6 | **6.81E-03** | **increasing** |
| 35C | 5 | 0.1 | 1 | 0.0 | 7 | 0.1 | 9.26E-02 | decreasing | 1.56E-01 | increasing | 2 | 0.1 | 1 | 0.0 | 1.00 |  |
| 35F | 25 | 0.6 | 91 | 1.6 | 142 | 1.6 | **6.67E-07** | **increasing** | 7.88E-01 | increasing | 29 | 1.3 | 42 | 2.0 | 5.45E-02 | increasing |
| 36 | 2 | 0.0 | 0 | 0.0 | 0 | 0.0 | 1.91E-01 | decreasing | 1.00 |  | 0 | 0.0 | 0 | 0.0 | 1.00E+00 | increasing |
| 37 | 6 | 0.1 | 0 | 0.0 | 5 | 0.1 | **6.97E-03** | **decreasing** | 1.65E-01 | increasing | 1 | 0.0 | 2 | 0.1 | 6.05E-01 | increasing |
| 38 | 26 | 0.6 | 63 | 1.1 | 96 | 1.1 | **5.23E-03** | **increasing** | 9.35E-01 | increasing | 17 | 0.7 | 25 | 1.2 | 1.22E-01 | increasing |
| 39 | 1 | 0.0 | 2 | 0.0 | 0 | 0.0 | 1.00 |  | 1.60E-01 | decreasing | 0 | 0.0 | 0 | 0.0 | 1.00 |  |
| 45 | 1 | 0.0 | 0 | 0.0 | 0 | 0.0 | 4.37E-01 | decreasing | 1.00 |  | 0 | 0.0 | 0 | 0.0 | 1.00 |  |
| 48 | 1 | 0.0 | 0 | 0.0 | 0 | 0.0 | 4.37E-01 | decreasing | 1.00 |  | 0 | 0.0 | 0 | 0.0 | 1.00 |  |
| NT | 7 | 0.2 | 7 | 0.1 | 15 | 0.2 | 7.89E-01 | decreasing | 5.17E-01 | increasing | 8 | 0.3 | 3 | 0.1 | 2.34E-01 | decreasing |
| nonPCV13 | 1239 | 27.7 | 1925 | 33.4 | 4550 | 52.7 | **5.24E-10** | **increasing** | **1.90E-116** | **increasing** | 943 | 41.0 | 1314 | 63.7 | **2.16E-51** | **increasing** |
| total | 4477 | 100.0 | 5764 | 100.0 | 8636 | 100.0 |  |  |  |  | 2302 | 100.0 | 2062 | 100.0 |  |  |

**Table F: Serotype distribution among isolates from IPD in adults (16-49 years of age) in Germany (n=3,945).**

| **Serotype** | **Pre-Vaccination 1992-2006** | | **Early Vaccination 2007-2010** | | **Late Vaccination 2010-2014** | | **Pre-Vaccination vs. Early Vaccination** | | **Early Vaccination vs. Late Vaccination** | | **2010-2011** | | **2013-2014** | | **2010-2011 vs. 2013-2014** | |
| --- | --- | --- | --- | --- | --- | --- | --- | --- | --- | --- | --- | --- | --- | --- | --- | --- |
|  | **n** | **%** | **n** | **%** | **n** | **%** | **p-value** | **direction** | **p-value** | **direction** | **n** | **%** | **n** | **%** | **p-value** | **direction** |
| 4 | 103 | 10.1 | 67 | 5.7 | 36 | 2.4 | **1.12E-04** | **decreasing** | **2.69E-05** | **decreasing** | 9 | 2.0 | 10 | 3.1 | 3.53E-01 | increasing |
| 6B | 35 | 3.4 | 10 | 0.8 | 7 | 0.5 | **2.55E-05** | **decreasing** | 3.28E-01 | decreasing | 3 | 0.7 | 0 | 0.0 | 2.70E-01 | decreasing |
| 9V | 60 | 5.9 | 32 | 2.7 | 19 | 1.3 | **2.45E-04** | **decreasing** | **9.98E-03** | **decreasing** | 10 | 2.2 | 0 | 0.0 | **6.60E-03** | **decreasing** |
| 14 | 99 | 9.7 | 73 | 6.2 | 21 | 1.4 | **2.40E-03** | **decreasing** | **4.52E-11** | **decreasing** | 2 | 0.4 | 8 | 2.5 | **2.02E-02** | **increasing** |
| 18C | 35 | 3.4 | 22 | 1.9 | 17 | 1.2 | **2.21E-02** | **decreasing** | 1.46E-01 | decreasing | 9 | 2.0 | 3 | 0.9 | 3.77E-01 | decreasing |
| 19F | 34 | 3.3 | 22 | 1.9 | 22 | 1.5 | **3.01E-02** | **decreasing** | 5.41E-01 | decreasing | 9 | 2.0 | 3 | 0.9 | 3.77E-01 | decreasing |
| 23F | 35 | 3.4 | 20 | 1.7 | 11 | 0.7 | **9.31E-03** | **decreasing** | **2.87E-02** | **decreasing** | 3 | 0.7 | 3 | 0.9 | 6.98E-01 | increasing |
| PCV7 | 401 | 39.3 | 246 | 20.8 | 133 | 9.0 | **1.54E-21** | **decreasing** | **8.97E-18** | **decreasing** | 45 | 9.9 | 27 | 8.3 | 5.31E-01 | decreasing |
| 1 | 143 | 14.0 | 214 | 18.1 | 167 | 11.3 | **1.07E-02** | **increasing** | **8.91E-07** | **decreasing** | 82 | 18.1 | 19 | 5.8 | **2.51E-07** | **decreasing** |
| 5 | 14 | 1.4 | 15 | 1.3 | 1 | 0.1 | 8.53E-01 | decreasing | **4.76E-05** | **decreasing** | 1 | 0.2 | 0 | 0.0 | 1.00 |  |
| 7F | 78 | 7.6 | 187 | 15.8 | 213 | 14.4 | **3.76E-09** | **increasing** | 3.54E-01 | decreasing | 86 | 18.9 | 25 | 7.7 | **6.49E-06** | **decreasing** |
| PCV10 | 636 | 62.4 | 662 | 55.9 | 514 | 34.8 | 2.07E-03 | decreasing | **1.44E-27** | **decreasing** | 214 | 47.1 | 71 | 21.8 | **2.95E-13** | **decreasing** |
| PCV10non7 | 235 | 23.0 | 416 | 35.1 | 381 | 25.8 | **5.82E-10** | **increasing** | **2.02E-07** | **decreasing** | 169 | 37.2 | 44 | 13.5 | **6.44E-14** | **decreasing** |
| 3 | 49 | 4.8 | 98 | 8.3 | 131 | 8.9 | **1.11E-03** | **increasing** | 6.27E-01 | increasing | 35 | 7.7 | 30 | 9.2 | 5.12E-01 | increasing |
| 6A | 13 | 1.3 | 17 | 1.4 | 9 | 0.6 | 8.54E-01 | increasing | **4.54E-02** | **decreasing** | 1 | 0.2 | 1 | 0.3 | 1.00 |  |
| 19A | 26 | 2.5 | 57 | 4.8 | 105 | 7.1 | **6.75E-03** | **increasing** | **1.43E-02** | **increasing** | 28 | 6.2 | 15 | 4.6 | 4.27E-01 | decreasing |
| PCV13 | 724 | 71.0 | 834 | 70.4 | 759 | 51.4 | 7.78E-01 | decreasing | **1.87E-23** | **decreasing** | 278 | 61.2 | 117 | 36.0 | **3.95E-12** | **decreasing** |
| PCV13non7 | 323 | 31.7 | 588 | 49.6 | 626 | 42.4 | **1.08E-17** | **increasing** | **2.00E-04** | **decreasing** | 233 | 51.3 | 90 | 27.7 | **4.09E-11** | **decreasing** |
| PCV13non10 | 88 | 8.6 | 172 | 14.5 | 245 | 16.6 | **2.08E-05** | **increasing** | 1.48E-01 | increasing | 64 | 14.1 | 46 | 14.2 | 1.00 |  |
| 2 | 3 | 0.3 | 2 | 0.2 | 0 | 0.0 | 6.68E-01 | decreasing | 1.98E-01 | decreasing | 0 | 0.0 | 0 | 0.0 | 1.00 |  |
| 8 | 49 | 4.8 | 48 | 4.1 | 68 | 4.6 | 4.06E-01 | decreasing | 5.05E-01 | increasing | 16 | 3.5 | 23 | 7.1 | 3.00E-02 | increasing |
| 9N | 25 | 2.5 | 28 | 2.4 | 55 | 3.7 | 8.90E-01 | decreasing | 5.59E-02 | increasing | 17 | 3.7 | 17 | 5.2 | 3.75E-01 | increasing |
| 10A | 18 | 1.8 | 29 | 2.4 | 40 | 2.7 | 3.02E-01 | increasing | 7.14E-01 | increasing | 13 | 2.9 | 14 | 4.3 | 3.22E-01 | increasing |
| 11A | 18 | 1.8 | 16 | 1.4 | 32 | 2.2 | 4.90E-01 | decreasing | 1.42E-01 | increasing | 7 | 1.5 | 5 | 1.5 | 1.00 |  |
| 12F | 33 | 3.2 | 27 | 2.3 | 120 | 8.1 | 1.90E-01 | decreasing | 9.55E-12 | increasing | 18 | 4.0 | 40 | 12.3 | 2.16E-05 | increasing |
| 15B | 2 | 0.2 | 7 | 0.6 | 14 | 0.9 | 1.90E-01 | increasing | 3.80E-01 | increasing | 4 | 0.9 | 0 | 0.0 | 1.45E-01 | decreasing |
| 17F | 6 | 0.6 | 6 | 0.5 | 12 | 0.8 | 1.00 |  | 4.76E-01 | increasing | 4 | 0.9 | 3 | 0.9 | 1.00 |  |
| 20 | 10 | 1.0 | 9 | 0.8 | 13 | 0.9 | 6.47E-01 | decreasing | 8.31E-01 | increasing | 4 | 0.9 | 4 | 1.2 | 7.25E-01 | increasing |
| 22F | 22 | 2.2 | 52 | 4.4 | 82 | 5.6 | **4.18E-03** | **increasing** | 1.82E-01 | increasing | 24 | 5.3 | 22 | 6.8 | 4.42E-01 | increasing |
| 33F | 9 | 0.9 | 14 | 1.2 | 21 | 1.4 | 5.35E-01 | increasing | 6.13E-01 | increasing | 6 | 1.3 | 7 | 2.2 | 4.05E-01 | increasing |
| PPV23 | 906 |  | 1055 |  | 1207 |  | 8.92E-01 | increasing | 1.44E-07 | decreasing | 390 |  | 251 |  | 2.26E-03 | decreasing |
| 6C | 2 | 0.2 | 9 | 0.8 | 23 | 1.6 | 7.29E-02 | increasing | 7.31E-02 | increasing | 4 | 0.9 | 5 | 1.5 | 5.02E-01 | increasing |
| 6D | 0 | 0.0 | 0 | 0.0 | 1 | 0.1 | 1.00 |  | 1.00 |  | 1 | 0.2 | 0 | 0.0 | 1.00 |  |
| 7C | 1 | 0.1 | 0 | 0.0 | 1 | 0.1 | 4.63E-01 | decreasing | 1.00 |  | 0 | 0.0 | 1 | 0.3 | 4.17E-01 | increasing |
| 9A | 5 | 0.5 | 1 | 0.1 | 0 | 0.0 | 1.02E-01 | decreasing | 4.45E-01 | decreasing | 0 | 0.0 | 0 | 0.0 | 1.00 |  |
| 9L | 2 | 0.2 | 0 | 0.0 | 0 | 0.0 | 2.14E-01 | decreasing | 1.00 |  | 0 | 0.0 | 0 | 0.0 | 1.00 |  |
| 10B | 2 | 0.2 | 0 | 0.0 | 1 | 0.1 | 2.14E-01 | decreasing | 1.00 |  | 1 | 0.2 | 0 | 0.0 | 1.00 |  |
| 10F | 0 | 0.0 | 0 | 0.0 | 1 | 0.1 | 1.00 |  | 1.00 |  | 1 | 0.2 | 0 | 0.0 | 1.00 |  |
| 11B | 0 | 0.0 | 2 | 0.2 | 1 | 0.1 | 5.03E-01 | increasing | 5.89E-01 | decreasing | 0 | 0.0 | 0 | 0.0 | 1.00 |  |
| 11F | 0 | 0.0 | 1 | 0.1 | 0 | 0.0 | 1.00 |  | 4.45E-01 | decreasing | 0 | 0.0 | 0 | 0.0 | 1.00 |  |
| 12A | 2 | 0.2 | 1 | 0.1 | 2 | 0.1 | 5.99E-01 | decreasing | 1.00E+00 | increasing | 0 | 0.0 | 1 | 0.3 | 4.17E-01 | increasing |
| 12B | 1 | 0.1 | 1 | 0.1 | 0 | 0.0 | 1.00 |  | 4.45E-01 | decreasing | 0 | 0.0 | 0 | 0.0 | 1.00 |  |
| 13 | 11 | 1.1 | 1 | 0.1 | 3 | 0.2 | **1.96E-03** | **decreasing** | 6.34E-01 | increasing | 1 | 0.2 | 0 | 0.0 | 1.00 |  |
| 15A | 8 | 0.8 | 4 | 0.3 | 20 | 1.4 | 2.45E-01 | decreasing | **6.29E-03** | **increasing** | 4 | 0.9 | 7 | 2.2 | 2.16E-01 | increasing |
| 15C | 2 | 0.2 | 7 | 0.6 | 18 | 1.2 | 1.90E-01 | increasing | 1.08E-01 | increasing | 4 | 0.9 | 7 | 2.2 | 2.16E-01 | increasing |
| 15F | 1 | 0.1 | 0 | 0.0 | 2 | 0.1 | 4.63E-01 | decreasing | 5.06E-01 | increasing | 1 | 0.2 | 0 | 0.0 | 1.00 |  |
| 16F | 6 | 0.6 | 6 | 0.5 | 8 | 0.5 | 1.00 |  | 1.00 |  | 3 | 0.7 | 1 | 0.3 | 6.44E-01 | decreasing |
| 18A | 2 | 0.2 | 2 | 0.2 | 2 | 0.1 | 1.00 |  | 1.00 |  | 0 | 0.0 | 0 | 0.0 | 1.00 |  |
| 18B | 0 | 0.0 | 1 | 0.1 | 1 | 0.1 | 1.00 |  | 1.00 |  | 0 | 0.0 | 0 | 0.0 | 1.00 |  |
| 18F | 5 | 0.5 | 2 | 0.2 | 0 | 0.0 | 2.60E-01 | decreasing | 1.98E-01 | decreasing | 0 | 0.0 | 0 | 0.0 | 1.00 |  |
| 19B | 0 | 0.0 | 0 | 0.0 | 1 | 0.1 | 1.00 |  | 1.00 |  | 0 | 0.0 | 1 | 0.3 | 4.17E-01 | increasing |
| 21 | 0 | 0.0 | 1 | 0.1 | 1 | 0.1 | 1.00 |  | 1.00 |  | 0 | 0.0 | 1 | 0.3 | 4.17E-01 | increasing |
| 22A | 0 | 0.0 | 1 | 0.1 | 2 | 0.1 | 1.00 |  | 1.00 |  | 2 | 0.4 | 0 | 0.0 | 5.13E-01 | decreasing |
| 23A | 5 | 0.5 | 13 | 1.1 | 29 | 2.0 | 1.54E-01 | increasing | 8.54E-02 | increasing | 10 | 2.2 | 4 | 1.2 | 4.16E-01 | decreasing |
| 23B | 0 | 0.0 | 5 | 0.4 | 55 | 3.7 | 6.57E-02 | increasing | **1.07E-09** | **increasing** | 9 | 2.0 | 16 | 4.9 | **2.43E-02** | **increasing** |
| 24A | 1 | 0.1 | 0 | 0.0 | 0 | 0.0 | 4.63E-01 | decreasing | 1.00 |  | 0 | 0.0 | 0 | 0.0 | 1.00 |  |
| 24B | 0 | 0.0 | 1 | 0.1 | 0 | 0.0 | 1.00 |  | 4.45E-01 | decreasing | 0 | 0.0 | 0 | 0.0 | 1.00 |  |
| 24F | 11 | 1.1 | 12 | 1.0 | 19 | 1.3 | 1.00 |  | 5.88E-01 | increasing | 3 | 0.7 | 7 | 2.2 | 1.03E-01 | increasing |
| 25F | 0 | 0.0 | 1 | 0.1 | 0 | 0.0 | 1.00 |  | 4.45E-01 | decreasing | 0 | 0.0 | 0 | 0.0 | 1.00 |  |
| 27 | 0 | 0.0 | 0 | 0.0 | 1 | 0.1 | 1.00 |  | 1.00 |  | 0 | 0.0 | 0 | 0.0 | 1.00 |  |
| 28A | 1 | 0.1 | 0 | 0.0 | 3 | 0.2 | 4.63E-01 | decreasing | 2.59E-01 | increasing | 1 | 0.2 | 1 | 0.3 | 1.00 |  |
| 28F | 1 | 0.1 | 1 | 0.1 | 0 | 0.0 | 1.00 |  | 4.45E-01 | decreasing | 0 | 0.0 | 0 | 0.0 | 1.00 |  |
| 29 | 0 | 0.0 | 1 | 0.1 | 1 | 0.1 | 1.00 |  | 1.00 |  | 1 | 0.2 | 0 | 0.0 | 1.00 |  |
| 31 | 6 | 0.6 | 7 | 0.6 | 7 | 0.5 | 1.00 |  | 7.90E-01 | decreasing | 3 | 0.7 | 0 | 0.0 | 2.70E-01 | increasing |
| 33A | 1 | 0.1 | 0 | 0.0 | 0 | 0.0 | 4.63E-01 | decreasing | 1.00 |  | 0 | 0.0 | 0 | 0.0 | 1.00 |  |
| 33B | 1 | 0.1 | 0 | 0.0 | 0 | 0.0 | 4.63E-01 | decreasing | 1.00 |  | 0 | 0.0 | 0 | 0.0 | 1.00 |  |
| 34 | 3 | 0.3 | 5 | 0.4 | 6 | 0.4 | 7.32E-01 | increasing | 1.00 |  | 0 | 0.0 | 2 | 0.6 | 1.74E-01 | increasing |
| 35A | 1 | 0.1 | 1 | 0.1 | 2 | 0.1 | 1.00 |  | 1.00 |  | 0 | 0.0 | 1 | 0.3 | 4.17E-01 | increasing |
| 35B | 0 | 0.0 | 3 | 0.3 | 10 | 0.7 | 2.54E-01 | increasing | 1.63E-01 | increasing | 1 | 0.2 | 3 | 0.9 | 3.14E-01 | increasing |
| 35C | 2 | 0.2 | 0 | 0.0 | 1 | 0.1 | 2.14E-01 | decreasing | 1.00 |  | 1 | 0.2 | 0 | 0.0 | 1.00 |  |
| 35F | 8 | 0.8 | 16 | 1.4 | 25 | 1.7 | 2.23E-01 | increasing | 5.29E-01 | increasing | 3 | 0.7 | 13 | 4.0 | **1.52E-03** | **increasing** |
| 37 | 2 | 0.2 | 0 | 0.0 | 1 | 0.1 | 2.14E-01 | decreasing | 1.00 |  | 0 | 0.0 | 1 | 0.3 | 4.17E-01 | decreasing |
| 38 | 4 | 0.4 | 5 | 0.4 | 5 | 0.3 | 1.00 |  | 7.59E-01 | decreasing | 2 | 0.4 | 0 | 0.0 | 5.13E-01 | decreasing |
| 39 | 1 | 0.1 | 1 | 0.1 | 0 | 0.0 | 1.00 |  | 4.45E-01 | decreasing | 0 | 0.0 | 0 | 0.0 | 1.00 |  |
| 45 | 1 | 0.1 | 0 | 0.0 | 0 | 0.0 | 4.63E-01 | decreasing | 1.00 |  | 0 | 0.0 | 0 | 0.0 | 1.00 |  |
| NT | 2 | 0.2 | 1 | 0.1 | 8 | 0.5 | 5.99E-01 | decreasing | **4.90E-02** | **increasing** | 7 | 1.5 | 1 | 0.3 | 1.49E-01 | decreasing |
| nonPCV13 | 296 | 29.0 | 351 | 29.6 | 718 | 48.6 | 7.78E-01 | increasing | **1.87E-23** | **increasing** | 176 | 38.8 | 208 | 64.0 | **3.95E-12** | **increasing** |
| total | 1020 | 100.0 | 1185 | 100.0 | 1477 | 100.0 |  |  |  |  | 454 | 100.0 | 325 | 100.0 |  |  |

**Table G: Serotype distribution among isolates from IPD in adults (50-60 years of age) in Germany (n=3,285)**.

| **Serotype** | **Pre-Vaccination 1992-2006** | | **Early Vaccination 2007-2010** | | **Late Vaccination 2010-2014** | | **Pre-Vaccination vs. Early Vaccination** | | **Early Vaccination vs. Late Vaccination** | | **2010-2011** | | **2013-2014** | | **2010-2011 vs. 2013-2014** | |
| --- | --- | --- | --- | --- | --- | --- | --- | --- | --- | --- | --- | --- | --- | --- | --- | --- |
|  | **n** | **%** | **n** | **%** | **n** | **%** | **p-value** | **direction** | **p-value** | **direction** | **n** | **%** | **n** | **%** | **p-value** | **direction** |
| 4 | 59 | 7.9 | 49 | 5.2 | 29 | 2.1 | **2.71E-02** | **decreasing** | **5.10E-05** | **decreasing** | 11 | 3.0 | 5 | 1.4 | 2.07E-01 | decreasing |
| 6B | 31 | 4.2 | 15 | 1.6 | 9 | 0.6 | **1.41E-03** | **decreasing** | **3.46E-02** | **decreasing** | 2 | 0.5 | 2 | 0.6 | 1.00 |  |
| 9V | 43 | 5.8 | 40 | 4.2 | 15 | 1.1 | 1.73E-01 | decreasing | **9.98E-07** | **decreasing** | 5 | 1.4 | 1 | 0.3 | 2.18E-01 | decreasing |
| 14 | 103 | 13.9 | 50 | 5.3 | 24 | 1.7 | **1.32E-09** | **decreasing** | **1.61E-06** | **decreasing** | 7 | 1.9 | 4 | 1.1 | 5.47E-01 | decreasing |
| 18C | 18 | 2.4 | 18 | 1.9 | 2 | 0.1 | 5.00E-01 | decreasing | **5.03E-06** | **decreasing** | 1 | 0.3 | 0 | 0.0 | 1.00 |  |
| 19F | 34 | 4.6 | 23 | 2.4 | 17 | 1.2 | **2.03E-02** | **decreasing** | **3.33E-02** | **decreasing** | 8 | 2.2 | 3 | 0.9 | 2.25E-01 | decreasing |
| 23F | 39 | 5.2 | 34 | 3.6 | 6 | 0.4 | 1.17E-01 | decreasing | **6.14E-09** | **decreasing** | 4 | 1.1 | 2 | 0.6 | 6.87E-01 | decreasing |
| PCV7 | 327 | 44.0 | 229 | 24.2 | 102 | 7.3 | **1.24E-17** | **decreasing** | **1.42E-30** | **decreasing** | 38 | 10.4 | 17 | 4.9 | **7.24E-03** | **decreasing** |
| 1 | 41 | 5.5 | 93 | 9.8 | 86 | 6.1 | **1.06E-03** | **increasing** | **1.10E-03** | **decreasing** | 41 | 11.2 | 12 | 3.4 | **8.40E-05** | **decreasing** |
| 5 | 8 | 1.1 | 2 | 0.2 | 0 | 0.0 | **2.63E-02** | **decreasing** | 1.61E-01 | decreasing | 0 | 0.0 | 0 | 0.0 | 1.00 |  |
| 7F | 53 | 7.1 | 97 | 10.3 | 120 | 8.5 | **2.52E-02** | **increasing** | 1.67E-01 | decreasing | 45 | 12.3 | 10 | 2.9 | **1.82E-06** | **decreasing** |
| PCV10 | 429 | 57.7 | 421 | 44.6 | 308 | 21.9 | **8.71E-08** | **decreasing** | **6.58E-31** | **decreasing** | 124 | 33.8 | 39 | 11.2 | **2.36E-13** | **decreasing** |
| PCV10non7 | 102 | 13.7 | 192 | 20.3 | 206 | 14.7 | **3.75E-04** | **increasing** | **4.02E-04** | **decreasing** | 86 | 23.4 | 22 | 6.3 | **6.85E-11** | **decreasing** |
| 3 | 57 | 7.7 | 122 | 12.9 | 184 | 13.1 | **5.86E-04** | **increasing** | 9.50E-01 | increasing | 57 | 15.5 | 50 | 14.3 | 6.76E-01 | decreasing |
| 6A | 26 | 3.5 | 20 | 2.1 | 16 | 1.1 | 9.75E-02 | decreasing | 6.15E-02 | decreasing | 5 | 1.4 | 4 | 1.1 | 1.00 |  |
| 19A | 18 | 2.4 | 54 | 5.7 | 134 | 9.5 | **9.35E-04** | **increasing** | **8.19E-04** | **increasing** | 49 | 13.4 | 22 | 6.3 | **1.69E-03** | **decreasing** |
| PCV13 | 530 | 71.3 | 617 | 65.3 | 642 | 45.7 | **8.63E-03** | **decreasing** | **5.87E-21** | **decreasing** | 235 | 64.0 | 115 | 33.0 | **6.54E-17** | **decreasing** |
| PCV13non7 | 203 | 27.3 | 388 | 41.1 | 540 | 38.4 | **4.30E-09** | **increasing** | 2.12E-01 | decreasing | 197 | 53.7 | 98 | 28.1 | **3.57E-12** | **decreasing** |
| PCV13non10 | 101 | 13.6 | 196 | 20.7 | 334 | 23.8 | **1.40E-04** | **increasing** | 8.73E-02 | increasing | 111 | 30.2 | 76 | 21.8 | **1.07E-02** | **decreasing** |
| 2 | 0 | 0.0 | 1 | 0.1 | 0 | 0.0 | 1.00 |  | 4.02E-01 | decreasing | 0 | 0.0 | 0 | 0.0 | 1.00 |  |
| 8 | 22 | 3.0 | 28 | 3.0 | 75 | 5.3 | 1.00 |  | **5.49E-03** | **increasing** | 8 | 2.2 | 18 | 5.2 | **4.43E-02** | **increasing** |
| 9N | 19 | 2.6 | 29 | 3.1 | 55 | 3.9 | 5.59E-01 | increasing | 3.09E-01 | increasing | 6 | 1.6 | 17 | 4.9 | **1.83E-02** | **increasing** |
| 10A | 17 | 2.3 | 21 | 2.2 | 56 | 4.0 | 1.00 |  | **1.82E-02** | **increasing** | 14 | 3.8 | 17 | 4.9 | 5.83E-01 | increasing |
| 11A | 24 | 3.2 | 26 | 2.8 | 38 | 2.7 | 5.67E-01 | decreasing | 1.00 |  | 5 | 1.4 | 9 | 2.6 | 2.87E-01 | increasing |
| 12F | 22 | 3.0 | 27 | 2.9 | 110 | 7.8 | 1.00 |  | **2.09E-07** | **increasing** | 15 | 4.1 | 44 | 12.6 | **3.38E-05** | **increasing** |
| 15B | 3 | 0.4 | 16 | 1.7 | 12 | 0.9 | **1.77E-02** | **increasing** | 8.05E-02 | decreasing | 5 | 1.4 | 1 | 0.3 | 2.18E-01 | decreasing |
| 17F | 5 | 0.7 | 2 | 0.2 | 11 | 0.8 | 2.52E-01 | decreasing | 8.86E-02 | increasing | 4 | 1.1 | 2 | 0.6 | 6.87E-01 | decreasing |
| 20 | 7 | 0.9 | 14 | 1.5 | 12 | 0.9 | 3.81E-01 | increasing | 1.64E-01 | decreasing | 5 | 1.4 | 3 | 0.9 | 7.26E-01 | decreasing |
| 22F | 20 | 2.7 | 54 | 5.7 | 96 | 6.8 | **2.61E-03** | **increasing** | 3.02E-01 | increasing | 25 | 6.8 | 27 | 7.7 | 6.67E-01 | increasing |
| 33F | 6 | 0.8 | 9 | 1.0 | 22 | 1.6 | 8.00E-01 | increasing | 2.68E-01 | increasing | 6 | 1.6 | 6 | 1.7 | 1.00 |  |
| PPV23 | 649 | 87.3 | 824 | 87.2 | 1113 | 79.2 | 9.42E-01 | decreasing | **4.59E-07** | **decreasing** | 323 | 88.0 | 255 | 73.1 | **4.10E-07** | **decreasing** |
| 6C | 4 | 0.5 | 12 | 1.3 | 17 | 1.2 | 1.37E-01 | increasing | 1.00 |  | 3 | 0.8 | 3 | 0.9 | 1.00 |  |
| 6D | 0 | 0.0 | 0 | 0.0 | 0 | 0.0 | 1.00 |  | 1.00 |  | 0 | 0.0 | 0 | 0.0 | 1.00 |  |
| 7A | 0 | 0.0 | 1 | 0.1 | 0 | 0.0 | 1.00 |  | 4.02E-01 | decreasing | 0 | 0.0 | 0 | 0.0 | 1.00 |  |
| 7B | 0 | 0.0 | 1 | 0.1 | 1 | 0.1 | 1.00 |  | 1.00 |  | 1 | 0.3 | 0 | 0.0 | 1.00 |  |
| 7C | 3 | 0.4 | 1 | 0.1 | 3 | 0.2 | 3.26E-01 | decreasing | 6.53E-01 | increasing | 1 | 0.3 | 1 | 0.3 | 1.00 |  |
| 9A | 4 | 0.5 | 0 | 0.0 | 0 | 0.0 | **3.74E-02** | **decreasing** | 1.00 |  | 0 | 0.0 | 0 | 0.0 | 1.00 |  |
| 9L | 1 | 0.1 | 0 | 0.0 | 0 | 0.0 | 4.40E-01 | decreasing | 1.00 |  | 0 | 0.0 | 0 | 0.0 | 1.00 |  |
| 10B | 0 | 0.0 | 1 | 0.1 | 1 | 0.1 | 1.00 |  | 1.00 |  | 0 | 0.0 | 1 | 0.3 | 4.87E-01 | increasing |
| 10F | 2 | 0.3 | 0 | 0.0 | 0 | 0.0 | 1.94E-01 | decreasing | 1.00 |  | 0 | 0.0 | 0 | 0.0 | 1.00 |  |
| 11B | 1 | 0.1 | 0 | 0.0 | 1 | 0.1 | 4.40E-01 | decreasing | 1.00 |  | 0 | 0.0 | 0 | 0.0 | 1.00 |  |
| 11F | 0 | 0.0 | 0 | 0.0 | 1 | 0.1 | 1.00 |  | 1.00 |  | 1 | 0.3 | 0 | 0.0 | 1.00 |  |
| 12A | 0 | 0.0 | 0 | 0.0 | 3 | 0.2 | 1.00 |  | 2.79E-01 | increasing | 0 | 0.0 | 1 | 0.3 | 4.87E-01 | increasing |
| 12B | 0 | 0.0 | 0 | 0.0 | 0 | 0.0 | 1.00 |  | 1.00 |  | 0 | 0.0 | 0 | 0.0 | 1.00 |  |
| 13 | 2 | 0.3 | 2 | 0.2 | 1 | 0.1 | 1.00 | decreasing | 5.68E-01 | decreasing | 0 | 0.0 | 0 | 0.0 | 1.00 |  |
| 15A | 1 | 0.1 | 6 | 0.6 | 32 | 2.3 | 1.43E-01 | increasing | **1.40E-03** | **increasing** | 5 | 1.4 | 7 | 2.0 | 5.70E-01 | increasing |
| 15C | 3 | 0.4 | 5 | 0.5 | 17 | 1.2 | 1.00 |  | 1.25E-01 | increasing | 2 | 0.5 | 7 | 2.0 | 9.95E-02 | increasing |
| 15F | 0 | 0.0 | 0 | 0.0 | 1 | 0.1 | 1.00 |  | 1.00 |  | 0 | 0.0 | 1 | 0.3 | 4.87E-01 | increasing |
| 16F | 6 | 0.8 | 2 | 0.2 | 12 | 0.9 | 1.49E-01 | decreasing | 5.60E-02 | increasing | 2 | 0.5 | 2 | 0.6 | 1.00 |  |
| 18A | 2 | 0.3 | 0 | 0.0 | 2 | 0.1 | 1.94E-01 | decreasing | 5.19E-01 | increasing | 0 | 0.0 | 2 | 0.6 | 2.37E-01 | increasing |
| 18B | 0 | 0.0 | 0 | 0.0 | 0 | 0.0 | 1.00 |  | 1.00 |  | 0 | 0.0 | 0 | 0.0 | 1.00 |  |
| 18F | 0 | 0.0 | 1 | 0.1 | 0 | 0.0 | 1.00 |  | 4.02E-01 | decreasing | 0 | 0.0 | 0 | 0.0 | 1.00 |  |
| 21 | 0 | 0.0 | 0 | 0.0 | 1 | 0.1 | 1.00 |  | 1.00 |  | 1 | 0.3 | 0 | 0.0 | 1.00 |  |
| 22A | 0 | 0.0 | 1 | 0.1 | 0 | 0.0 | 1.00 |  | 4.02E-01 | decreasing | 0 | 0.0 | 0 | 0.0 | 1.00 |  |
| 23A | 6 | 0.8 | 16 | 1.7 | 31 | 2.2 | 1.32E-01 | increasing | 4.53E-01 | increasing | 6 | 1.6 | 8 | 2.3 | 5.96E-01 | increasing |
| 23B | 0 | 0.0 | 5 | 0.5 | 46 | 3.3 | 7.11E-02 | increasing | **1.79E-06** | **increasing** | 7 | 1.9 | 17 | 4.9 | 3.64E-02 | increasing |
| 24A | 0 | 0.0 | 0 | 0.0 | 0 | 0.0 | 1.00 |  | 1.00 |  | 0 | 0.0 | 0 | 0.0 | 1.00 |  |
| 24B | 0 | 0.0 | 0 | 0.0 | 0 | 0.0 | 1.00 |  | 1.00 |  | 0 | 0.0 | 0 | 0.0 | 1.00 |  |
| 24F | 5 | 0.7 | 9 | 1.0 | 29 | 2.1 | 5.98E-01 | increasing | **4.43E-02** | **increasing** | 1 | 0.3 | 13 | 3.7 | **6.84E-04** | **increasing** |
| 25A | 0 | 0.0 | 0 | 0.0 | 1 | 0.1 | 1.00 |  | 1.00 |  | 1 | 0.3 | 0 | 0.0 | 1.00 |  |
| 25F | 0 | 0.0 | 1 | 0.1 | 1 | 0.1 | 1.00 |  | 1.00 |  | 0 | 0.0 | 0 | 0.0 | 1.00 |  |
| 27 | 0 | 0.0 | 0 | 0.0 | 0 | 0.0 | 1.00 |  | 1.00 |  | 0 | 0.0 | 0 | 0.0 | 1.00 |  |
| 28A | 1 | 0.1 | 2 | 0.2 | 4 | 0.3 | 1.00 |  | 1.00 |  | 0 | 0.0 | 1 | 0.3 | 4.87E-01 | increasing |
| 28F | 1 | 0.1 | 1 | 0.1 | 3 | 0.2 | 1.00 |  | 6.53E-01 | increasing | 0 | 0.0 | 2 | 0.6 | 2.37E-01 | increasing |
| 29 | 2 | 0.3 | 0 | 0.0 | 0 | 0.0 | 1.94E-01 | decreasing | 1.00 |  | 0 | 0.0 | 0 | 0.0 | 1.00 |  |
| 31 | 5 | 0.7 | 3 | 0.3 | 6 | 0.4 | 3.11E-01 | decreasing | 7.48E-01 | increasing | 0 | 0.0 | 2 | 0.6 | 2.37E-01 | increasing |
| 33A | 1 | 0.1 | 0 | 0.0 | 0 | 0.0 | 4.40E-01 | decreasing | 1.00 |  | 0 | 0.0 | 0 | 0.0 | 1.00 |  |
| 33B | 0 | 0.0 | 1 | 0.1 | 0 | 0.0 | 1.00 |  | 4.02E-01 | decreasing | 0 | 0.0 | 0 | 0.0 | 1.00 |  |
| 34 | 3 | 0.4 | 2 | 0.2 | 7 | 0.5 | 6.60E-01 | decreasing | 3.29E-01 | increasing | 0 | 0.0 | 2 | 0.6 | 2.37E-01 | increasing |
| 35A | 0 | 0.0 | 1 | 0.1 | 1 | 0.1 | 1.00 |  | 1.00 |  | 1 | 0.3 | 0 | 0.0 | 1.00 |  |
| 35B | 0 | 0.0 | 5 | 0.5 | 17 | 1.2 | 7.11E-02 | increasing | 1.25E-01 | increasing | 0 | 0.0 | 9 | 2.6 | **1.47E-03** | **increasing** |
| 35C | 0 | 0.0 | 0 | 0.0 | 1 | 0.1 | 1.00 |  | 1.00 |  | 1 | 0.3 | 0 | 0.0 | 1.00 |  |
| 35F | 3 | 0.4 | 17 | 1.8 | 21 | 1.5 | **1.08E-02** | **increasing** | 6.18E-01 | decreasing | 5 | 1.4 | 5 | 1.4 | 1.00 |  |
| 36 | 1 | 0.1 | 0 | 0.0 | 0 | 0.0 | 4.40E-01 | decreasing | 1.00 |  | 0 | 0.0 | 0 | 0.0 | 1.00 |  |
| 37 | 1 | 0.1 | 0 | 0.0 | 0 | 0.0 | 4.40E-01 | decreasing | 1.00 |  | 0 | 0.0 | 0 | 0.0 | 1.00 |  |
| 38 | 8 | 1.1 | 4 | 0.4 | 12 | 0.9 | 1.46E-01 | decreasing | 3.07E-01 | increasing | 0 | 0.0 | 5 | 1.4 | **2.71E-02** | **increasing** |
| 39 | 0 | 0.0 | 0 | 0.0 | 0 | 0.0 | 1.00 |  | 1.00 |  | 0 | 0.0 | 0 | 0.0 | 1.00 |  |
| 45 | 0 | 0.0 | 0 | 0.0 | 0 | 0.0 | 1.00 |  | 1.00 |  | 0 | 0.0 | 0 | 0.0 | 1.00 |  |
| NT | 2 | 0.3 | 1 | 0.1 | 4 | 0.3 | 5.86E-01 | decreasing | 6.54E-01 | increasing | 1 | 0.3 | 1 | 0.3 | 1.00 |  |
| nonPCV13 | 213 | 28.7 | 328 | 34.7 | 764 | 54.3 | 8.63E-03 | increasing | **5.87E-21** | **increasing** | 132 | 36.0 | 234 | 67.0 | **6.54E-17** | **increasing** |
| total | 743 | 100.0 | 945 | 100.0 | 1406 | 100.0 |  |  |  |  | 367 | 100.0 | 349 | 100.0 |  |  |

**Table H: Serotype distribution among isolates from IPD in adults (61-75 years of age) in Germany (n=6,902)**.

| **Serotype** | **Pre-Vaccination 1992-2006** | | **Early Vaccination 2007-2010** | | **Late Vaccination 2010-2014** | | **Pre-Vaccination vs. Early Vaccination** | | **Early Vaccination vs. Late Vaccination** | | **2010-2011** | | **2013-2014** | | **2010-2011 vs. 2013-2014** | |
| --- | --- | --- | --- | --- | --- | --- | --- | --- | --- | --- | --- | --- | --- | --- | --- | --- |
|  | **n** | **%** | **n** | **%** | **n** | **%** | **p-value** | **direction** | **p-value** | **direction** | **n** | **%** | **n** | **%** | **p-value** | **direction** |
| 4 | 126 | 8.1 | 96 | 4.9 | 61 | 2.0 | **1.56E-04** | **decreasing** | **2.81E-08** | **decreasing** | 25 | 3.1 | 8 | 1.1 | **7.93E-03** | **decreasing** |
| 6B | 69 | 4.4 | 42 | 2.2 | 14 | 0.5 | **1.97E-04** | **decreasing** | **1.01E-07** | **decreasing** | 6 | 0.7 | 4 | 0.6 | 7.57E-01 | decreasing |
| 9V | 109 | 7.0 | 73 | 3.8 | 13 | 0.4 | **2.20E-05** | **decreasing** | **2.58E-18** | **decreasing** | 5 | 0.6 | 1 | 0.1 | 2.23E-01 | decreasing |
| 14 | 204 | 13.1 | 124 | 6.4 | 47 | 1.6 | **1.28E-11** | **decreasing** | **7.54E-19** | **decreasing** | 20 | 2.5 | 4 | 0.6 | **3.06E-03** | **decreasing** |
| 18C | 27 | 1.7 | 29 | 1.5 | 30 | 1.0 | 5.90E-01 | decreasing | 1.41E-01 | decreasing | 10 | 1.2 | 5 | 0.7 | 3.12E-01 | decreasing |
| 19F | 52 | 3.4 | 55 | 2.8 | 43 | 1.4 | 4.29E-01 | decreasing | **7.93E-04** | **decreasing** | 18 | 2.2 | 7 | 1.0 | 6.81E-02 | decreasing |
| 23F | 88 | 5.7 | 67 | 3.5 | 31 | 1.0 | **1.66E-03** | **decreasing** | **6.75E-09** | **decreasing** | 18 | 2.2 | 5 | 0.7 | **1.88E-02** | **decreasing** |
| PCV7 | 675 | 43.5 | 486 | 25.0 | 239 | 8.0 | **1.72E-30** | **decreasing** | **2.44E-59** | **decreasing** | 102 | 12.7 | 34 | 4.8 | **4.24E-08** | **decreasing** |
| 1 | 83 | 5.3 | 114 | 5.9 | 118 | 4.0 | 5.55E-01 | increasing | **2.41E-03** | **decreasing** | 47 | 5.9 | 18 | 2.5 | **1.38E-03** | **decreasing** |
| 5 | 9 | 0.6 | 8 | 0.4 | 2 | 0.1 | 6.26E-01 | decreasing | **1.77E-02** | **decreasing** | 1 | 0.1 | 0 | 0.0 | 1.00 |  |
| 7F | 103 | 6.6 | 184 | 9.5 | 262 | 8.8 | **2.36E-03** | **increasing** | 4.17E-01 | decreasing | 102 | 12.7 | 29 | 4.1 | **1.26E-09** | **decreasing** |
| PCV10 | 870 | 56.1 | 792 | 40.8 | 621 | 20.9 | **3.56E-19** | **decreasing** | **9.60E-51** | **decreasing** | 252 | 31.5 | 81 | 11.4 | **9.11E-22** | **decreasing** |
| PCV10non7 | 195 | 12.6 | 306 | 15.8 | 382 | 12.8 | **7.54E-03** | **increasing** | **4.22E-03** | **decreasing** | 150 | 18.7 | 47 | 6.6 | **8.81E-13** | **decreasing** |
| 3 | 163 | 10.5 | 296 | 15.2 | 460 | 15.4 | **3.49E-05** | **increasing** | 8.72E-01 | increasing | 124 | 15.5 | 126 | 17.7 | 2.67E-01 | increasing |
| 6A | 58 | 3.7 | 74 | 3.8 | 44 | 1.5 | 9.29E-01 | increasing | **2.84E-07** | **decreasing** | 17 | 2.1 | 7 | 1.0 | 9.84E-02 | decreasing |
| 19A | 47 | 3.0 | 127 | 6.5 | 312 | 10.5 | **1.48E-06** | **increasing** | **1.80E-06** | **increasing** | 87 | 10.9 | 49 | 6.9 | **6.89E-03** | **decreasing** |
| PCV13 | 1138 | 73.3 | 1289 | 66.4 | 1437 | 48.3 | **1.07E-05** | **decreasing** | **3.02E-36** | **decreasing** | 480 | 59.9 | 263 | 36.9 | **3.61E-19** | **decreasing** |
| PCV13non7 | 463 | 29.8 | 803 | 41.4 | 1198 | 40.2 | **1.68E-12** | **increasing** | 4.40E-01 | decreasing | 378 | 47.2 | 229 | 32.1 | **2.70E-09** | **decreasing** |
| PCV13non10 | 268 | 17.3 | 497 | 25.6 | 816 | 27.4 | **2.83E-09** | **increasing** | 1.66E-01 | increasing | 228 | 28.5 | 182 | 25.5 | 2.03E-01 | decreasing |
| 2 | 1 | 0.1 | 1 | 0.1 | 0 | 0.0 | 1.00 |  | 3.95E-01 | decreasing | 0 | 0.0 | 0 | 0.0 | 1.00 |  |
| 8 | 60 | 3.9 | 61 | 3.1 | 129 | 4.3 | 2.64E-01 | decreasing | **3.43E-02** | **increasing** | 23 | 2.9 | 47 | 6.6 | **8.15E-04** | **increasing** |
| 9N | 35 | 2.3 | 56 | 2.9 | 85 | 2.9 | 2.85E-01 | increasing | 1.00 |  | 18 | 2.2 | 28 | 3.9 | 7.11E-02 | increasing |
| 10A | 32 | 2.1 | 47 | 2.4 | 76 | 2.6 | 4.94E-01 | increasing | 8.52E-01 | increasing | 17 | 2.1 | 21 | 2.9 | 3.27E-01 | increasing |
| 11A | 34 | 2.2 | 38 | 2.0 | 74 | 2.5 | 6.34E-01 | decreasing | 2.42E-01 | increasing | 17 | 2.1 | 21 | 2.9 | 3.27E-01 | increasing |
| 12F | 38 | 2.4 | 34 | 1.8 | 148 | 5.0 | 1.53E-01 | decreasing | **1.47E-09** | **increasing** | 33 | 4.1 | 44 | 6.2 | 7.88E-02 | increasing |
| 15B | 14 | 0.9 | 19 | 1.0 | 33 | 1.1 | 8.62E-01 | increasing | 7.76E-01 | increasing | 6 | 0.7 | 9 | 1.3 | 4.37E-01 | increasing |
| 17F | 10 | 0.6 | 3 | 0.2 | 23 | 0.8 | **2.36E-02** | **decreasing** | **3.84E-03** | **increasing** | 4 | 0.5 | 7 | 1.0 | 3.66E-01 | increasing |
| 20 | 8 | 0.5 | 10 | 0.5 | 22 | 0.7 | 1.00 |  | 3.71E-01 | increasing | 9 | 1.1 | 5 | 0.7 | 4.33E-01 | decreasing |
| 22F | 26 | 1.7 | 95 | 4.9 | 232 | 7.8 | **1.15E-07** | **increasing** | **6.46E-05** | **increasing** | 61 | 7.6 | 67 | 9.4 | 2.29E-01 | increasing |
| 33F | 9 | 0.6 | 26 | 1.3 | 54 | 1.8 | **2.63E-02** | **increasing** | 2.07E-01 | increasing | 13 | 1.6 | 18 | 2.5 | 2.75E-01 | increasing |
| PPV23 | 1347 | 86.8 | 1605 | 82.7 | 2269 | 76.2 | **9.74E-04** | **decreasing** | **4.64E-08** | **decreasing** | 664 | 82.9 | 523 | 73.4 | **8.38E-06** | **decreasing** |
| 6C | 15 | 1.0 | 34 | 1.8 | 88 | 3.0 | 5.92E-02 | increasing | **8.42E-03** | **increasing** | 27 | 3.4 | 15 | 2.1 | 1.59E-01 | decreasing |
| 6D | 0 | 0.0 | 2 | 0.1 | 0 | 0.0 | 5.06E-01 | increasing | 1.56E-01 | decreasing | 0 | 0.0 | 0 | 0.0 | 1.00 |  |
| 7B | 0 | 0.0 | 4 | 0.2 | 0 | 0.0 | 1.34E-01 | increasing | **2.42E-02** | **decreasing** | 0 | 0.0 | 0 | 0.0 | 1.00 |  |
| 7C | 0 | 0.0 | 3 | 0.2 | 2 | 0.1 | 2.59E-01 | increasing | 3.89E-01 | decreasing | 0 | 0.0 | 1 | 0.1 | 4.71E-01 | increasing |
| 9A | 9 | 0.6 | 3 | 0.2 | 0 | 0.0 | 4.14E-02 | decreasing | 6.14E-02 | decreasing | 0 | 0.0 | 0 | 0.0 | 1.00 |  |
| 9L | 0 | 0.0 | 1 | 0.1 | 0 | 0.0 | 1.00 |  | 3.95E-01 | decreasing | 0 | 0.0 | 0 | 0.0 | 1.00 |  |
| 6F | 0 | 0.0 | 0 | 0.0 | 1 | 0.0 | 1.00 |  | 1.00 |  | 1 | 0.1 | 0 | 0.0 | 1.00 |  |
| 10B | 2 | 0.1 | 2 | 0.1 | 3 | 0.1 | 1.00 |  | 1.00 |  | 2 | 0.2 | 0 | 0.0 | 5.01E-01 | decreasing |
| 10F | 1 | 0.1 | 1 | 0.1 | 1 | 0.0 | 1.00 |  | 1.00 |  | 0 | 0.0 | 0 | 0.0 | 1.00 |  |
| 11B | 1 | 0.1 | 0 | 0.0 | 0 | 0.0 | 4.44E-01 | decreasing | 1.00 |  | 0 | 0.0 | 0 | 0.0 | 1.00 |  |
| 11C | 0 | 0.0 | 0 | 0.0 | 1 | 0.0 | 1.00 |  | 1.00 |  | 0 | 0.0 | 0 | 0.0 | 1.00 |  |
| 11F | 0 | 0.0 | 1 | 0.1 | 0 | 0.0 | 1.00 |  | 3.95E-01 | decreasing | 0 | 0.0 | 0 | 0.0 | 1.00 |  |
| 12A | 0 | 0.0 | 1 | 0.1 | 6 | 0.2 | 1.00 |  | 2.57E-01 | increasing | 0 | 0.0 | 4 | 0.6 | **4.90E-02** | **increasing** |
| 13 | 5 | 0.3 | 5 | 0.3 | 1 | 0.0 | 7.59E-01 | decreasing | **3.84E-02** | **decreasing** | 0 | 0.0 | 0 | 0.0 | 1.00 |  |
| 15A | 5 | 0.3 | 14 | 0.7 | 84 | 2.8 | 1.63E-01 | increasing | **5.25E-08** | **increasing** | 12 | 1.5 | 32 | 4.5 | **6.16E-04** | **increasing** |
| 15C | 11 | 0.7 | 5 | 0.3 | 22 | 0.7 | 7.46E-02 | decreasing | **2.88E-02** | **increasing** | 4 | 0.5 | 3 | 0.4 | 1.00 |  |
| 15F | 3 | 0.2 | 0 | 0.0 | 0 | 0.0 | 8.76E-02 | decreasing | 1.00 |  | 0 | 0.0 | 0 | 0.0 | 1.00 |  |
| 16F | 4 | 0.3 | 8 | 0.4 | 25 | 0.8 | 5.66E-01 | increasing | 7.64E-02 | increasing | 3 | 0.4 | 5 | 0.7 | 4.87E-01 | increasing |
| 17A | 1 | 0.1 | 0 | 0.0 | 0 | 0.0 | 4.44E-01 | decreasing | 1.00 |  | 0 | 0.0 | 0 | 0.0 | 1.00 |  |
| 18A | 3 | 0.2 | 3 | 0.2 | 6 | 0.2 | 1.00 |  | 1.00 |  | 0 | 0.0 | 0 | 0.0 | 1.00 |  |
| 18B | 0 | 0.0 | 0 | 0.0 | 1 | 0.0 | 1.00 |  | 1.00 |  | 0 | 0.0 | 0 | 0.0 | 1.00 |  |
| 18F | 1 | 0.1 | 0 | 0.0 | 0 | 0.0 | 4.44E-01 | decreasing | 1.00 |  | 0 | 0.0 | 0 | 0.0 | 1.00 |  |
| 19C | 1 | 0.1 | 0 | 0.0 | 0 | 0.0 | 4.44E-01 | decreasing | 1.00 |  | 0 | 0.0 | 0 | 0.0 | 1.00 |  |
| 21 | 0 | 0.0 | 0 | 0.0 | 1 | 0.0 | 1.00 |  | 1.00 |  | 0 | 0.0 | 1 | 0.1 | 4.71E-01 | increasing |
| 22A | 1 | 0.1 | 0 | 0.0 | 1 | 0.0 | 4.44E-01 | decreasing | 1.00 |  | 1 | 0.1 | 0 | 0.0 | 1.00 |  |
| 23A | 15 | 1.0 | 32 | 1.6 | 74 | 2.5 | 1.03E-01 | increasing | 5.59E-02 | increasing | 16 | 2.0 | 18 | 2.5 | 4.94E-01 | increasing |
| 23B | 3 | 0.2 | 13 | 0.7 | 83 | 2.8 | **4.40E-02** | **increasing** | **3.34E-08** | **increasing** | 11 | 1.4 | 27 | 3.8 | **2.89E-03** | **increasing** |
| 24A | 1 | 0.1 | 0 | 0.0 | 1 | 0.0 | 4.44E-01 | decreasing | 1.00 |  | 0 | 0.0 | 0 | 0.0 | 1.00 |  |
| 24B | 1 | 0.1 | 0 | 0.0 | 0 | 0.0 | 4.44E-01 | decreasing | 1.00 |  | 0 | 0.0 | 0 | 0.0 | 1.00 |  |
| 24F | 17 | 1.1 | 25 | 1.3 | 74 | 2.5 | 6.42E-01 | increasing | **3.46E-03** | **increasing** | 7 | 0.9 | 28 | 3.9 | **9.62E-05** | **increasing** |
| 25A | 0 | 0.0 | 2 | 0.1 | 0 | 0.0 | 5.06E-01 | increasing | 1.56E-01 | decreasing | 0 | 0.0 | 0 | 0.0 | 1.00 |  |
| 25F | 1 | 0.1 | 1 | 0.1 | 0 | 0.0 | 1.00 |  | 3.95E-01 | decreasing | 0 | 0.0 | 0 | 0.0 | 1.00 |  |
| 28A | 1 | 0.1 | 1 | 0.1 | 5 | 0.2 | 1.00 |  | 4.13E-01 | increasing | 2 | 0.2 | 0 | 0.0 | 5.01E-01 | decreasing |
| 28F | 1 | 0.1 | 1 | 0.1 | 3 | 0.1 | 1.00 |  | 1.00 |  | 2 | 0.2 | 0 | 0.0 | 5.01E-01 | decreasing |
| 29 | 1 | 0.1 | 1 | 0.1 | 4 | 0.1 | 1.00 |  | 6.54E-01 | increasing | 1 | 0.1 | 1 | 0.1 | 1.00 |  |
| 31 | 8 | 0.5 | 14 | 0.7 | 41 | 1.4 | 5.22E-01 | increasing | **3.69E-02** | **increasing** | 5 | 0.6 | 10 | 1.4 | 1.92E-01 | increasing |
| 33A | 4 | 0.3 | 4 | 0.2 | 0 | 0.0 | 7.40E-01 | decreasing | **2.42E-02** | **decreasing** | 0 | 0.0 | 0 | 0.0 | 1.00 |  |
| 33B | 0 | 0.0 | 1 | 0.1 | 1 | 0.0 | 1.00 |  | 1.00 |  | 0 | 0.0 | 0 | 0.0 | 1.00 |  |
| 34 | 7 | 0.5 | 10 | 0.5 | 15 | 0.5 | 1.00 |  | 1.00 |  | 4 | 0.5 | 3 | 0.4 | 1.00 |  |
| 35A | 1 | 0.1 | 2 | 0.1 | 0 | 0.0 | 1.00 |  | 1.56E-01 | decreasing | 0 | 0.0 | 0 | 0.0 | 1.00 |  |
| 35B | 0 | 0.0 | 12 | 0.6 | 38 | 1.3 | **9.12E-04** | **increasing** | **2.84E-02** | **increasing** | 5 | 0.6 | 12 | 1.7 | **8.42E-02** | **increasing** |
| 35C | 1 | 0.1 | 0 | 0.0 | 1 | 0.0 | 4.44E-01 | decreasing | 1.00 |  | 0 | 0.0 | 0 | 0.0 | 1.00 |  |
| 35F | 8 | 0.5 | 29 | 1.5 | 43 | 1.4 | **4.53E-03** | **increasing** | 9.04E-01 | decreasing | 9 | 1.1 | 14 | 2.0 | 2.10E-01 | increasing |
| 36 | 1 | 0.1 | 0 | 0.0 | 0 | 0.0 | 4.44E-01 | decreasing | 1.00 |  | 0 | 0.0 | 0 | 0.0 | 1.00 |  |
| 37 | 2 | 0.1 | 0 | 0.0 | 3 | 0.1 | 1.97E-01 | decreasing | 2.83E-01 | increasing | 0 | 0.0 | 1 | 0.1 | 4.71E-01 | increasing |
| 38 | 7 | 0.5 | 23 | 1.2 | 33 | 1.1 | **2.53E-02** | **increasing** | 7.86E-01 | decreasing | 8 | 1.0 | 7 | 1.0 | 1.00 |  |
| 48 | 1 | 0.1 | 0 | 0.0 | 0 | 0.0 | 4.44E-01 | decreasing | 1.00 |  | 0 | 0.0 | 0 | 0.0 | 1.00 |  |
| NT | 3 | 0.2 | 4 | 0.2 | 3 | 0.1 | 1.00 |  | 4.45E-01 | decreasing | 0 | 0.0 | 1 | 0.1 | 4.71E-01 | increasing |
| nonPCV13 | 414 | 26.7 | 652 | 33.6 | 1541 | 51.7 | **1.07E-05** | **increasing** | **3.02E-36** | **increasing** | 321 | 40.1 | 450 | 63.1 | **3.61E-19** | **increasing** |
| total | 1552 | 100.0 | 1941 | 100.0 | 2978 | 100.0 |  |  |  |  | 801 | 100.0 | 713 | 100.0 |  |  |

**Table I: Serotype distribution among isolates from IPD in adults (>75 years of age) in Germany (n=5,972)**.

| **Serotype** | **Pre-Vaccination 1992-2006** | | **Early Vaccination 2007-2010** | | **Late Vaccination 2010-2014** | | **Pre-Vaccination vs. Early Vaccination** | | **Early Vaccination vs. Late Vaccination** | | **2010-2011** | | **2013-2014** | | **2010-2011 vs. 2013-2014** | |
| --- | --- | --- | --- | --- | --- | --- | --- | --- | --- | --- | --- | --- | --- | --- | --- | --- |
|  | **n** | **%** | **n** | **%** | **n** | **%** | **p-value** | **direction** | **p-value** | **direction** | **n** | **%** | **n** | **%** | **p-value** | **direction** |
| 4 | 80 | 6.9 | 76 | 4.5 | 38 | 1.4 | **7.15E-03** | **decreasing** | **4.28E-10** | **decreasing** | 14 | 2.1 | 8 | 1.2 | 2.82E-01 | decreasing |
| 6B | 48 | 4.1 | 43 | 2.5 | 19 | 0.7 | **2.23E-02** | **decreasing** | **5.07E-07** | **decreasing** | 5 | 0.7 | 5 | 0.7 | 1.00 |  |
| 9V | 74 | 6.4 | 58 | 3.4 | 19 | 0.7 | **3.67E-04** | **decreasing** | **1.94E-11** | **decreasing** | 12 | 1.8 | 2 | 0.3 | **1.25E-02** | **decreasing** |
| 14 | 185 | 15.9 | 120 | 7.1 | 41 | 1.5 | **1.17E-13** | **decreasing** | **9.51E-22** | **decreasing** | 12 | 1.8 | 7 | 1.0 | 3.56E-01 | decreasing |
| 18C | 25 | 2.2 | 32 | 1.9 | 29 | 1.0 | 6.83E-01 | decreasing | **2.32E-02** | **decreasing** | 13 | 1.9 | 6 | 0.9 | 1.64E-01 | decreasing |
| 19F | 33 | 2.8 | 63 | 3.7 | 49 | 1.8 | 2.07E-01 | increasing | **7.02E-05** | **decreasing** | 19 | 2.8 | 10 | 1.5 | 1.32E-01 | decreasing |
| 23F | 95 | 8.2 | 73 | 4.3 | 42 | 1.5 | **2.26E-05** | **decreasing** | **2.46E-08** | **decreasing** | 13 | 1.9 | 3 | 0.4 | **2.06E-02** | **decreasing** |
| PCV7 | 540 | 46.5 | 465 | 27.5 | 237 | 8.5 | **3.06E-25** | **decreasing** | **8.17E-62** | **decreasing** | 88 | 12.9 | 41 | 6.1 | **1.75E-05** | **decreasing** |
| 1 | 40 | 3.4 | 66 | 3.9 | 42 | 1.5 | 5.47E-01 | increasing | **9.42E-07** | **decreasing** | 17 | 2.5 | 5 | 0.7 | **1.61E-02** | **decreasing** |
| 5 | 4 | 0.3 | 1 | 0.1 | 1 | 0.0 | 1.65E-01 | decreasing | 1.00 |  | 1 | 0.1 | 0 | 0.0 | 1.00 |  |
| 7F | 62 | 5.3 | 124 | 7.3 | 193 | 7.0 | **3.71E-02** | **increasing** | 6.74E-01 | decreasing | 61 | 9.0 | 34 | 5.0 | **5.46E-03** | **decreasing** |
| PCV10 | 646 | 55.6 | 656 | 38.7 | 473 | 17.0 | **8.19E-19** | **decreasing** | **1.40E-57** | **decreasing** | 167 | 24.6 | 80 | 11.9 | **1.12E-09** | **decreasing** |
| PCV10non7 | 106 | 9.1 | 191 | 11.3 | 236 | 8.5 | 7.03E-02 | increasing | **2.76E-03** | **decreasing** | 79 | 11.6 | 39 | 5.8 | **1.54E-04** | **decreasing** |
| 3 | 120 | 10.3 | 269 | 15.9 | 438 | 15.8 | **1.83E-05** | **increasing** | 9.33E-01 | decreasing | 103 | 15.1 | 104 | 15.4 | 9.40E-01 | increasing |
| 6A | 43 | 3.7 | 63 | 3.7 | 49 | 1.8 | 1.00 |  | **7.02E-05** | **decreasing** | 8 | 1.2 | 10 | 1.5 | 6.44E-01 | increasing |
| 19A | 37 | 3.2 | 111 | 6.6 | 288 | 10.4 | **4.85E-05** | **increasing** | **1.12E-05** | **increasing** | 88 | 12.9 | 59 | 8.7 | **1.43E-02** | **decreasing** |
| PCV13 | 846 | 72.8 | 1099 | 64.9 | 1248 | 45.0 | **8.31E-06** | **decreasing** | **1.00E-38** | **decreasing** | 366 | 53.8 | 253 | 37.5 | **1.84E-09** | **decreasing** |
| PCV13non7 | 306 | 26.3 | 634 | 37.4 | 1011 | 36.4 | **5.20E-10** | **increasing** | 5.02E-01 | decreasing | 278 | 40.9 | 212 | 31.4 | **2.96E-04** | **decreasing** |
| PCV13non10 | 200 | 17.2 | 443 | 26.2 | 775 | 27.9 | **1.45E-08** | **increasing** | 2.13E-01 | increasing | 199 | 29.3 | 173 | 25.6 | 1.44E-01 | decreasing |
| 2 | 0 | 0.0 | 4 | 0.2 | 0 | 0.0 | 1.51E-01 | increasing | **2.06E-02** | **decreasing** | 0 | 0.0 | 0 | 0.0 | 1.00 |  |
| 8 | 29 | 2.5 | 34 | 2.0 | 77 | 2.8 | 4.37E-01 | decreasing | 1.14E-01 | increasing | 16 | 2.4 | 21 | 3.1 | 4.10E-01 | increasing |
| 9N | 40 | 3.4 | 65 | 3.8 | 122 | 4.4 | 6.14E-01 | increasing | 3.97E-01 | increasing | 29 | 4.3 | 30 | 4.4 | 8.95E-01 | increasing |
| 10A | 13 | 1.1 | 34 | 2.0 | 84 | 3.0 | 7.30E-02 | increasing | **4.32E-02** | **increasing** | 15 | 2.2 | 24 | 3.6 | 1.47E-01 | increasing |
| 11A | 25 | 2.2 | 46 | 2.7 | 88 | 3.2 | 3.92E-01 | increasing | 4.17E-01 | increasing | 19 | 2.8 | 19 | 2.8 | 1.00 |  |
| 12F | 27 | 2.3 | 24 | 1.4 | 127 | 4.6 | 8.42E-02 | decreasing | **3.30E-09** | **increasing** | 18 | 2.6 | 46 | 6.8 | **2.94E-04** | **increasing** |
| 15B | 5 | 0.4 | 21 | 1.2 | 38 | 1.4 | **2.70E-02** | **increasing** | 7.88E-01 | increasing | 10 | 1.5 | 9 | 1.3 | 1.00 |  |
| 17F | 8 | 0.7 | 5 | 0.3 | 19 | 0.7 | 1.59E-01 | decreasing | 9.40E-02 | increasing | 2 | 0.3 | 5 | 0.7 | 2.86E-01 | increasing |
| 20 | 13 | 1.1 | 8 | 0.5 | 25 | 0.9 | 7.18E-02 | decreasing | 1.48E-01 | increasing | 5 | 0.7 | 6 | 0.9 | 7.73E-01 | increasing |
| 22F | 35 | 3.0 | 71 | 4.2 | 187 | 6.7 | 1.08E-01 | increasing | **3.46E-04** | **increasing** | 47 | 6.9 | 45 | 6.7 | 9.14E-01 | decreasing |
| 33F | 11 | 0.9 | 16 | 0.9 | 53 | 1.9 | 1.00 |  | **1.19E-02** | **increasing** | 19 | 2.8 | 11 | 1.6 | 1.96E-01 | decreasing |
| PPV23 | 1009 | 86.8 | 1364 | 80.6 | 2019 | 72.8 | **9.46E-06** | **decreasing** | **2.79E-09** | **decreasing** | 538 | 79.1 | 459 | 68.0 | **3.67E-06** | **decreasing** |
| 6C | 11 | 0.9 | 44 | 2.6 | 133 | 4.8 | **1.30E-03** | **increasing** | **2.55E-04** | **increasing** | 26 | 3.8 | 27 | 4.0 | 8.89E-01 | increasing |
| 6D | 0 | 0.0 | 0 | 0.0 | 2 | 0.1 | 1.00 |  | 5.29E-01 | increasing | 1 | 0.1 | 0 | 0.0 | 1.00 |  |
| 7B | 1 | 0.1 | 0 | 0.0 | 0 | 0.0 | 4.07E-01 | decreasing | 1.00 |  | 0 | 0.0 | 0 | 0.0 | 1.00 |  |
| 7C | 2 | 0.2 | 1 | 0.1 | 2 | 0.1 | 5.70E-01 | decreasing | 1.00 |  | 1 | 0.1 | 0 | 0.0 | 1.00 |  |
| 9A | 3 | 0.3 | 1 | 0.1 | 1 | 0.0 | 3.11E-01 | decreasing | 1.00 |  | 0 | 0.0 | 0 | 0.0 | 1.00 |  |
| 9L | 4 | 0.3 | 2 | 0.1 | 0 | 0.0 | 2.32E-01 | decreasing | 1.44E-01 | decreasing | 0 | 0.0 | 0 | 0.0 | 1.00 |  |
| 10B | 1 | 0.1 | 3 | 0.2 | 4 | 0.1 | 6.50E-01 | increasing | 1.00 |  | 0 | 0.0 | 2 | 0.3 | 2.48E-01 | increasing |
| 10F | 1 | 0.1 | 1 | 0.1 | 2 | 0.1 | 1.00 |  | 1.00 |  | 1 | 0.1 | 1 | 0.1 | 1.00 |  |
| 11B | 0 | 0.0 | 1 | 0.1 | 1 | 0.0 | 1.00 |  | 1.00 |  | 0 | 0.0 | 0 | 0.0 | 1.00 |  |
| 11F | 0 | 0.0 | 1 | 0.1 | 0 | 0.0 | 1.00 |  | 3.79E-01 | decreasing | 0 | 0.0 | 0 | 0.0 | 1.00 |  |
| 12A | 4 | 0.3 | 1 | 0.1 | 2 | 0.1 | 1.65E-01 | decreasing | 1.00 |  | 0 | 0.0 | 1 | 0.1 | 4.98E-01 | increasing |
| 12B | 0 | 0.0 | 3 | 0.2 | 0 | 0.0 | 2.76E-01 | increasing | 5.43E-02 | decreasing | 0 | 0.0 | 0 | 0.0 | 1.00 |  |
| 13 | 2 | 0.2 | 1 | 0.1 | 0 | 0.0 | 5.70E-01 | decreasing | 3.79E-01 | decreasing | 0 | 0.0 | 0 | 0.0 | 1.00 |  |
| 15A | 13 | 1.1 | 7 | 0.4 | 80 | 2.9 | **3.76E-02** | **decreasing** | **2.00E-10** | **increasing** | 9 | 1.3 | 29 | 4.3 | **8.74E-04** | **increasing** |
| 15C | 3 | 0.3 | 7 | 0.4 | 13 | 0.5 | 7.49E-01 | increasing | 1.00 |  | 5 | 0.7 | 3 | 0.4 | 7.26E-01 | decreasing |
| 15F | 1 | 0.1 | 1 | 0.1 | 1 | 0.0 | 1.00 |  | 1.00 |  | 0 | 0.0 | 1 | 0.1 | 4.98E-01 | increasing |
| 16F | 4 | 0.3 | 9 | 0.5 | 39 | 1.4 | 5.78E-01 | increasing | **6.47E-03** | **increasing** | 4 | 0.6 | 16 | 2.4 | **6.62E-03** | **increasing** |
| 18A | 5 | 0.4 | 4 | 0.2 | 4 | 0.1 | 5.00E-01 | decreasing | 4.87E-01 | decreasing | 0 | 0.0 | 2 | 0.3 | 2.48E-01 | increasing |
| 18B | 1 | 0.1 | 0 | 0.0 | 2 | 0.1 | 4.07E-01 | decreasing | 5.29E-01 | increasing | 1 | 0.1 | 0 | 0.0 | 1.00 |  |
| 18F | 1 | 0.1 | 1 | 0.1 | 0 | 0.0 | 1.00 |  | 3.79E-01 | decreasing | 0 | 0.0 | 0 | 0.0 | 1.00 |  |
| 19B | 1 | 0.1 | 0 | 0.0 | 0 | 0.0 | 4.07E-01 | decreasing | 1.00 |  | 0 | 0.0 | 0 | 0.0 | 1.00 |  |
| 19C | 1 | 0.1 | 0 | 0.0 | 0 | 0.0 | 4.07E-01 | decreasing | 1.00 |  | 0 | 0.0 | 0 | 0.0 | 1.00 |  |
| 21 | 0 | 0.0 | 0 | 0.0 | 2 | 0.1 | 1.00 |  | 5.29E-01 | increasing | 0 | 0.0 | 0 | 0.0 | 1.00 |  |
| 23A | 5 | 0.4 | 34 | 2.0 | 81 | 2.9 | **2.17E-04** | **increasing** | 6.46E-02 | increasing | 14 | 2.1 | 29 | 4.3 | **2.02E-02** | **increasing** |
| 23B | 3 | 0.3 | 8 | 0.5 | 47 | 1.7 | 5.41E-01 | increasing | **2.11E-04** | **increasing** | 4 | 0.6 | 15 | 2.2 | **1.10E-02** | **increasing** |
| 24A | 0 | 0.0 | 0 | 0.0 | 1 | 0.0 | 1.00 |  | 1.00 |  | 0 | 0.0 | 0 | 0.0 | 1.00 |  |
| 24B | 0 | 0.0 | 1 | 0.1 | 0 | 0.0 | 1.00 |  | 3.79E-01 | decreasing | 0 | 0.0 | 0 | 0.0 | 1.00 |  |
| 24F | 14 | 1.2 | 32 | 1.9 | 82 | 3.0 | 1.75E-01 | increasing | **3.12E-02** | **increasing** | 17 | 2.5 | 30 | 4.4 | 5.43E-02 | increasing |
| 27 | 0 | 0.0 | 0 | 0.0 | 1 | 0.0 | 1.00 |  | 1.00 |  | 1 | 0.1 | 0 | 0.0 | 1.00 |  |
| 28A | 0 | 0.0 | 6 | 0.4 | 4 | 0.1 | 8.74E-02 | increasing | 1.93E-01 | decreasing | 3 | 0.4 | 0 | 0.0 | 2.49E-01 | decreasing |
| 28F | 0 | 0.0 | 0 | 0.0 | 4 | 0.1 | 1.00 |  | 3.04E-01 | increasing | 0 | 0.0 | 2 | 0.3 | 2.48E-01 | increasing |
| 29 | 0 | 0.0 | 1 | 0.1 | 2 | 0.1 | 1.00 |  | 1.00 |  | 1 | 0.1 | 0 | 0.0 | 1.00 |  |
| 31 | 5 | 0.4 | 12 | 0.7 | 39 | 1.4 | 4.60E-01 | increasing | **4.11E-02** | **increasing** | 10 | 1.5 | 11 | 1.6 | 8.30E-01 | increasing |
| 33A | 2 | 0.2 | 1 | 0.1 | 0 | 0.0 | 5.70E-01 | decreasing | 3.79E-01 | decreasing | 0 | 0.0 | 0 | 0.0 | 1.00 |  |
| 33B | 1 | 0.1 | 0 | 0.0 | 0 | 0.0 | 4.07E-01 | decreasing | 1.00 |  | 0 | 0.0 | 0 | 0.0 | 1.00 |  |
| 34 | 2 | 0.2 | 7 | 0.4 | 14 | 0.5 | 3.25E-01 | increasing | 8.23E-01 | increasing | 5 | 0.7 | 3 | 0.4 | 7.26E-01 | decreasing |
| 35A | 0 | 0.0 | 1 | 0.1 | 0 | 0.0 | 1.00 |  | 3.79E-01 | decreasing | 0 | 0.0 | 0 | 0.0 | 1.00 |  |
| 35B | 3 | 0.3 | 12 | 0.7 | 40 | 1.4 | 1.19E-01 | increasing | **3.03E-02** | **increasing** | 11 | 1.6 | 10 | 1.5 | 1.00 |  |
| 35C | 2 | 0.2 | 1 | 0.1 | 4 | 0.1 | 5.70E-01 | decreasing | 6.56E-01 | increasing | 0 | 0.0 | 1 | 0.1 | 4.98E-01 | increasing |
| 35F | 6 | 0.5 | 29 | 1.7 | 53 | 1.9 | **4.85E-03** | **increasing** | 7.31E-01 | increasing | 12 | 1.8 | 10 | 1.5 | 8.30E-01 | decreasing |
| 37 | 1 | 0.1 | 0 | 0.0 | 1 | 0.0 | 4.07E-01 | decreasing | 1.00 |  | 1 | 0.1 | 0 | 0.0 | 1.00 |  |
| 38 | 7 | 0.6 | 31 | 1.8 | 46 | 1.7 | **4.38E-03** | **increasing** | 7.22E-01 | decreasing | 7 | 1.0 | 13 | 1.9 | 1.85E-01 | increasing |
| 39 | 0 | 0.0 | 1 | 0.1 | 0 | 0.0 | 1.00 |  | 3.79E-01 | decreasing | 0 | 0.0 | 0 | 0.0 | 1.00 |  |
| NT | 0 | 0.0 | 1 | 0.1 | 0 | 0.0 | 1.00 |  | 3.79E-01 | decreasing | 0 | 0.0 | 0 | 0.0 | 1.00 |  |
| nonPCV13 | 316 | 27.2 | 594 | 35.1 | 1527 | 55.0 | **8.31E-06** | **increasing** | **1.00E-38** | **increasing** | 314 | 46.2 | 422 | 62.5 | **1.84E-09** | **increasing** |
| total | 1162 | 100.0 | 1693 | 100.0 | 2775 | 100.0 |  |  |  |  | 680 | 100.0 | 675 | 100.0 |  |  |
